# Foundations of Community Engagement: A Series for Effective Community-Engaged Research

**DOI:** 10.15766/mep_2374-8265.11350

**Published:** 2023-10-10

**Authors:** Bryan Johnston, Leslie Ruffalo, David Nelson, Sarah O'Connor, Staci Young

**Affiliations:** 1 Assistant Professor, Department of Family and Community Medicine, Medical College of Wisconsin; 2 Associate Professor, Department of Family and Community Medicine, Medical College of Wisconsin; 3 Professor, Department of Family and Community Medicine, Medical College of Wisconsin; 4 Program Director, Office of Community Engagement, Medical College of Wisconsin; 5 Professor, Department of Family and Community Medicine, Director, Office of Community Engagement, and Senior Associate Dean for Community Engagement, Medical College of Wisconsin

**Keywords:** Community Engagement, Community-Based Medicine, Diversity, Equity, Inclusion

## Abstract

**Introduction:**

Medical students lack systematic exposure to community engagement. Community-engaged research (CEnR) is an effective approach to improve community health, and community-engaged physicians are better attuned to the community context of their patients' health and well-being. The Medical College of Wisconsin (MCW) Office of Community Engagement began offering the educational series Foundations of Community Engagement in 2021 to meet this need.

**Methods:**

We developed and implemented a four-session series for medical students at MCW and the University of Nebraska Medical Center. A 1-hour session on the foundations of CEnR was held for all learners. Three 1-hour sessions dove deeper into CEnR principles for a self-selected cohort. These small-group sessions involved discussion between faculty and community partners and facilitated small-group discussion. Students completed evaluations after each session.

**Results:**

A total of 160 students participated in the introductory session; 36 took part in the follow-up series. Survey response rates varied from 38% to 67% for each session. Overall, 87% of students in all sessions felt their session was worthwhile, with 85% of large-group and 96% of small-group respondents reporting they learned something they would use in their practice or profession. Qualitative responses included appreciation for addressing a curricular gap and desire for more time and more sessions to continue discussions.

**Discussion:**

The program was effective at stimulating medical student self-reported gains in skills, attitudes, and future intentions regarding CEnR in an efficient manner. Effective programs that transfer positive CEnR skills and attitudes to future physicians can promote CEnR within academic medicine.

## Educational Objectives

By the end of this activity, learners will be able to:

1. Define the principles of community-engaged research (CEnR).
2. Apply the principles of CEnR to current/future CEnR opportunities.
3. Discuss examples of the principles of CEnR.

## Introduction

While US academic medical institutions have traditionally connected their student body to community service opportunities,^1^ medical students lack substantial exposure to higher-level community engagement and community-engaged research (CEnR) skills.^2^ CEnR skills differ from traditional research skills in that they involve “working collaboratively with groups of people who are affiliated by geographic proximity, special interests, or similar situations with respect to issues affecting their well-being”^3^ and may include community-engaged education, clinical care, research, and policy/advocacy work.^4^ This provides an opportunity for improvement, as CEnR activities are associated with improved health outcomes, especially for vulnerable communities.^5–9^ In particular, CEnR is associated with improved health-seeking behaviors, which contribute to improving positive health attainment alongside prevention of negative health behaviors.^6,7^ The critical role of CEnR in promoting community health is particularly resonant for vulnerable communities during times of health crisis, such as the COVID-19 pandemic.^10^ Community-engaged clinicians are more aware of the context of their patients' health, are better able to identify social determinants of health needs and to connect to appropriate interventions, feel more capable of meeting the needs of their patients, and report lower levels of burnout and improved wellness.^7,11,12^ Applying CEnR principles to patient care also helps clinicians build trust and identify and recommend behavioral health interventions to improve their patients' health and wellness. In a sense, connecting to community context in a substantial way prior to or alongside traditional biomedical-focused medical education insulates medical learners from losing track of important community- and individual-level behavior changes that are estimated to be even more influential upon one's overall health than the accumulated impact of health care.^13^

To date, the literature has focused on development of effective researcher- and faculty-centered CEnR skill transmission.^14–18^ Medical learners face additional challenges to engaging in substantial CEnR skill development, including competing priorities and expectations, the staggered nature of their curricula, scheduling inflexibility, and lack of access to mentors and institutional support to enable experiences involving effective higher-level CEnR activities. Existing learner-focused curricula tend to concentrate on one aspect of CEnR, such as adding scholarly components to community service work,^19^ community health education,^20^ or understanding and facilitating clinical–community connections to intervene in deficiencies in the social determinants of health.^21^ Perhaps owing to these challenges, as well as the relatively recent recognition of the importance of CEnR as part of the mission of academic medical institutions,^17^ there is a lack of publications demonstrating efficient, effective means to transfer CEnR principles to medical students.

The Medical College of Wisconsin (MCW) is recognized as a leader in CEnR and has established community engagement as the institution's fourth mission (alongside clinical care, education, and research). The institution boasts substantial CEnR activities and supports faculty, staff, and learners to engage in these areas through a variety of means. For medical students, this has often taken the form of choosing to dedicate nonclassroom education time to CEnR education through a longitudinal pathways program or summer research opportunity between the first and second years of medical school. This approach was effective at providing rich CEnR learning experiences for those able to access it—a subset of the overall cadre of future physicians, which left out many students interested in CEnR who had, due to limited options or competing priorities, found this time dedicated to other directions. Still others were unfamiliar with CEnR or deprioritized it in favor of other interests and were unlikely to encounter CEnR in a substantial way throughout their training.

Students should be exposed to CEnR early in their training in order to provide a general understanding of CEnR and to act as a springboard to inspire interested students to engage more substantially in learning and applying CEnR principles in their careers. Limited CEnR curricula exist for medical student education, creating the need to develop a curriculum to address these goals. Key curricular aspects include teaching the breadth of CEnR via the five core community engagement principles, integrating community partner voices, case-based teaching, and incorporation of interactive components such as audience response and small-group discussion. The first iteration of this curriculum was undertaken in a virtual format in 2020. The curriculum was honed based on feedback from 2020 and again held in a virtual format in 2021. The results reported here and the curricular materials shared in the appendices reflect the 2021 curriculum, which is suitable for either virtual or in-person formats.

## Methods

### Curriculum Development

Faculty and staff in the Office of Community Engagement at MCW developed the Foundations of Community Engagement series. We conducted a literature review and enlisted expert opinions to identify appropriate priorities for CEnR knowledge and skill focus for medical learners. We assessed our local medical school curriculum to identify engagement opportunities, settling on the summer research program that annually engaged approximately three-quarters of the medical school class between their first and second years. We designed a multistage curriculum so that all students would be oriented in conceptual understanding of CEnR in a large-group session and could then apply to an optional, small-group, three-session series. As an institutional partner of our office, the University of Nebraska Medical Center (UNMC) was invited to participate in the program. Our curriculum focused on CEnR principles, with the first large-group session providing an overview of the five CEnR principles and subsequent small-group sessions centering on one or two of them in greater depth. The small-group sessions highlighted community and learner voices alongside their faculty partners/teachers in the context of a real-world CEnR project and provided space for guided interactive participant discussion. Feedback from the 2020 session allowed development and honing of our approach and curriculum in the 2021 session. The MCW Institutional Review Board oversaw the project, and we developed and administered postsession evaluations that included both qualitative and quantitative data elements.

### Curriculum Description and Implementation

The Foundations of Community Engagement series included four sessions over two parts. We used the article entitled “Community Engagement in Research: Frameworks for Education and Peer Review,” by Ahmed and Palermo,^22^ as the framework for all sessions. The first part of the series involved a didactic session in which CEnR was introduced conceptually to medical students enrolled in the summer research program (Appendix A). This 1-hour session introduced learners to core principles of community engagement referenced by Ahmed and Palermo, including (1) definition and scope of community engagement, (2) strong community–academic partnership, (3) equitable power and responsibility, (4) capacity building, and (5) effective dissemination plan. Given the breadth of content and the introductory nature of this session, we used a didactic format with interactive polling and opportunities for comments and questions. We recommend that future implementations of this CEnR orientation be led by a faculty member with experience and expertise in CEnR. In our case, the orientation was led by the institution's senior associate dean for community engagement. The session also included time for two additional community-engaged faculty to provide personal perspectives and examples of applying CEnR principles to their research and engaging medical students in this work.

For the second part, we invited both the MCW and UNMC student groups to apply to a three-session course in which we explored core CEnR principles in more depth. The application process provided an opportunity to benchmark the CEnR experiences that our learners had had before our series. No prerequisite CEnR knowledge or experience was required, and we accepted all students who applied (Appendix B). Each of the three sessions in the second part lasted 1 hour and represented a deep dive into one or two of the core CEnR principles that had been discussed during the didactic session. Session 1 explored the principle of strong community–academic partnership (Appendix C), session 2 focused on equitable power and responsibility (Appendices D and E), and session 3 emphasized capacity building and effective dissemination (Appendix F). We divided the CEnR principles across the sessions in this way because they paralleled what our group felt was a natural progression of how a community–academic partnership might evolve; however, other groups wishing to implement this curriculum may choose to divide the CEnR principles across multiple sessions in a different way. We created a facilitator guide (Appendix G) to assist with replicating the sessions.

Each session was co-led by an academic partner and a community partner or medical learner with a history of collaboration with one another. We recommend the inclusion of community partners—ideally, a representative of a community-based organization with a personal and institutional history of significant partnership with the academic medical representative—to ensure that the sessions offer perspectives from both an academic and community partner. The medical learner, usually at a more advanced level than the participants, provided a near-peer perspective into how CEnR played out in their training, offering an accessible example to participants. In a large group, the partners shared their experiences and insights pertaining to the CEnR principles highlighted in the session and discussed a practical case study. The learner/community–academic partner presentation in the large group lasted approximately 20 minutes, with some session-to-session variation. After the presentation, learners broke into three small groups that ranged from eight to 10 students and represented a mixed sample of students from both institutions. The small groups lasted 30 minutes and were led by faculty members from the Office of Community Engagement. We recommend that the small-group discussions be led by a CEnR-experienced faculty, staff, senior learner, or community partner. During the small groups, students had the opportunity to ask questions and pursue discussion about the CEnR core principles for that session. We also provided the groups with principle-specific prompts to facilitate discussion.

There was a 1-week lapse between each session in the second part. We recommend that spacing between sessions be no more than 1 month to ensure a degree of the momentum that comes with learning and reflecting on new topics near one another. To accommodate the COVID-19 pandemic, we implemented the series virtually through Zoom. We screen-shared our PowerPoint slides with the learners and encouraged them to use Zoom's chat feature to ask questions throughout each session. The chat was monitored by a staff member in the Office of Community Engagement, and we paused at transition points during the session to respond to questions from the chat. We also used Zoom's breakout room function to conduct our small groups. Small groups were preassigned to automatically send learners to their assigned breakout room.

### Evaluation Plan

To measure the achievement of our objectives, we developed brief surveys that were administered through Qualtrics software at the conclusion of each of our four sessions (Appendix H for the initial didactic session; Appendix I for each subsequent small-group session). We were deliberate in keeping the surveys brief and simple to maximize our response rate. The surveys included both quantitative and qualitative (open-ended) questions to foster a varied understanding of the students' experiences in attending the sessions. We had pilot tested the surveys the prior year in the original iteration of the program, with small adjustments to ensure better clarity and more useful response data. The surveys focused on level 1 of Kirkpatrick's four levels of evaluation^23^ by assessing students' satisfaction/reaction regarding the content and their self-perception of learning.

## Results

Survey response rates varied between the large-group didactic session (49% of 160 participants) and small-group sessions 1 (67% of 36), 2 (42% of 31), and 3 (38% of 21; Table). Overall, 87% of respondents (96% small-group, 82% large-group) strongly or somewhat agreed that their session was worthwhile on a 5-point Likert scale (1 = *strongly disagree,* 5 = *strongly agree*). Specifically, 85% of large-group and 96% of small-group respondents felt they had learned something they would use in their practice/profession. Both groups found the speakers to be effective at communicating session content (98% strongly or somewhat agreed; Table). Respondents noted appreciation for the speakers' passion and sharing of stories and case studies, as well as for their sharing principles of community engagement that had not been previously encountered.

**Table:**
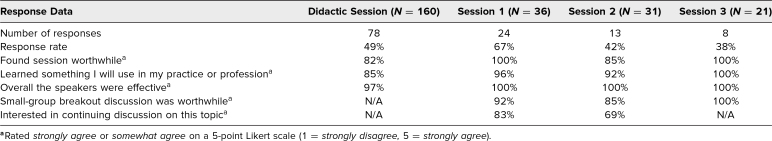
Survey Response Data Summarized Across Mandatory Didactic and Subsequent Optional Sessions

At the conclusion of the large-group, didactic session, nine of 78 respondents (12%) answered that yes, they planned to apply for the small-group series, and 30 (38%) answered maybe. Ultimately, 22 MCW students matriculated into the small-group series, where they were joined by 14 students from UNMC.

In addition, respondents from the large-group didactic noted appreciation for exposure to CEnR content (which they felt was lacking in other areas of the curriculum), appreciation for and challenges with efforts to engage/interact/build community remotely, intention to get involved in the community, and feelings of validation from students planning to embark on CEnR during the summer. One respondent wrote, “I found it very encouraging and inspirational to continue community-engaged research! They highlighted the importance of this type of research which I appreciated.”

Small-group session respondents suggested that more time in the speaker component and in interactive breakouts would have helped to engage them more thoroughly in CEnR topics. One respondent noted, “I wish we had more time in each, as I think we are limited on how much our presenters can teach us and how deep we can dive into discussion during small group by the current timeframe.” Suggestions included doubling the number of sessions or expanding each session by 30–60 minutes. There were also calls to focus on specific topics, such as building trust with communities of color that may have been mistreated by researchers in the past. Respondents noted appreciation for the community–academic dialogue format of sessions 1 and 2 and the student–faculty dialogue format of session 3. Additional feedback focused on adjusting the structure of small-group breakouts, including distributing questions ahead of time and including case studies for students to work through.

Between 69% and 83% of small-group respondents indicated interest in continuing their learning in the topic beyond the session. Interested participants connected with faculty after the summer or sought them out during later projects and experiences. Overall series feedback was positive, with the sense that this format was effective as a general introduction to CEnR. One respondent noted,

It was beneficial to be introduced to the topic and learn that there are many types of people working on this. It was helpful to have literature samples provided. It was good to get to ask questions…. I would have been willing to do multiple more sessions or 1.5-hour sessions.

## Discussion

The Foundations of Community Engagement series was effective at stimulating medical students' self-reported gains in CEnR knowledge, skills, and attitudes in an efficient manner. Students interacted with a variety of CEnR practitioners and applications. Participants reported a perception of knowledge gain, attitude shift, perspective expansion, and intention to further explore community context. Whether or not these participants make CEnR a substantial part of their future career, it is our hope that they will be more effective physicians who are more attuned to the context of the community and behavioral factors that influence their patients' health.

Development and implementation of the curriculum progressed in a stepwise manner and involved several community-engaged faculty members and long-standing community partners willing to substantially invest in sharing their experiences with medical students. Therefore, the resulting curriculum contained diverse perspectives and rich dialogue, which appeared to be a novel area of enrichment for medical students. The intricacy of the curriculum, intimacy of conversation, and personalization and use of individual partnerships and experiences as teaching tools required a high level of planning in advance of the session. Planning support from faculty or staff is recommended—initially to recruit instructors and community partners months in advance and then to plan specific dialogue and talking points closer to the dates of the session. We recommend that presenters (i.e., faculty, community partner or student, other coordinating team members) of each session meet beforehand to clarify plans for instruction, dialogue, and small-group facilitation. Although this series was implemented virtually, it seems likely that it would be just as successful (perhaps more) in an in-person setting. The diverse faculty and community experiences were a highlight of the student experience, and future educators would benefit from a similar approach. However, it is possible for fewer academic and community partners to feature in more of the sessions than are shown in the case study curriculum.

Although these sessions occurred in an academic context, we note that for those wishing to develop robust skills, CEnR must be learned in practice. We recommend offering learners emerging from this program opportunities to engage in true community work, ideally in partnership with a mentor skilled in CEnR. In the 2021 iteration of the program, a formal advising session was offered with the intention of connecting learners with individually tailored avenues for further CEnR enrichment. A prior iteration offered a follow-up journal club, and other connecting points could include a menu of active CEnR projects for interested students to consider joining or other locally available opportunities.

In addition, medical learners building capacity in CEnR principles are expected to be more effective at understanding community health needs and partnering with the community to meet those needs outside of the course of traditional medical care. Studying future application of CEnR principles is beyond the scope of this project, but the foundation provided by the program is expected to guide participants as they move along their path toward becoming the physicians of tomorrow.

This is a project of limited scope and without longitudinal follow-up. The curriculum emphasizes discussion between community–academic partners and medical students/teachers with experience doing CEnR work together, which may limit reproducibility. However, steps have been taken to guide such discussion to illuminate exploration of CEnR principles in different local contexts that can be used as case studies. Evaluation focused on general concepts and self-reported satisfaction and perception of learning, not content-specific questions or objective measurements of knowledge or behavior change. This curriculum, however, is one of the few examples of CEnR education for medical students in such a format, which we believe to be an accessible and effective approach to teaching CEnR concepts and stimulating future physicians to simultaneously learn the community context of their patients alongside the clinical knowledge and skills they are also developing. Future work devising and studying effective CEnR curricula for this population and measuring longitudinal impact is needed.

## Appendices


CE Didactic Session Slides.pptxApplication for Small-Group Series.docxCommunity-Academic Partnership Slides.pptxEquitable Power and Responsibility Slides.pptxEquitable Power and Responsibility Case Studies.docxCapacity Building and Dissemination Slides.pptxFacilitator Guide.docxCE Didactic Session Evaluation.docxSmall-Group Session Evaluation.docx
*All appendices are peer reviewed as integral parts of the Original Publication.*
