# Supplementary material for: Foundations of Community Engagement: A Series for Effective Community-Engaged Research
Source: MedEdPORTAL. 2023 Oct 10;19:11350. doi: 10.15766/mep_2374-8265.11350 (PMC10562524; doi:10.15766/mep_2374-8265.11350)
Supplement: Supplementary file 1 — CE Didactic Session Slides.pptxApplication for Small-Group Series.docxCommunity-Academic Partnership Slides.pptxEquitable Power and Responsibility Slides.pptxEquitable Power and Responsibility Case Studies.docxCapacity Building and Dissemination Slides.pptxFacilitator Guide.docxCE Didactic Session Evaluation.docxSmall-Group Session Evaluation.docx [file mep_2374-8265.11350-s001.zip › B. Application for Small-Group Series.docx]

**Application for Virtual Community Engagement Student Summer Series**

- Applications are due [INSERT DATE, TIME HERE]
- Prior community engagement experience is not required
- Offered by the [INSERT OFFICE/DEPT & INSTITUTION HERE]
- Questions? Contact [INSERT CONTACT NAME & CONTACT INFO HERE]

To apply to participate in this cohort, please complete the questions below.

1. **Your name**
2. **Your email address**
3. **What institution/campus are you from?**
   - [INSERT NAME OF YOUR INSTITUTION HERE]
   - Other
4. **What type of student/learner are you?**

- Medical
  - What year are you in your medical school training?
    - M1
    - M2
    - M3
    - M4
- Graduate
- What degree program are you in?
- Pharmacy
- Resident
- Other
- What type of student/learner are you?

1. **Are you participating in the summer research program?** [IF APPLICABLE TO YOUR INSTITUTION]
2. **If you have a summer advisor, is that person aware that you are applying for the Virtual Community Engagement Student Summer Series?** [IF APPLICABLE TO YOUR INSTITUTION]
3. **Please indicate that you can participate in all 3 of the sessions listed below by selecting all 3 dates. If you are not available for all sessions, please indicate the sessions for which you are available.**

- [INSERT SESSION 1 DATE & TIME HERE]
- [INSERT SESSION 2 DATE & TIME HERE]
- [INSERT SESSION 3 DATE & TIME HERE]

1. **Tell us about your interest in community engagement.** *Please limit your answer to one paragraph.*
2. **Do your educational plans involve community engagement? If so, how? Examples of your educational plans could include a summer research program, scholarly pathway, field placement, etc.** *Please note: this is not a requirement for acceptance to the program.*

Thank you for your application.

You will be notified by email as to the status of your acceptance into the Virtual Community Engagement Student Summer Series by [INSERT DATE HERE].

Questions? Please contact [INSERT CONTACT NAME & CONTACT INFO HERE].
