# Supplementary material for: Foundations of Community Engagement: A Series for Effective Community-Engaged Research
Source: MedEdPORTAL. 2023 Oct 10;19:11350. doi: 10.15766/mep_2374-8265.11350 (PMC10562524; doi:10.15766/mep_2374-8265.11350)
Supplement: Supplementary file 1 — CE Didactic Session Slides.pptxApplication for Small-Group Series.docxCommunity-Academic Partnership Slides.pptxEquitable Power and Responsibility Slides.pptxEquitable Power and Responsibility Case Studies.docxCapacity Building and Dissemination Slides.pptxFacilitator Guide.docxCE Didactic Session Evaluation.docxSmall-Group Session Evaluation.docx [file mep_2374-8265.11350-s001.zip › D. Equitable Power and Responsibility Slides.pptx]

## Slide 1
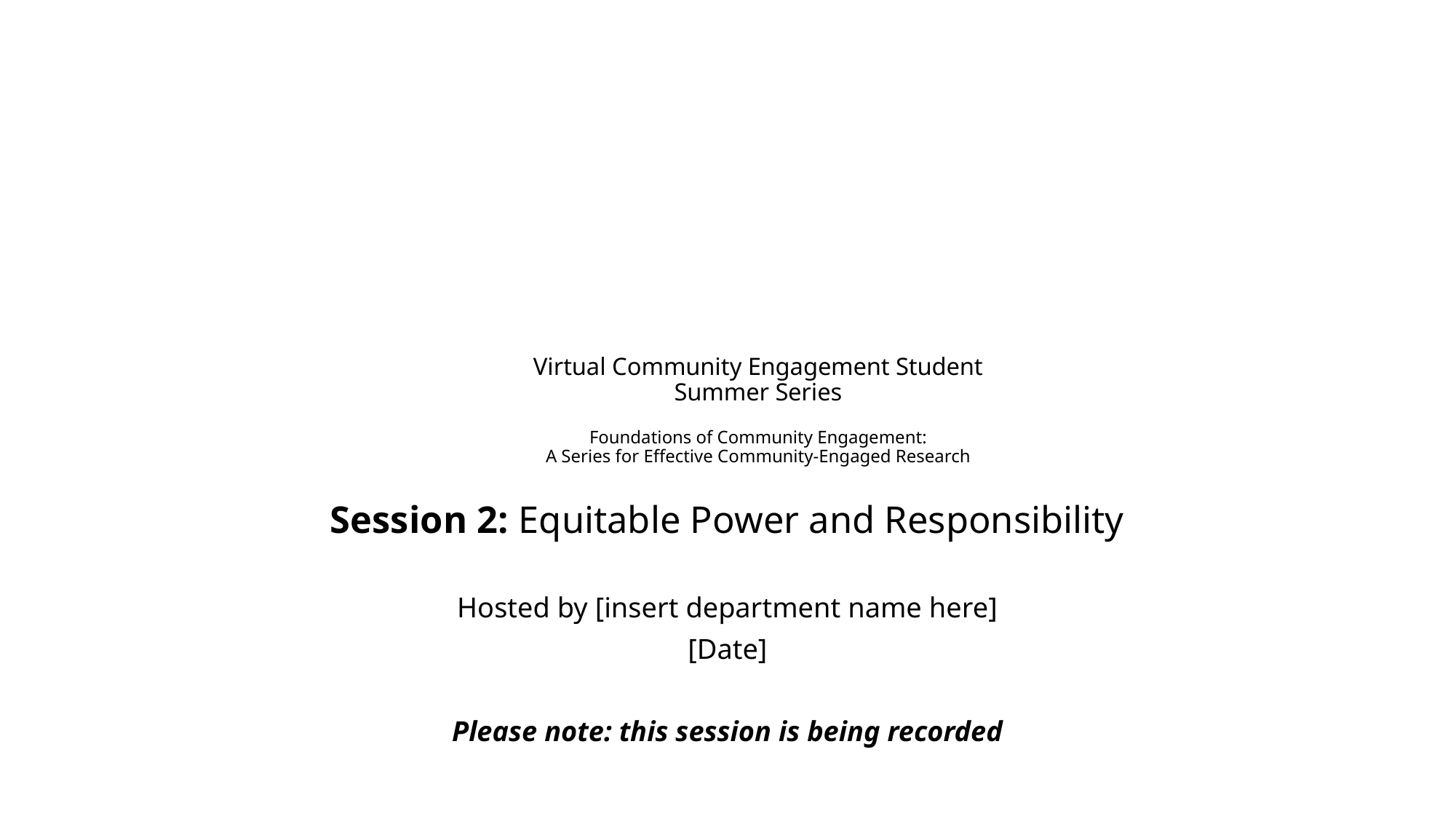

# Virtual Community Engagement Student Summer Series Foundations of Community Engagement: A Series for Effective Community-Engaged Research
Session 2: Equitable Power and Responsibility
Hosted by [insert department name here]
[Date]
Please note: this session is being recorded

## Slide 2
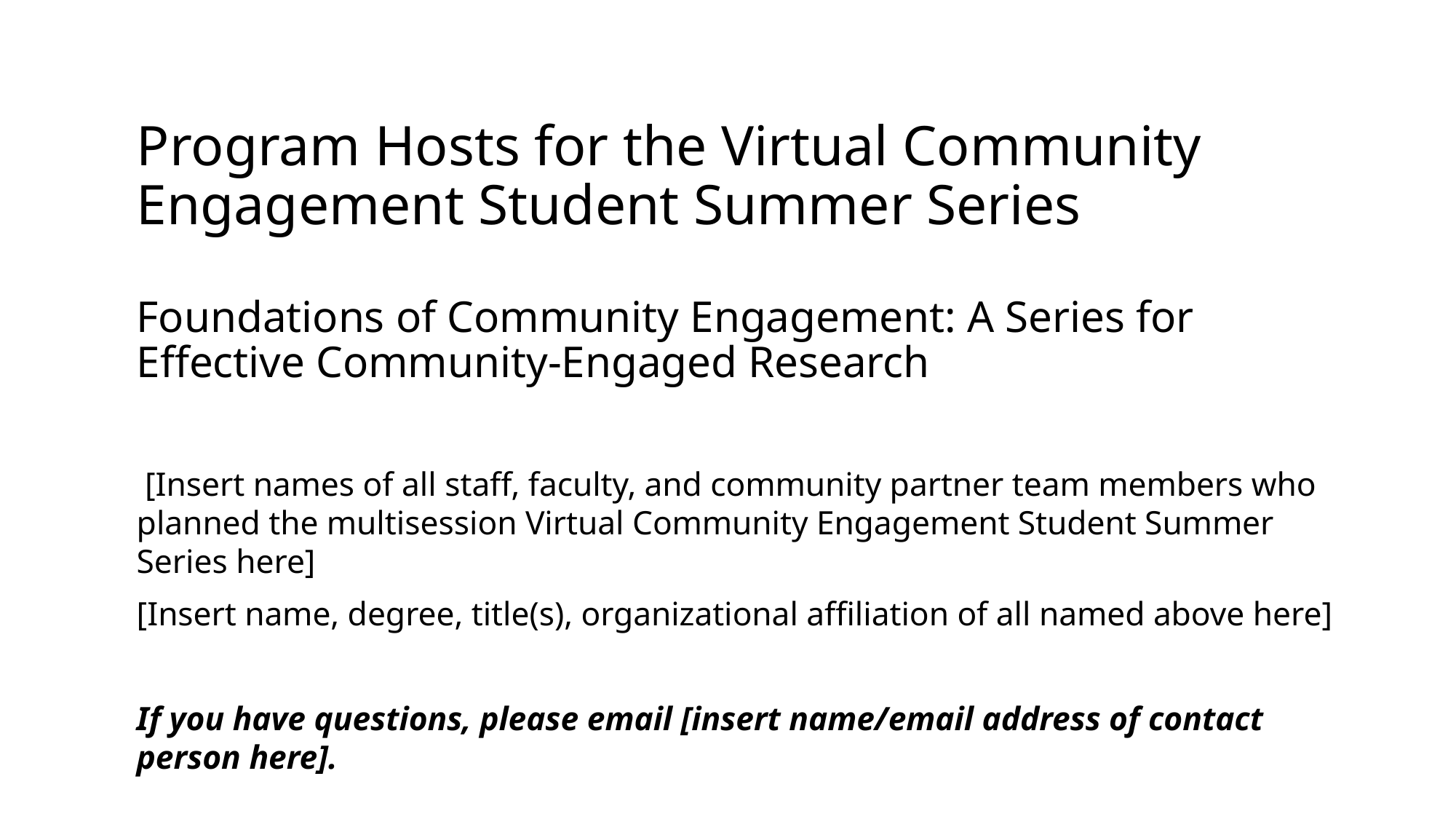

# Program Hosts for the Virtual Community Engagement Student Summer Series Foundations of Community Engagement: A Series for Effective Community-Engaged Research
 [Insert names of all staff, faculty, and community partner team members who planned the multisession Virtual Community Engagement Student Summer Series here]
[Insert name, degree, title(s), organizational affiliation of all named above here]
If you have questions, please email [insert name/email address of contact person here].

## Slide 3
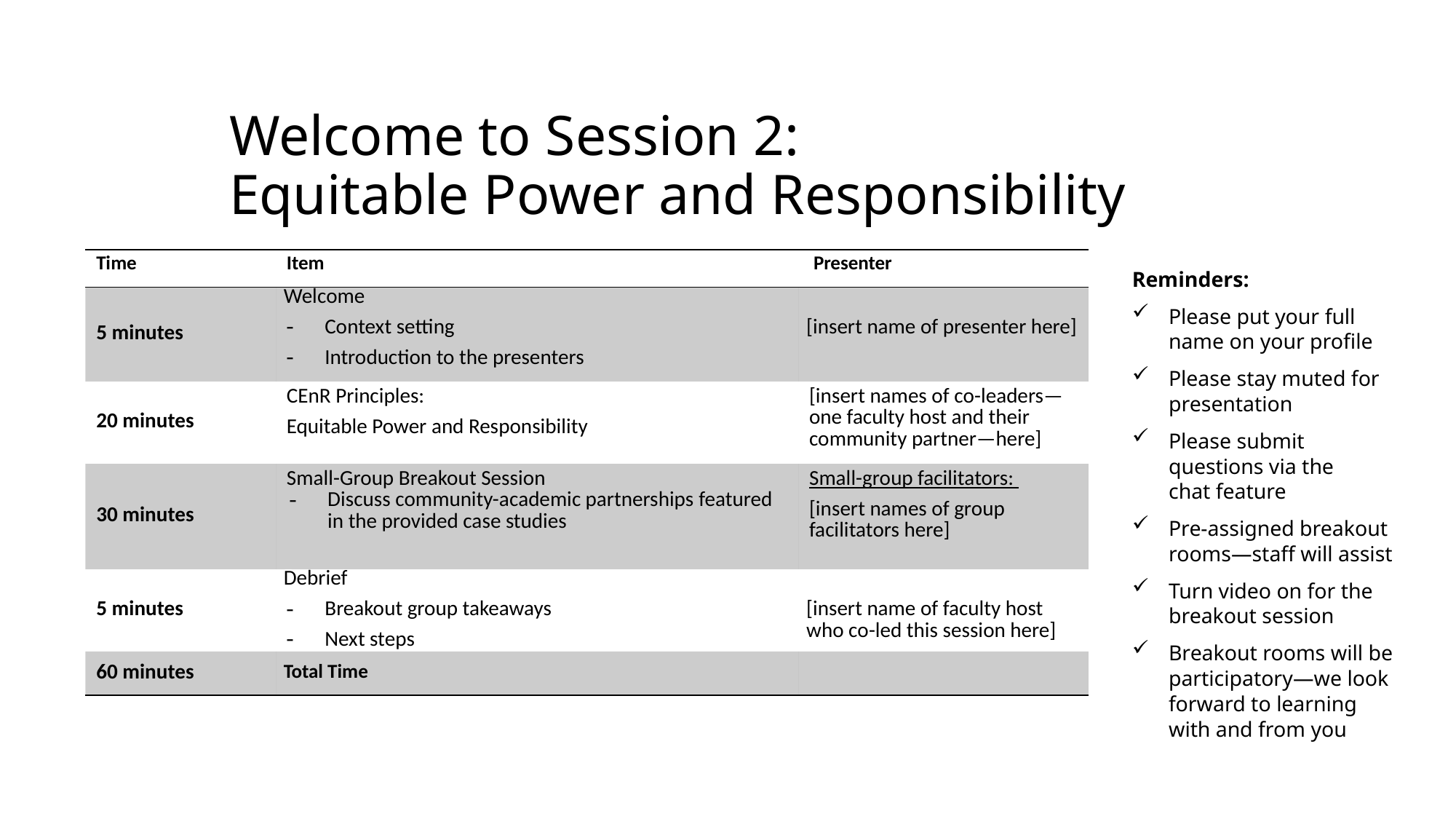

# Welcome to Session 2: Equitable Power and Responsibility
| Time​ | Item​ | ​Presenter |
| --- | --- | --- |
| 5 minutes | Welcome Context setting Introduction to the presenters | [insert name of presenter here] |
| 20 minutes | CEnR Principles:​ Equitable Power and Responsibility | [insert names of co-leaders—one faculty host and their community partner—here] |
| 30 minutes | Small-Group Breakout Session​ Discuss community-academic partnerships featured in the provided case studies | Small-group facilitators: [insert names of group facilitators here]​ |
| 5 minutes | Debrief Breakout group takeaways Next steps | [insert name of faculty host who co-led this session here] |
| 60 minutes | Total Time | |
Reminders:
Please put your full name on your profile
Please stay muted for presentation
Please submit questions via the chat feature
Pre-assigned breakout rooms—staff will assist
Turn video on for the breakout session
Breakout rooms will be participatory—we look forward to learning with and from you

## Slide 4
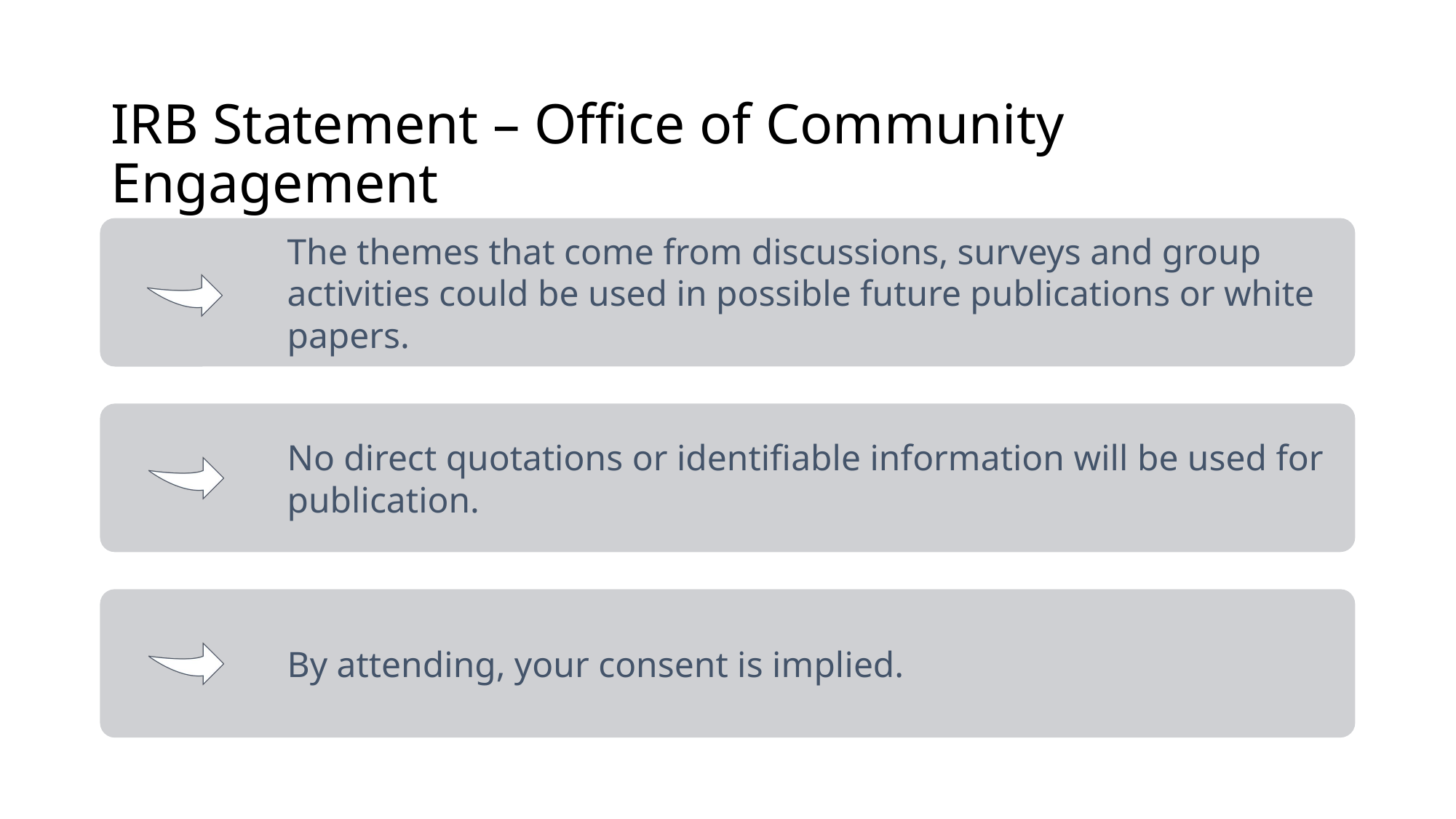

# IRB Statement – Office of Community Engagement

## Slide 5
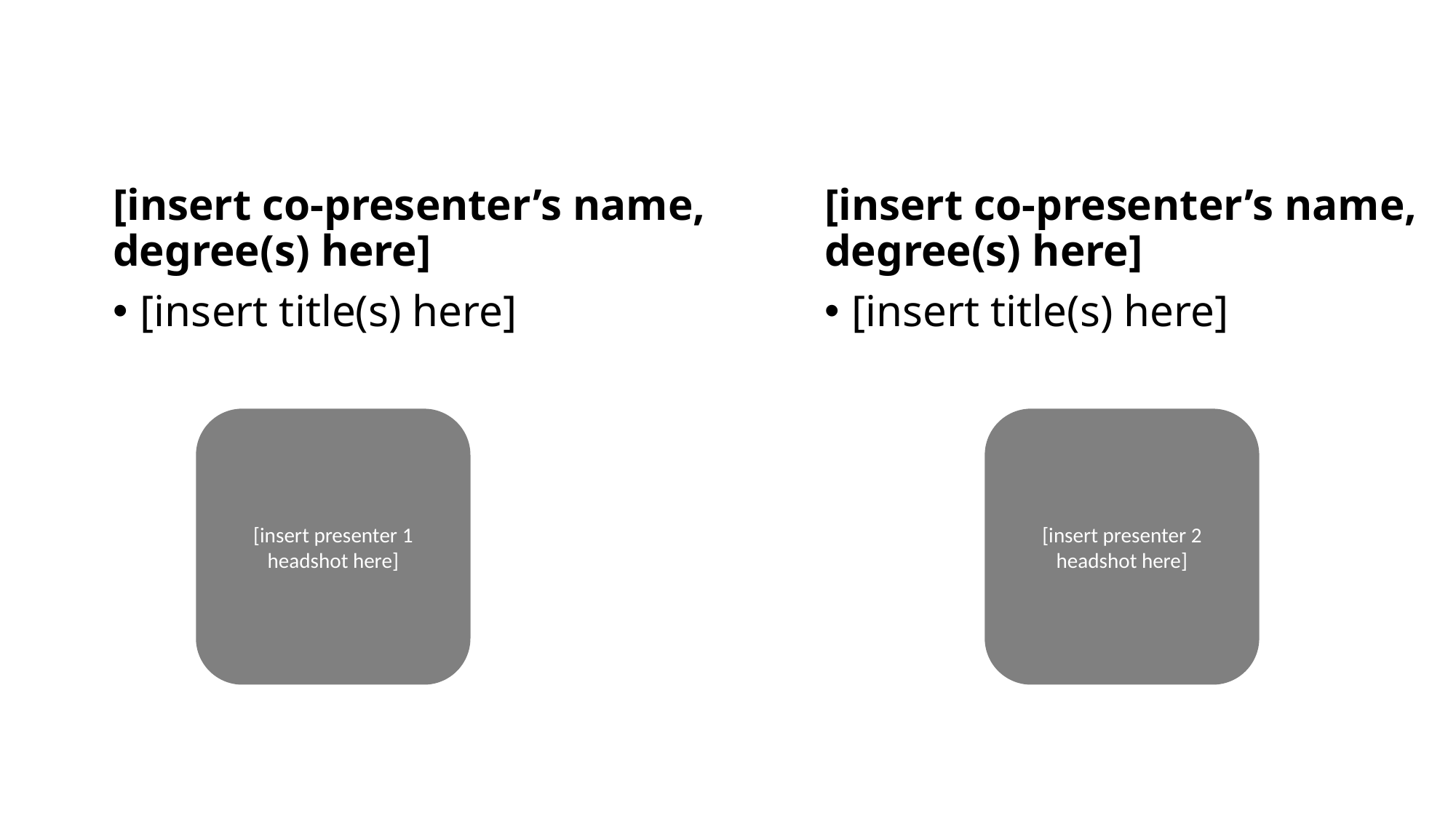

[insert co-presenter’s name, degree(s) here]
[insert title(s) here]
[insert co-presenter’s name, degree(s) here]
[insert title(s) here]
[insert presenter 1 headshot here]
[insert presenter 2 headshot here]

## Slide 6
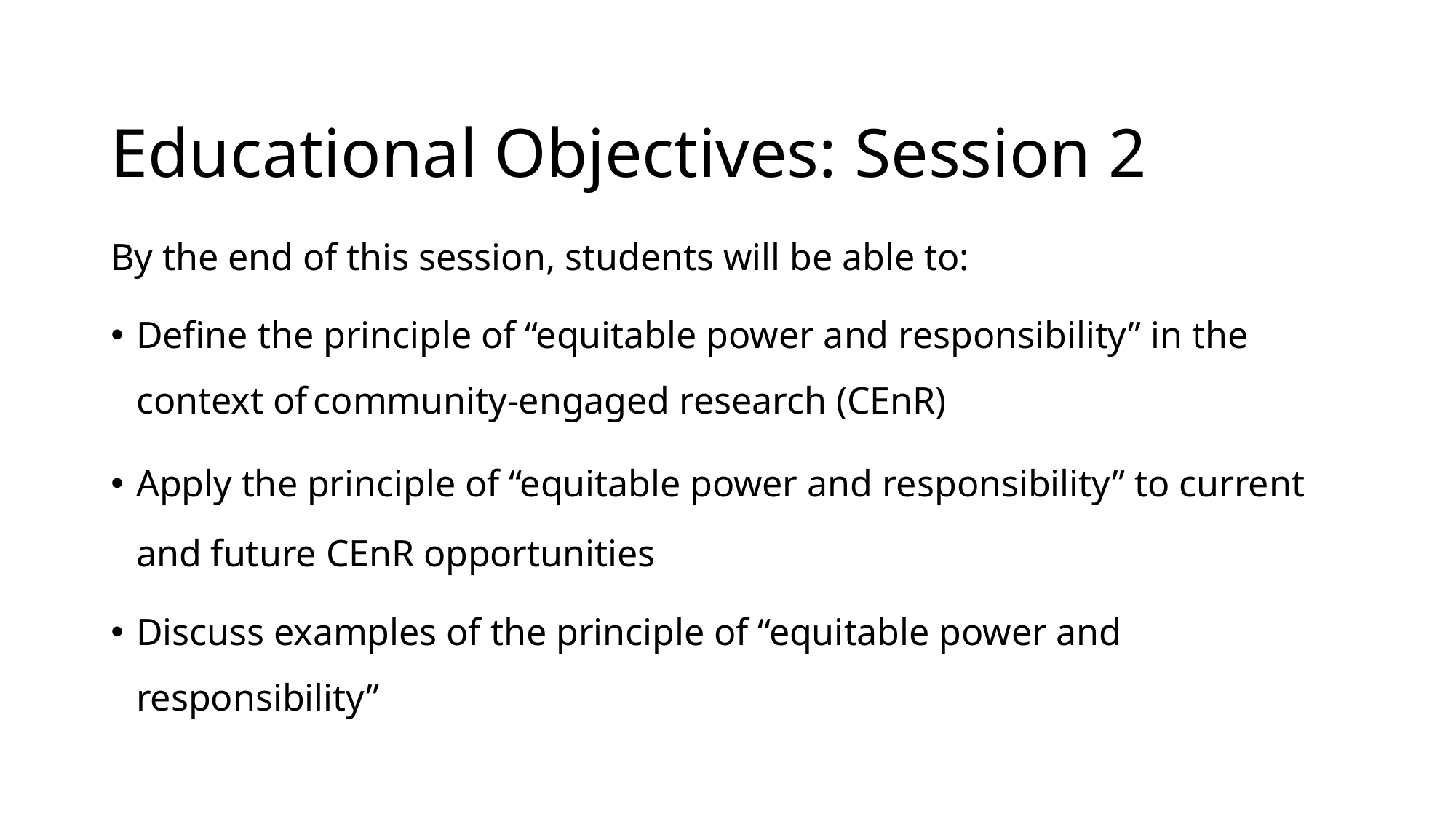

# Educational Objectives: Session 2
By the end of this session, students will be able to:
Define the principle of “equitable power and responsibility” in the context of community-engaged research (CEnR)
Apply the principle of “equitable power and responsibility” to current and future CEnR opportunities
Discuss examples of the principle of “equitable power and responsibility”

## Slide 7
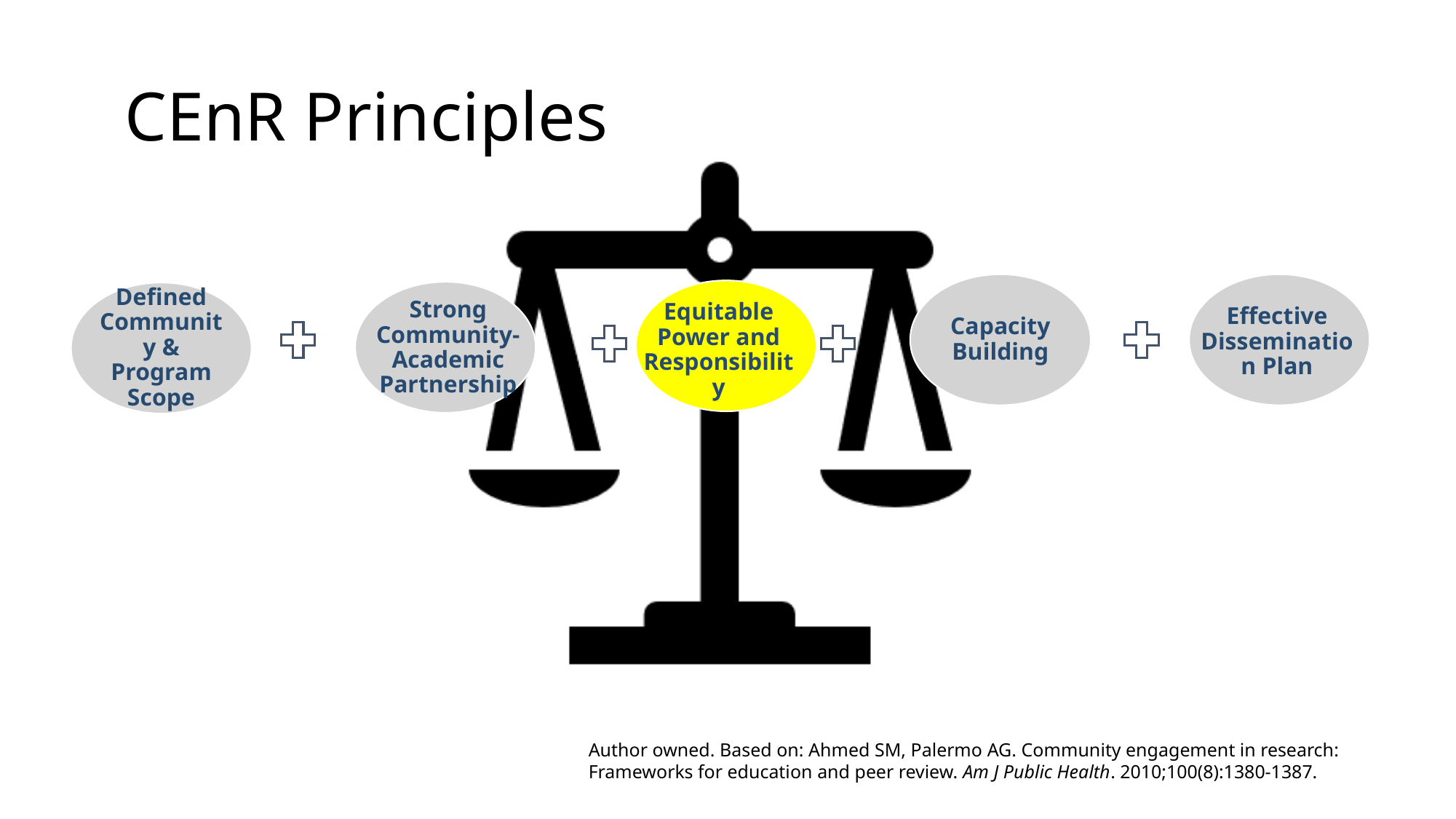

# CEnR Principles
Capacity Building
Effective Dissemination Plan
Equitable Power and Responsibility
Strong Community-Academic Partnership
Defined Community & Program Scope
Author owned. Based on: Ahmed SM, Palermo AG. Community engagement in research: Frameworks for education and peer review. Am J Public Health. 2010;100(8):1380-1387.

## Slide 8
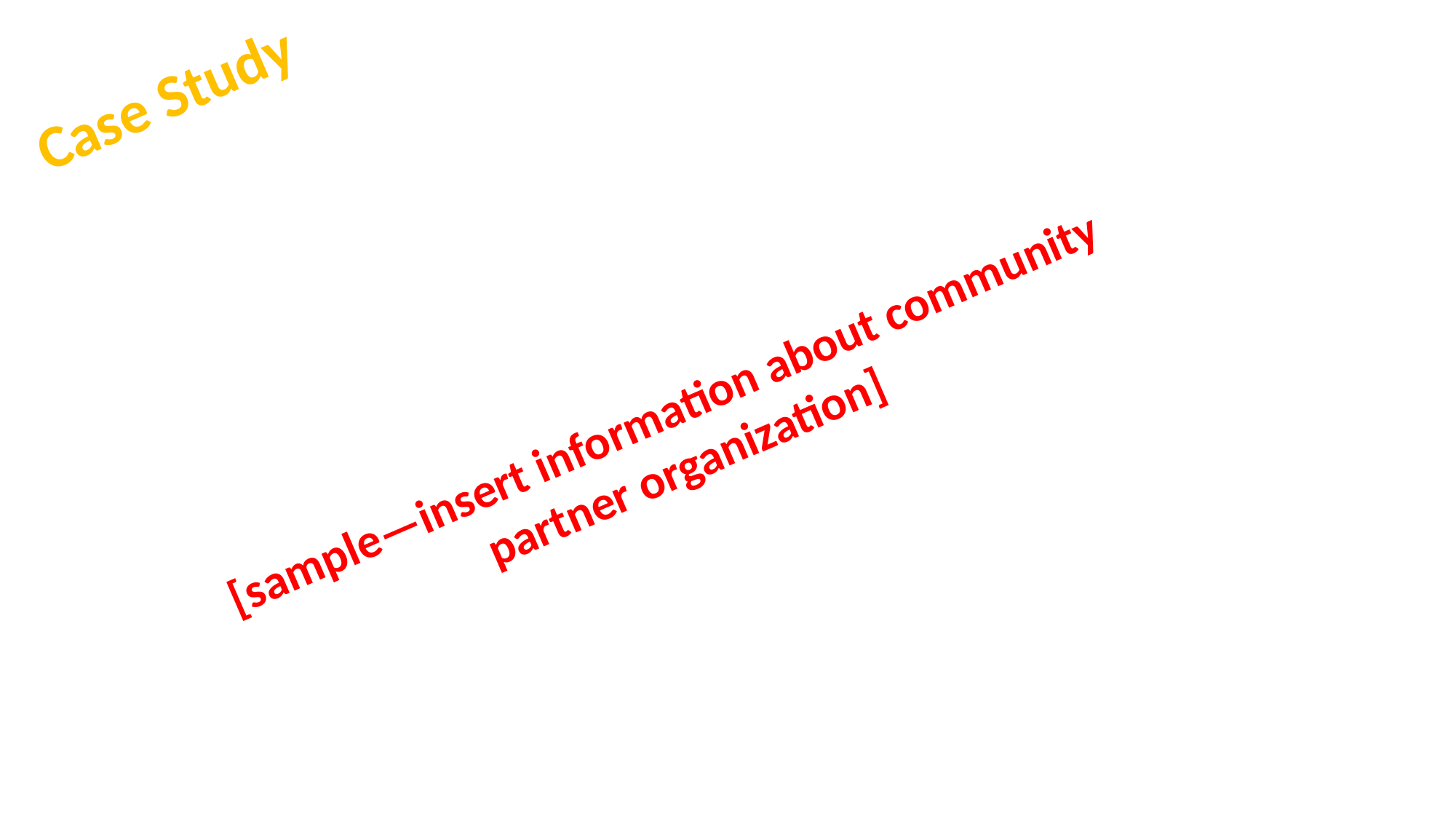

#
Case Study
[sample—insert information about community partner organization]

## Slide 9
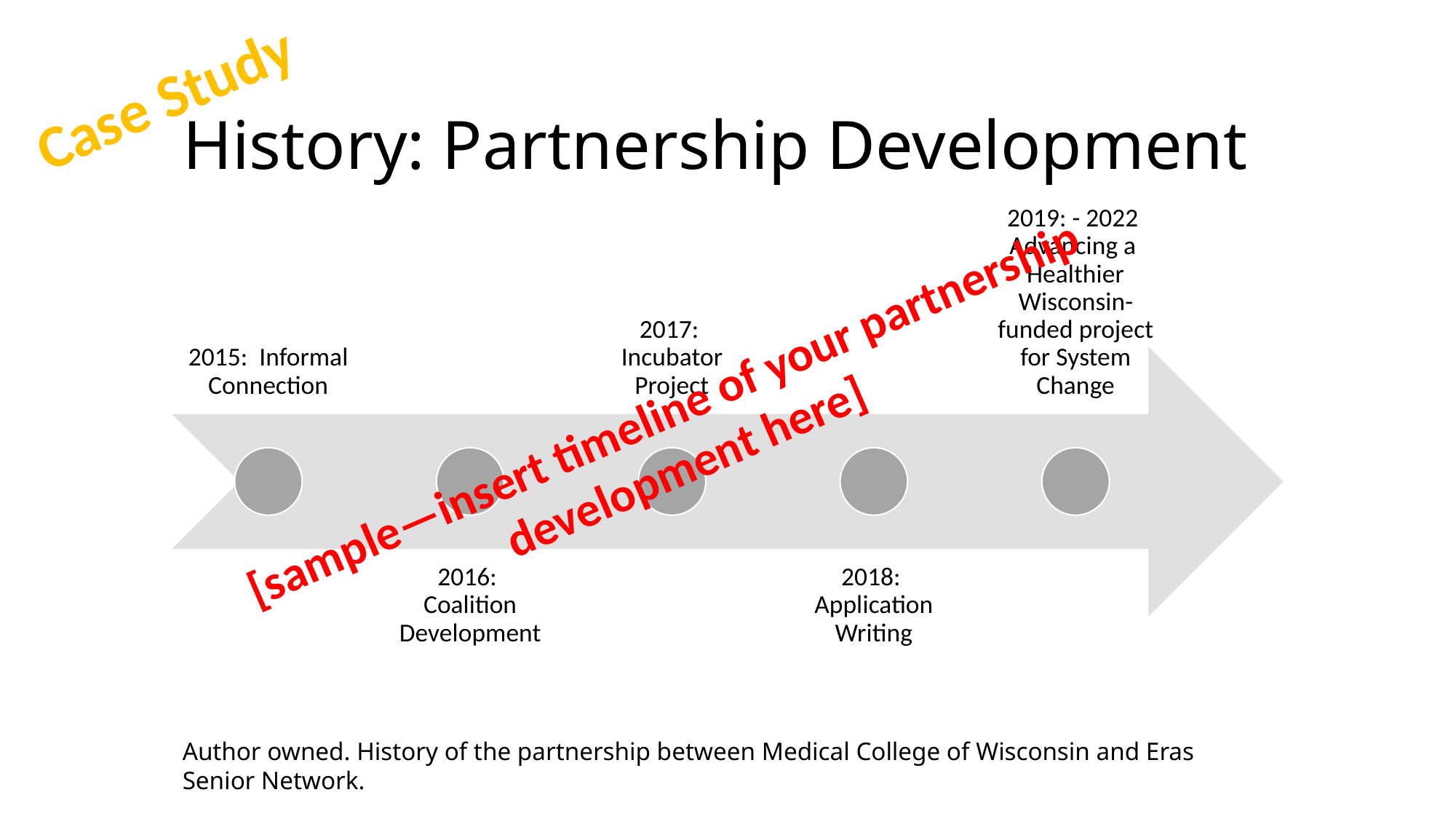

Case Study
# History: Partnership Development
[sample—insert timeline of your partnership development here]
Author owned. History of the partnership between Medical College of Wisconsin and Eras Senior Network.

## Slide 10
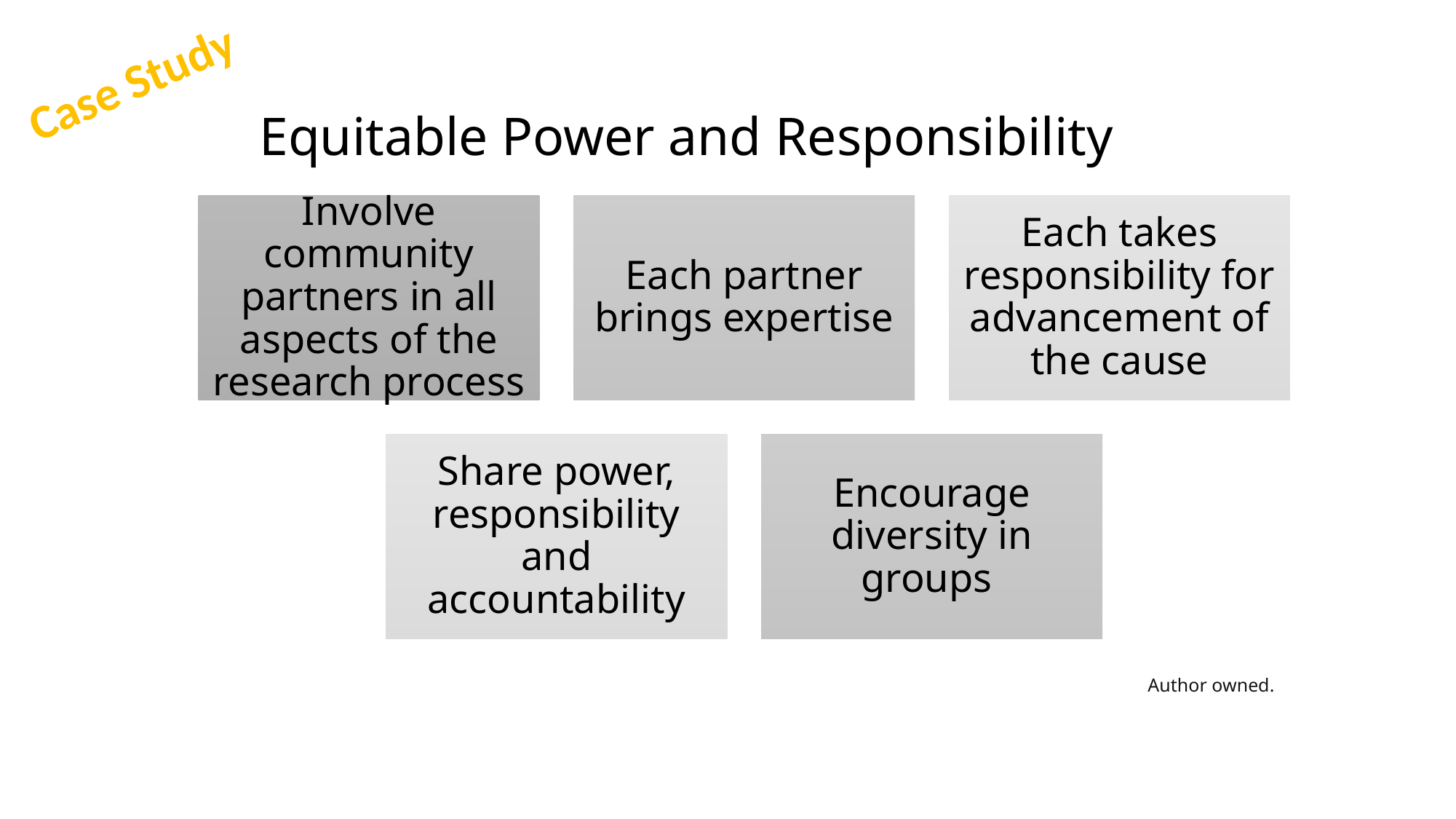

Case Study
# Equitable Power and Responsibility
Author owned.

## Slide 11
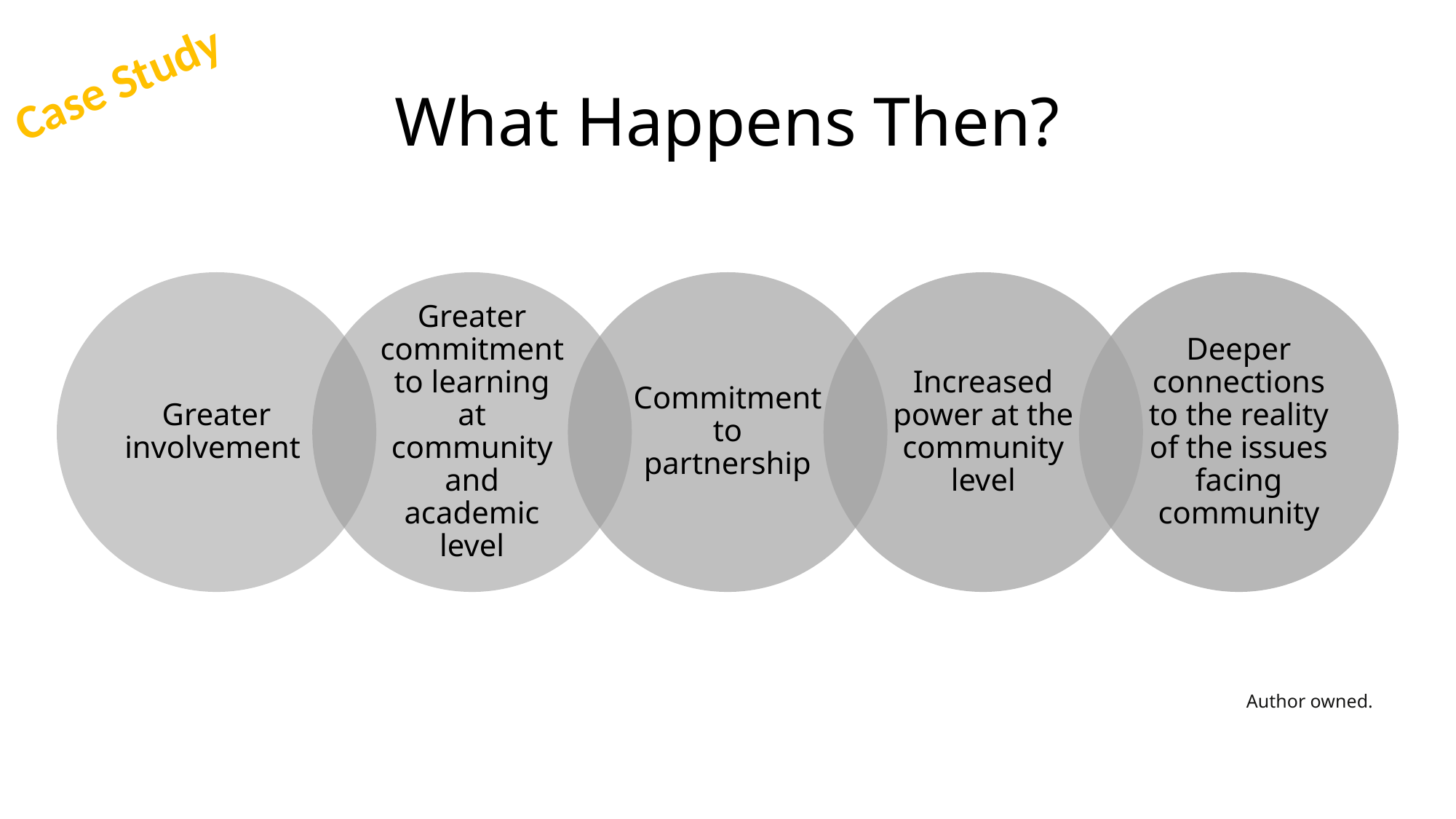

# What Happens Then?
Case Study
Author owned.

## Slide 12
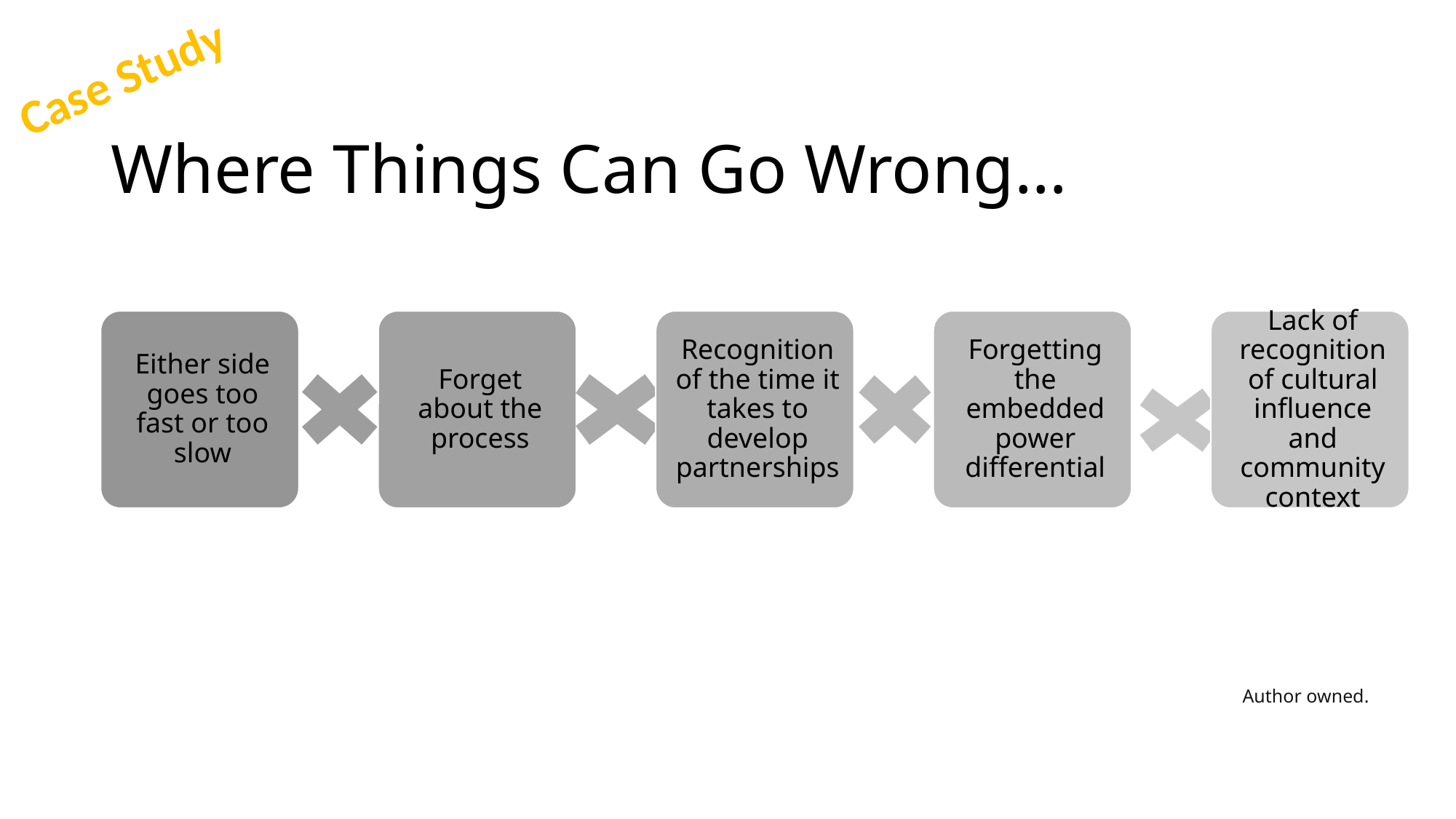

Case Study
# Where Things Can Go Wrong…
Author owned.

## Slide 13
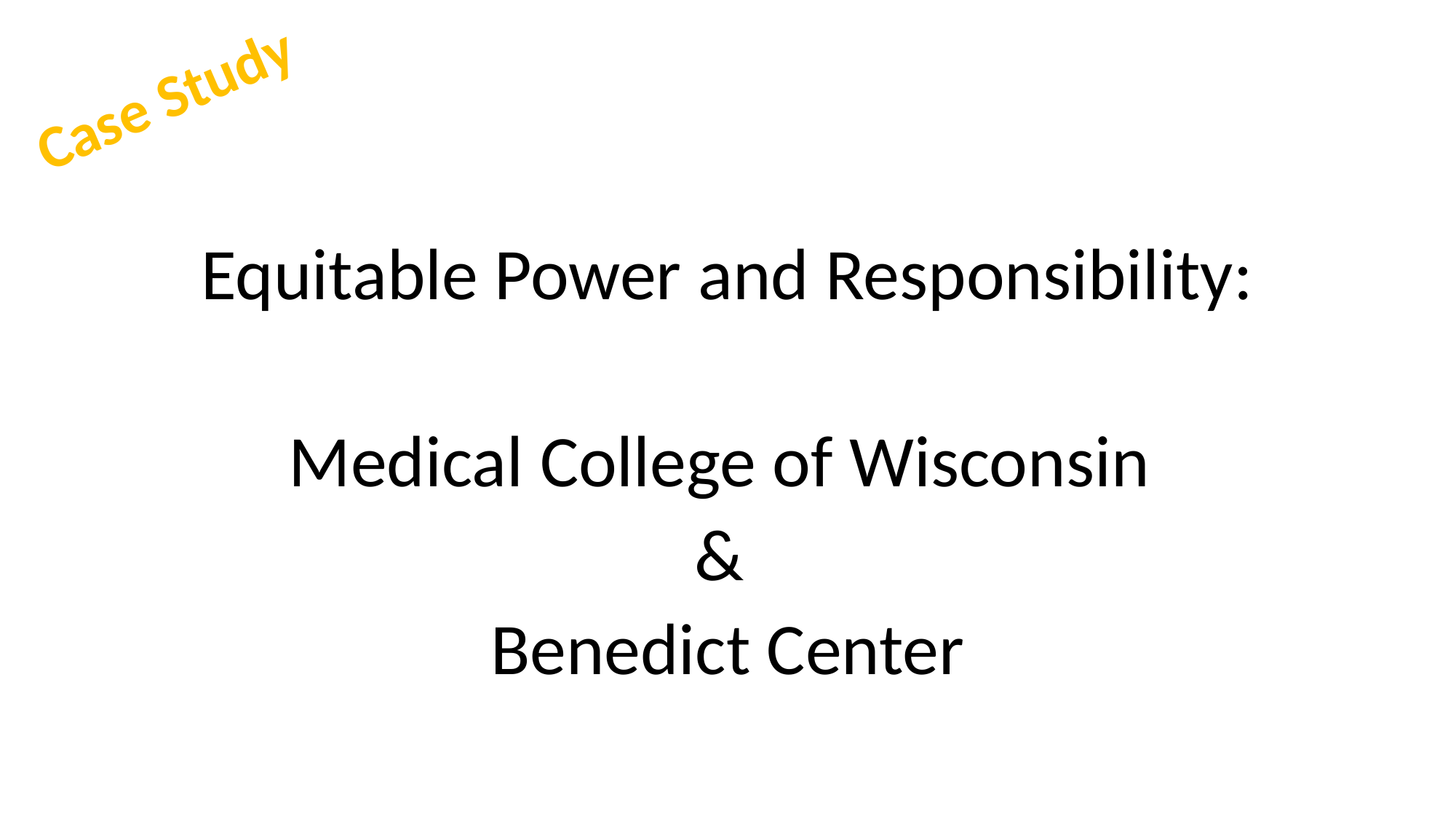

Case Study
Equitable Power and Responsibility:
Medical College of Wisconsin
&
Benedict Center

## Slide 14
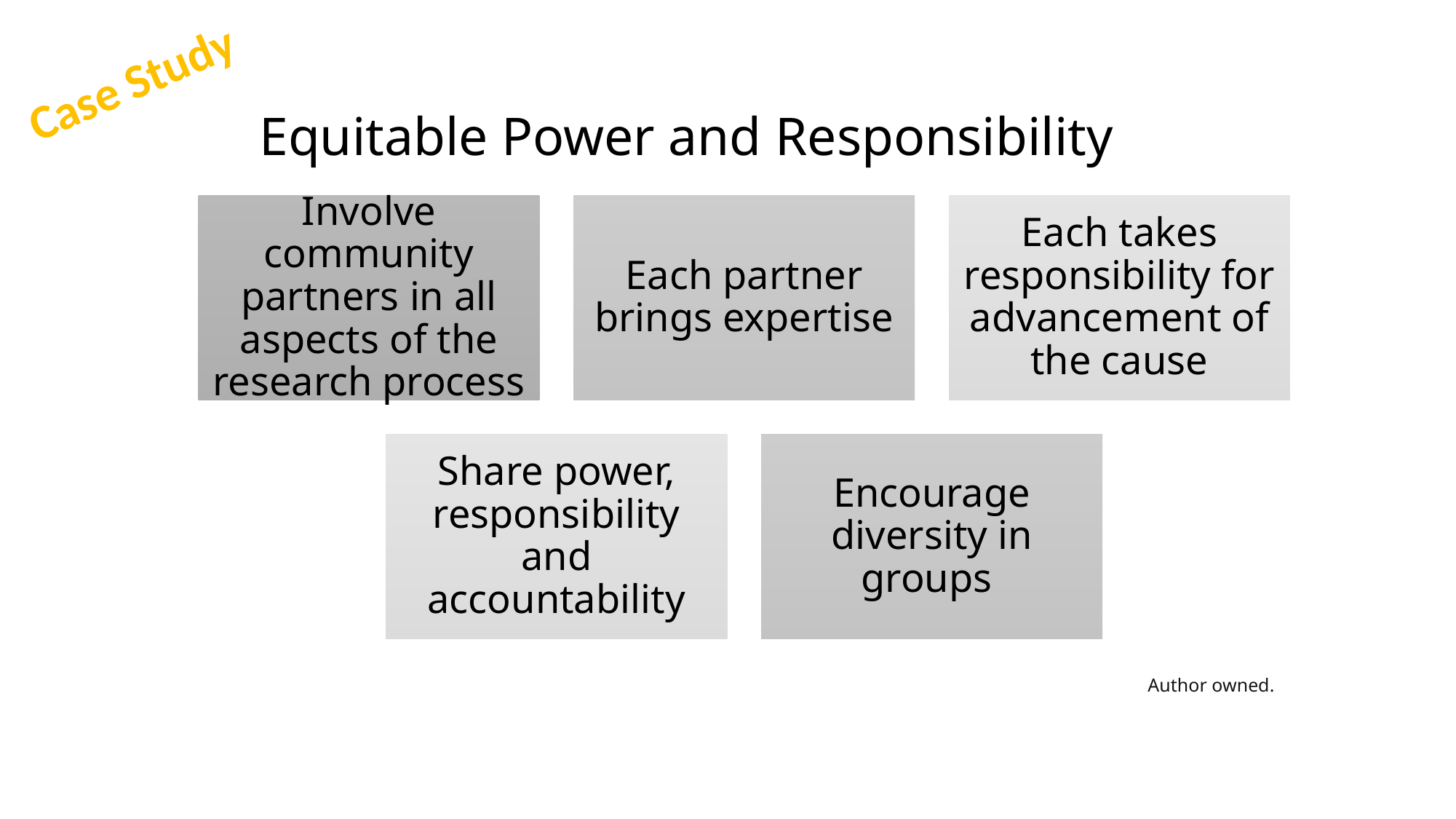

Case Study
# Equitable Power and Responsibility
Author owned.

## Slide 15
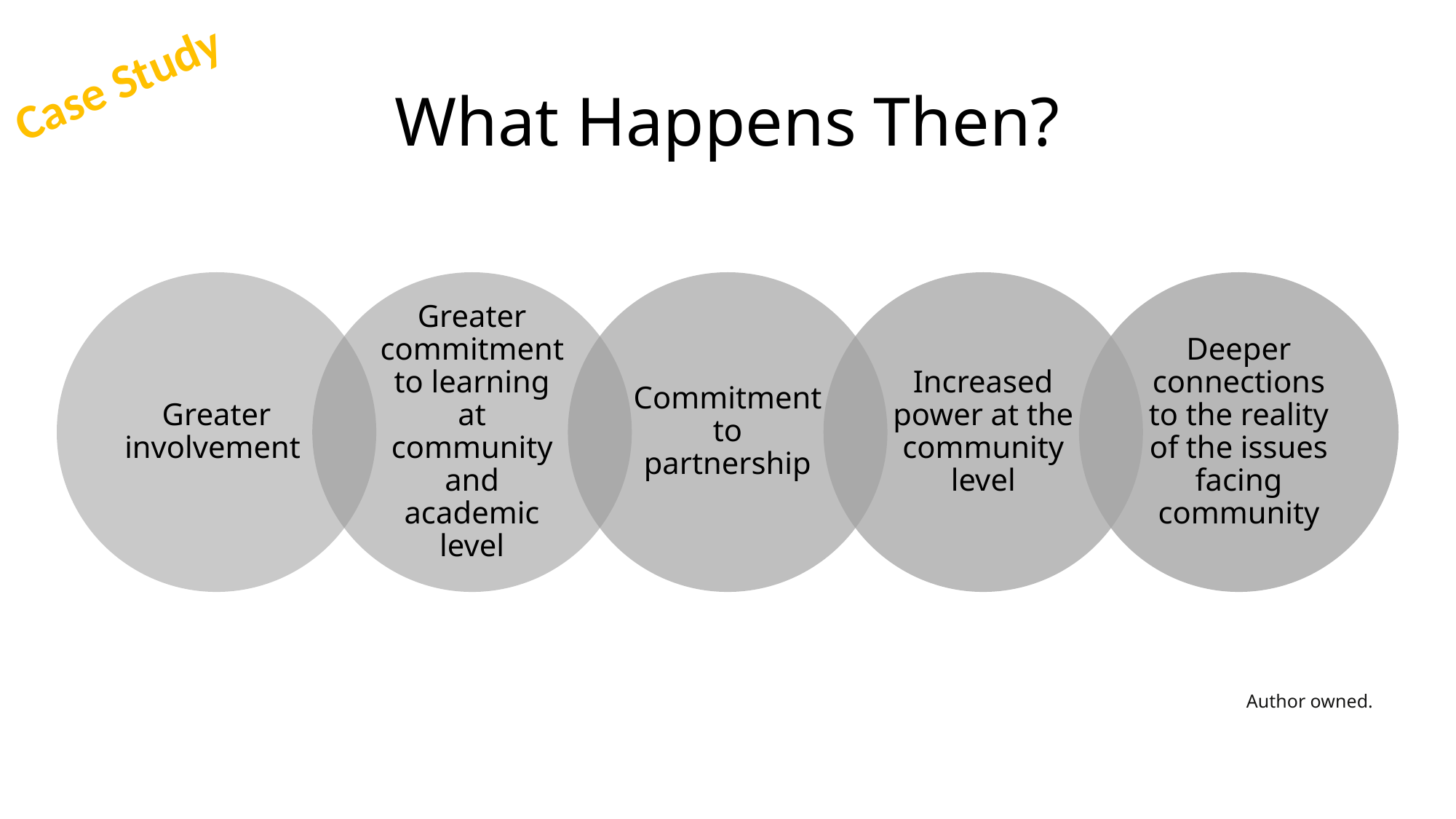

# What Happens Then?
Case Study
Author owned.

## Slide 16
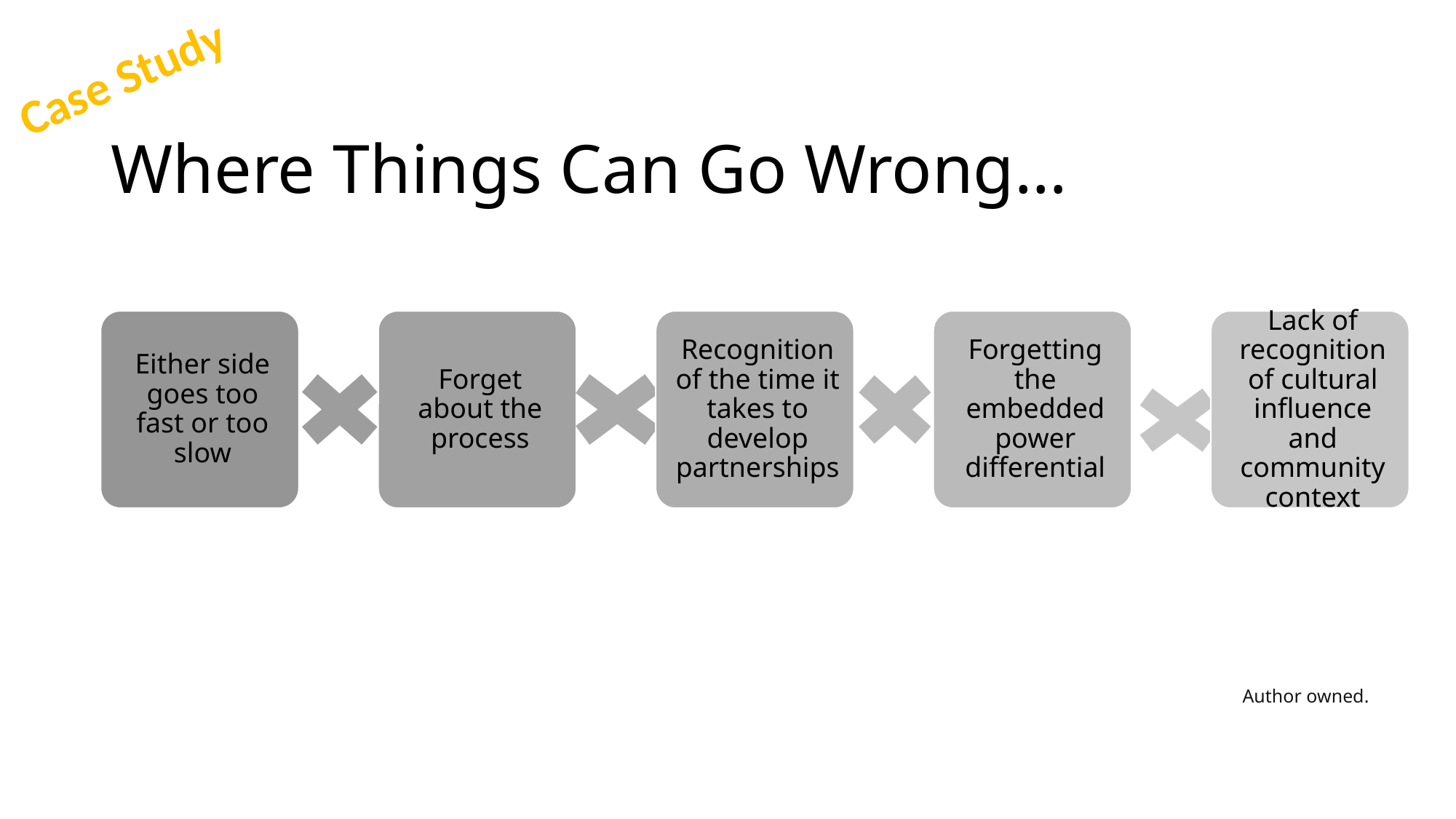

Case Study
# Where Things Can Go Wrong…
Author owned.

## Slide 17
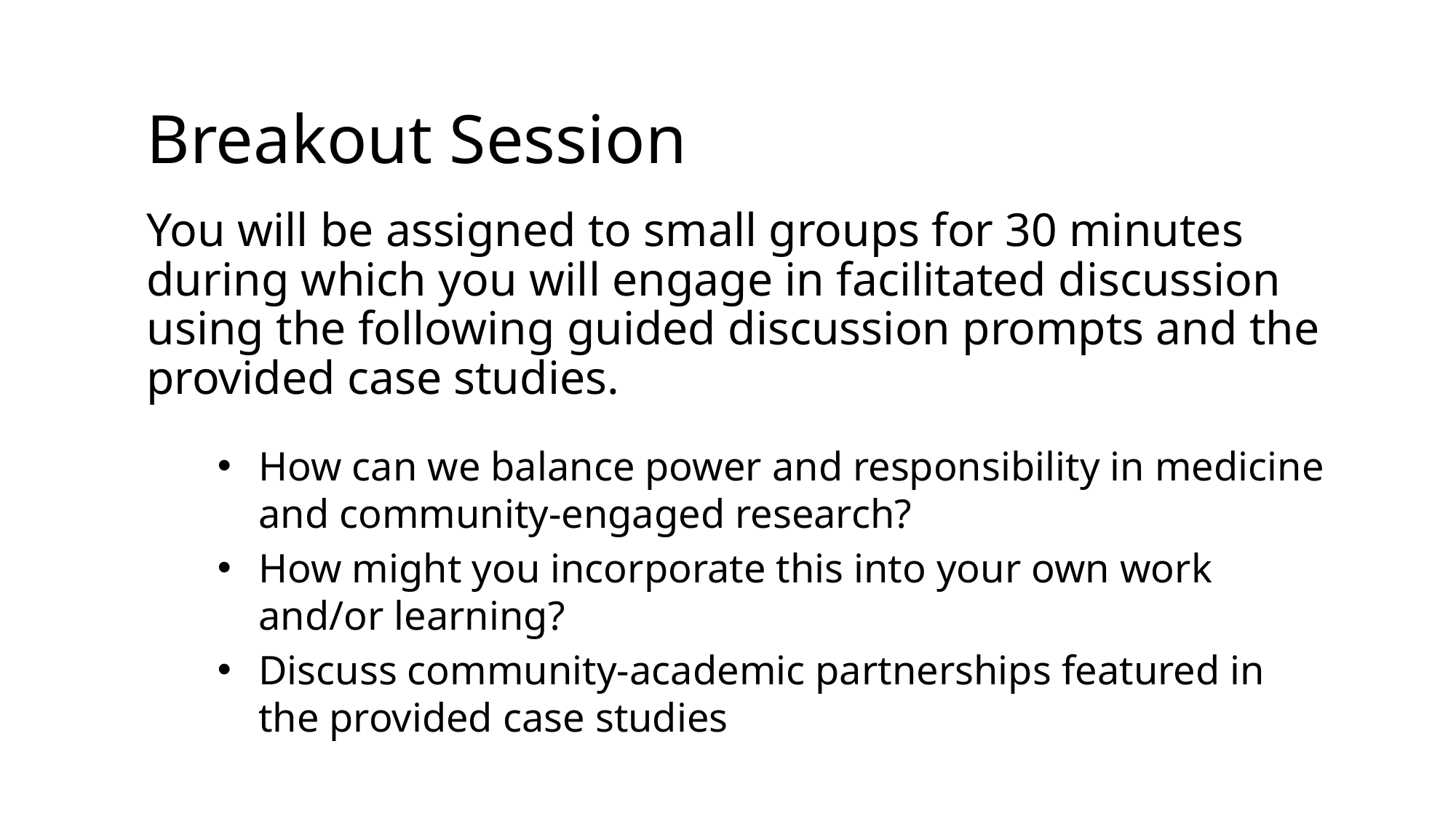

# Breakout Session
You will be assigned to small groups for 30 minutes during which you will engage in facilitated discussion using the following guided discussion prompts and the provided case studies.
How can we balance power and responsibility in medicine and community-engaged research?
How might you incorporate this into your own work and/or learning?
Discuss community-academic partnerships featured in the provided case studies

## Slide 18
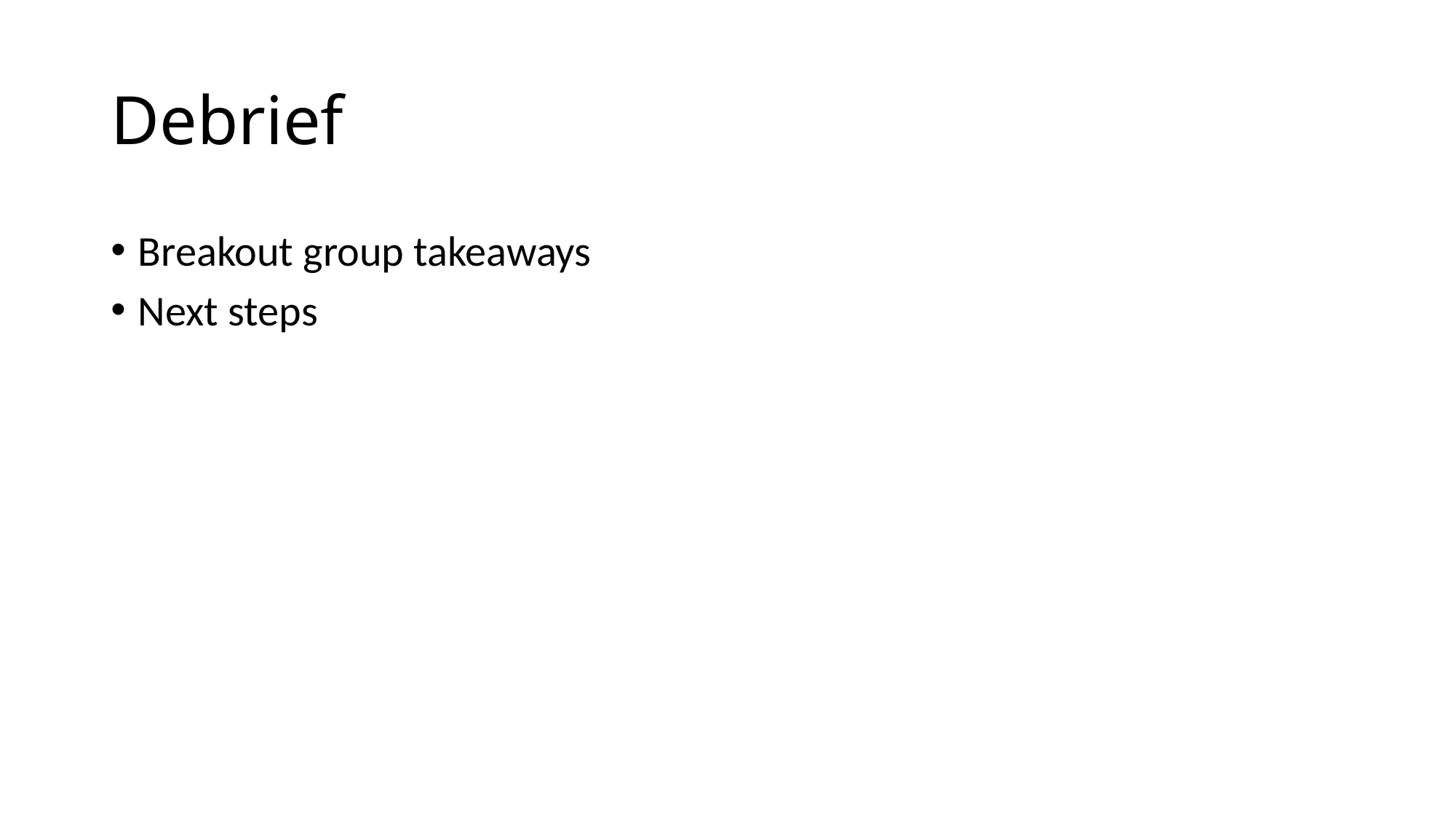

# Debrief
Breakout group takeaways
Next steps

## Slide 19
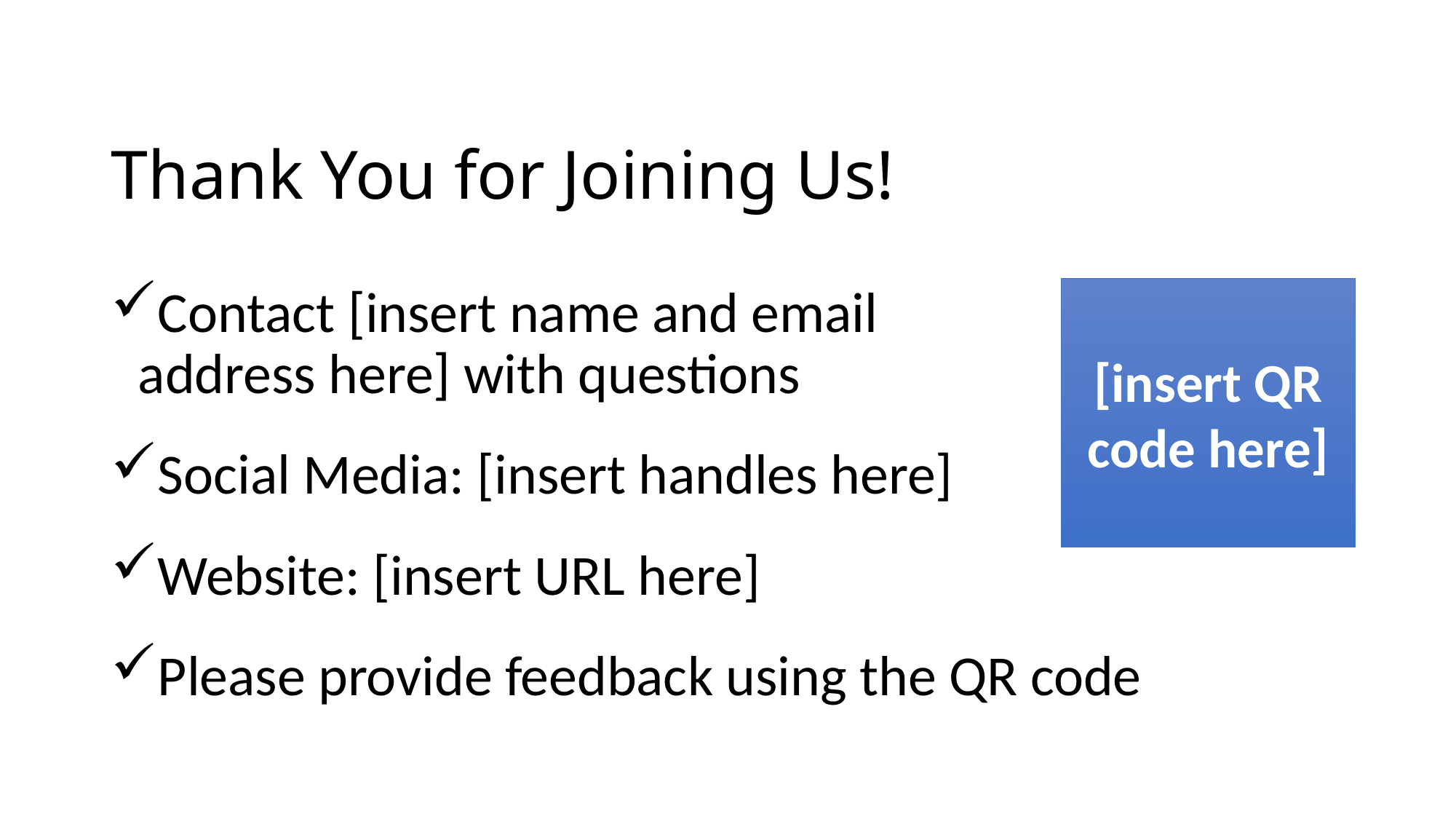

# Thank You for Joining Us!
Contact [insert name and email
address here] with questions
Social Media: [insert handles here]
Website: [insert URL here]
Please provide feedback using the QR code
[insert QR code here]

## Slide 20
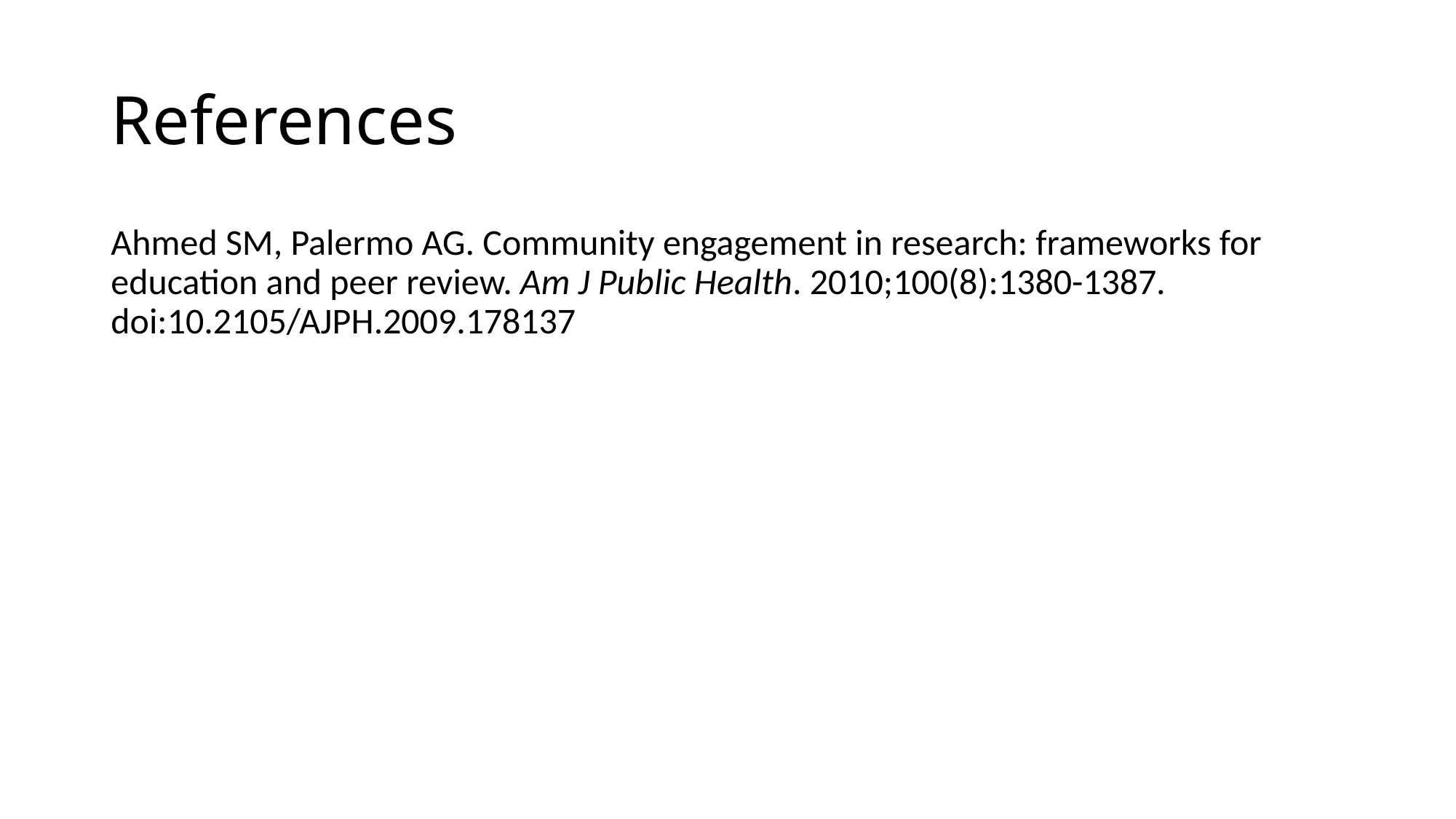

# References
Ahmed SM, Palermo AG. Community engagement in research: frameworks for education and peer review. Am J Public Health. 2010;100(8):1380-1387. doi:10.2105/AJPH.2009.178137
