# Supplementary material for: Foundations of Community Engagement: A Series for Effective Community-Engaged Research
Source: MedEdPORTAL. 2023 Oct 10;19:11350. doi: 10.15766/mep_2374-8265.11350 (PMC10562524; doi:10.15766/mep_2374-8265.11350)
Supplement: Supplementary file 1 — CE Didactic Session Slides.pptxApplication for Small-Group Series.docxCommunity-Academic Partnership Slides.pptxEquitable Power and Responsibility Slides.pptxEquitable Power and Responsibility Case Studies.docxCapacity Building and Dissemination Slides.pptxFacilitator Guide.docxCE Didactic Session Evaluation.docxSmall-Group Session Evaluation.docx [file mep_2374-8265.11350-s001.zip › E. Equitable Power and Responsibility Case Studies.docx]

Case Study #1: Funding Fumble

For the last 6 years a community academic partnership operated a drug abuse intervention program in Memphis, Tennessee. The funding for the program is due to expire at the end of the year, and the partnership has not been successful in finding other funding opportunities to sustain the program. Because they are the only substance abuse program in the area, a loss of funding could be devastating to the community.

As both partners research other funding opportunities, Dusk 2 Dawn learns about a new opportunity and informs Dr. O.P., their academic partner. Dr. O.P. explains to the staff from Dusk 2 Dawn that this particular funding opportunity does not really match the program’s goals and interests, and that it would be most beneficial to continue looking for other sources of funding, rather than apply for it.

The staff members of Dusk 2 Dawn are furious at Dr. O.P.’s response and feel that because Dr. O.P. is from academia she thinks that she should dictate how everything operates.

Discussion Questions

1. What are the main issues here?
2. Does the fact that Dr. O.P. is from academia give her insight that the community may not have about the funding process?
3. Keeping in mind the importance of sustainability and capacity building, what can Dr. O.P. do to let go of some of the control to let Dusk 2 Dawn take a more active role in pursuing funding?

Case Study #2: Misunderstanding the Rules of Engagement

In the late 1980s, a number of steel mills and other factories closed in Gary, Indiana, leaving a large population of unemployed and uninsured families. For the last 20 years, a local community health center and the emergency department of the city’s main hospital have been treating the uninsured for chronic medical problems. The hospital and the community center are now both understaffed and do not have the resources to continue providing these services.

Moira Low, the director of the center, contacted Dr. B.A., a family physician of the state’s medical college, about developing a city-wide health care program for the uninsured. In the past, she has read about the successful program Dr. B.A. and a community clinic ran for 15 years in Philadelphia and thought she would be a good resource. Because neither the center nor the hospital had any previous experience with program development or grant writing, they would need Dr. B.A.’s help to jumpstart the program. As expected, Dr. B.A. was very knowledgeable and offered a wealth of information. The two set up a time to meet at the center with the entire group and agreed to bring a list of ideas to discuss.

When the day of the meeting arrived, because Dr. B.A. had committed herself to too many projects she was not able to go to the meeting. She sent Dr. M., a well-respected academician, in her place to meet with the group and discuss ideas. The same day, Moira Low left a message on Dr. B.A.’s answering machine explaining how upset she was that Dr. M. came in her place. She also described Dr. M. as very hard to understand and very uncooperative, and she stated that she was not sure if her contacting Dr. B.A. had been such a good idea.

Discussion Questions

1. Besides rescheduling the meeting, what could Dr. B.A. have done to avoid angering Moira Low?
2. What can be done to rectify this situation?
3. What topics might have been addressed during initial communications to help avoid the misunderstanding?
