# Supplementary material for: Foundations of Community Engagement: A Series for Effective Community-Engaged Research
Source: MedEdPORTAL. 2023 Oct 10;19:11350. doi: 10.15766/mep_2374-8265.11350 (PMC10562524; doi:10.15766/mep_2374-8265.11350)
Supplement: Supplementary file 1 — CE Didactic Session Slides.pptxApplication for Small-Group Series.docxCommunity-Academic Partnership Slides.pptxEquitable Power and Responsibility Slides.pptxEquitable Power and Responsibility Case Studies.docxCapacity Building and Dissemination Slides.pptxFacilitator Guide.docxCE Didactic Session Evaluation.docxSmall-Group Session Evaluation.docx [file mep_2374-8265.11350-s001.zip › F. Capacity Building and Dissemination Slides.pptx]

## Slide 1
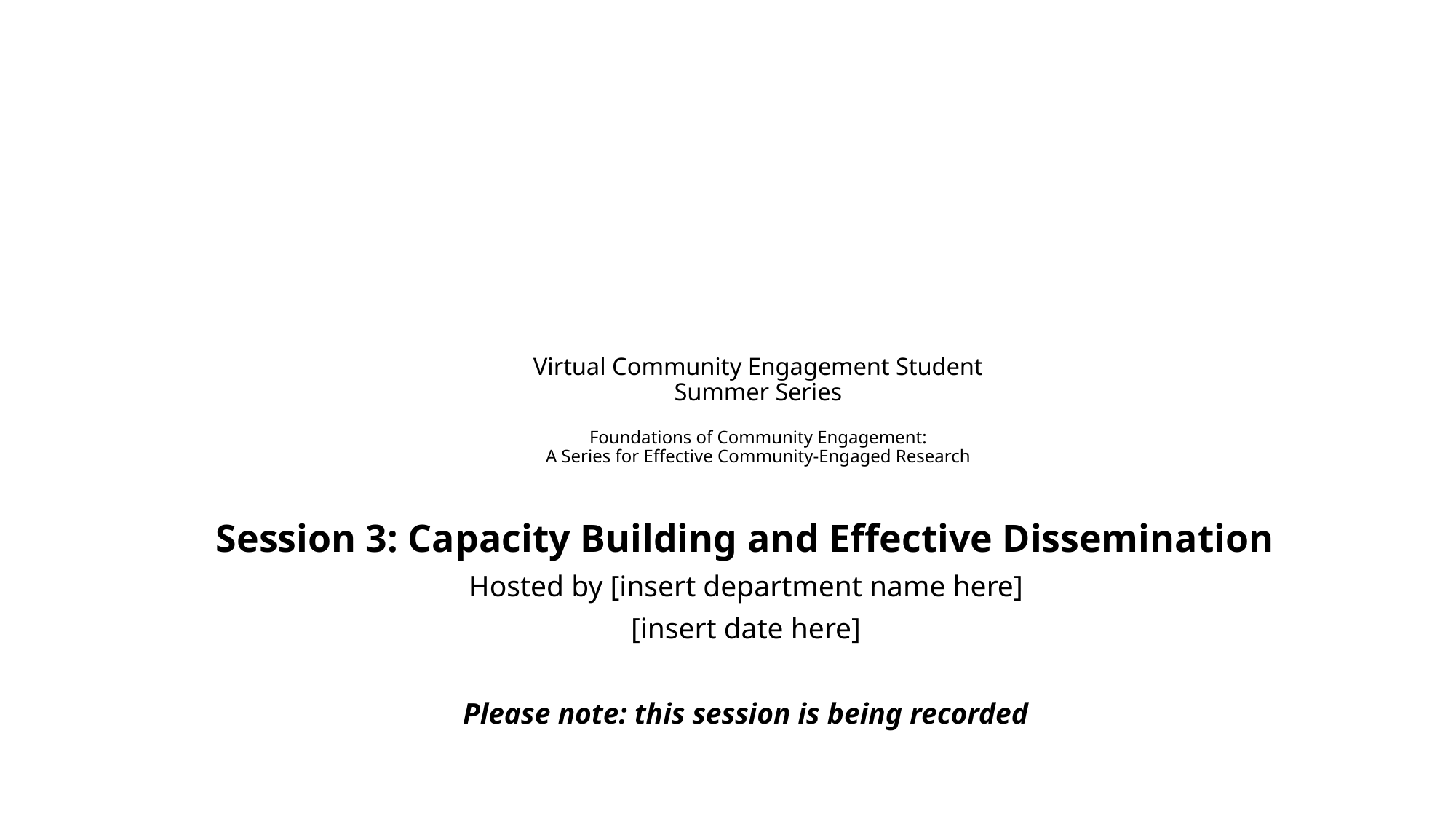

# Virtual Community Engagement Student Summer Series Foundations of Community Engagement: A Series for Effective Community-Engaged Research
Session 3: Capacity Building and Effective Dissemination
Hosted by [insert department name here]
[insert date here]
Please note: this session is being recorded

## Slide 2
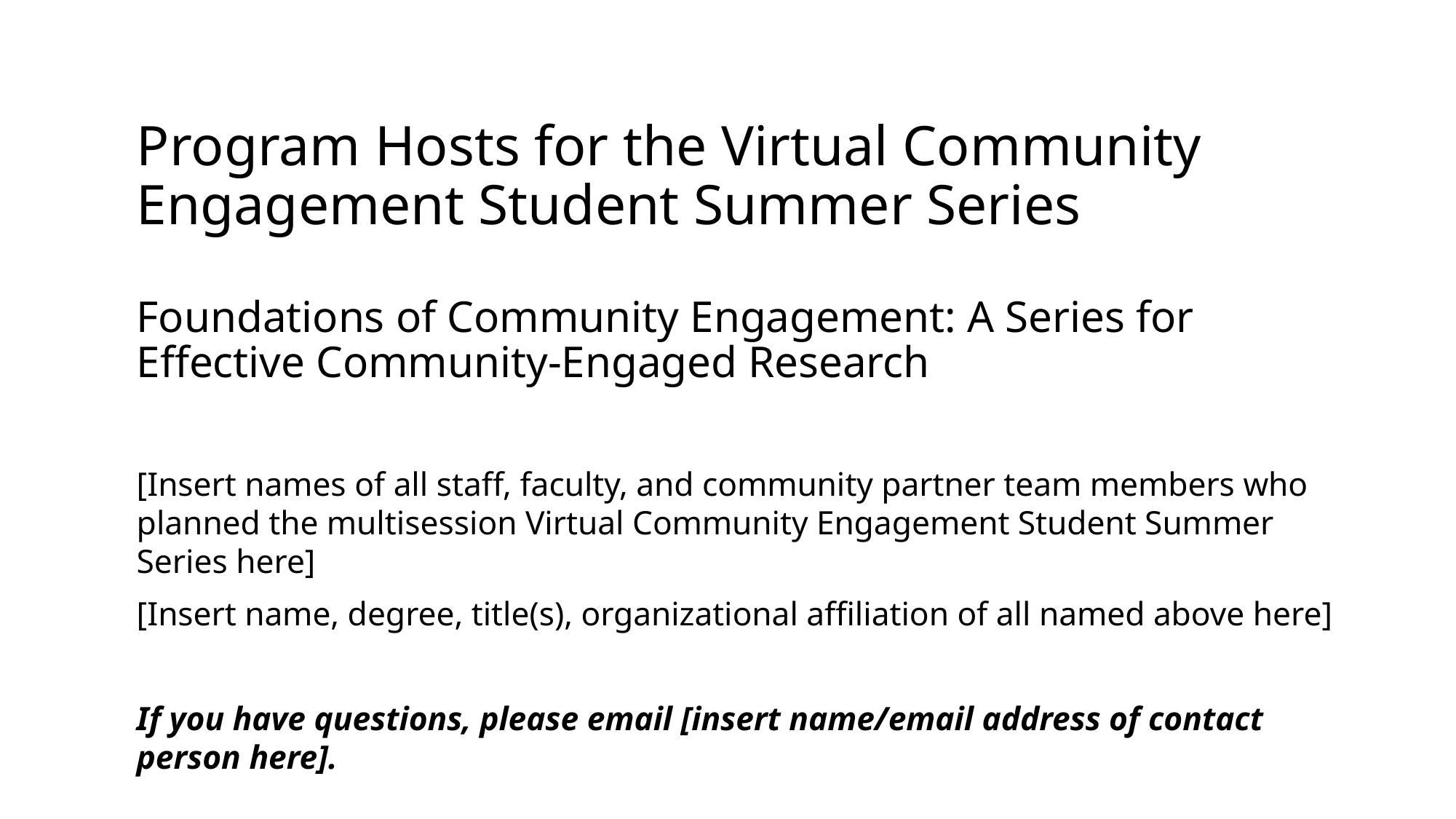

# Program Hosts for the Virtual Community Engagement Student Summer Series Foundations of Community Engagement: A Series for Effective Community-Engaged Research
[Insert names of all staff, faculty, and community partner team members who planned the multisession Virtual Community Engagement Student Summer Series here]
[Insert name, degree, title(s), organizational affiliation of all named above here]
If you have questions, please email [insert name/email address of contact person here].

## Slide 3
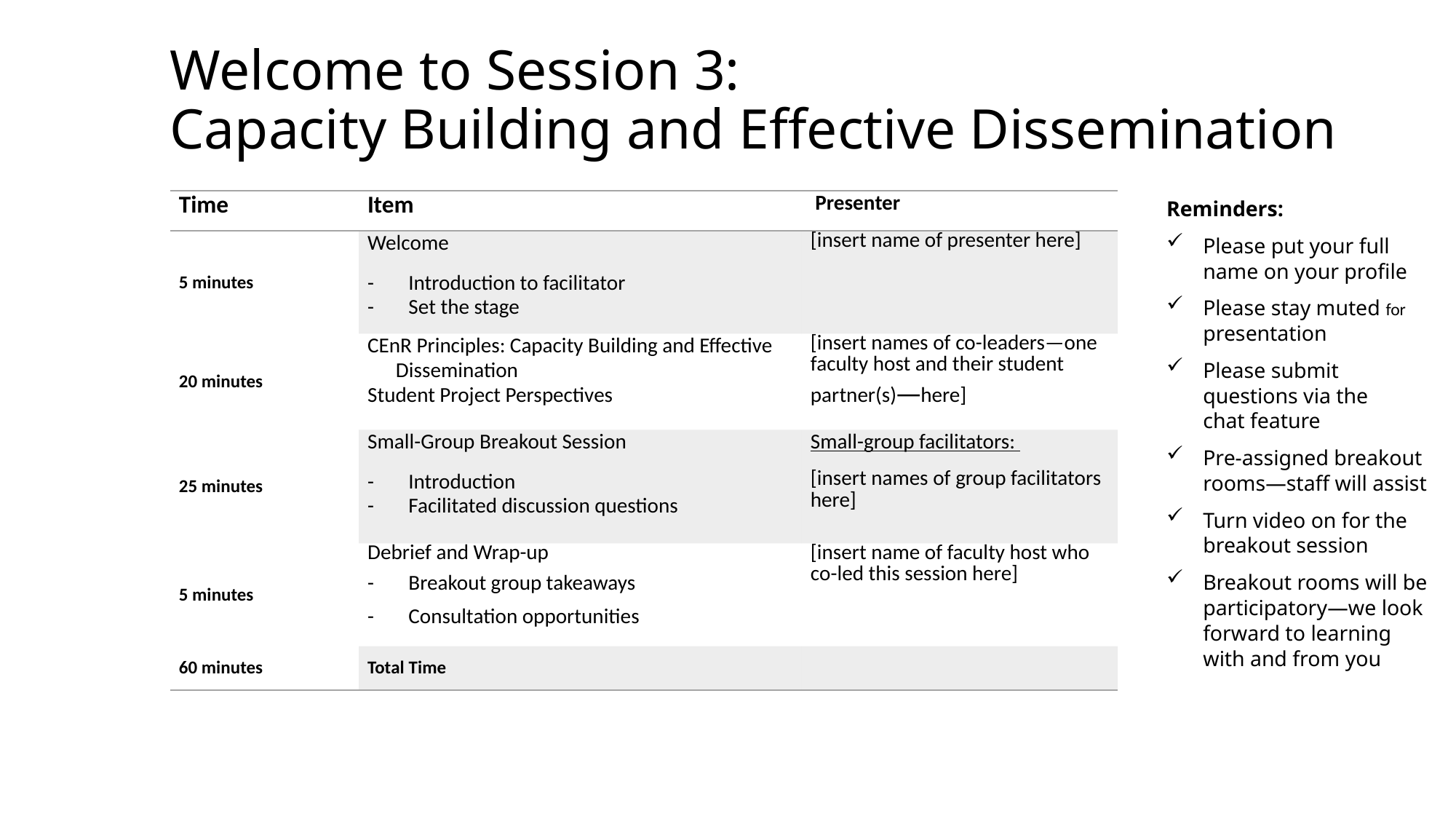

# Welcome to Session 3: Capacity Building and Effective Dissemination
| Time | Item | Presenter |
| --- | --- | --- |
| 5 minutes | Welcome Introduction to facilitator Set the stage | [insert name of presenter here] |
| 20 minutes | CEnR Principles: Capacity Building and Effective Dissemination Student Project Perspectives | [insert names of co-leaders—one faculty host and their student partner(s)—here] |
| 25 minutes | Small-Group Breakout Session Introduction Facilitated discussion questions | Small-group facilitators:  [insert names of group facilitators here] |
| 5 minutes | Debrief and Wrap-up Breakout group takeaways Consultation opportunities | [insert name of faculty host who co-led this session here] |
| 60 minutes | Total Time | |
Reminders:
Please put your full name on your profile
Please stay muted for presentation
Please submit questions via the chat feature
Pre-assigned breakout rooms—staff will assist
Turn video on for the breakout session
Breakout rooms will be participatory—we look forward to learning with and from you

## Slide 4
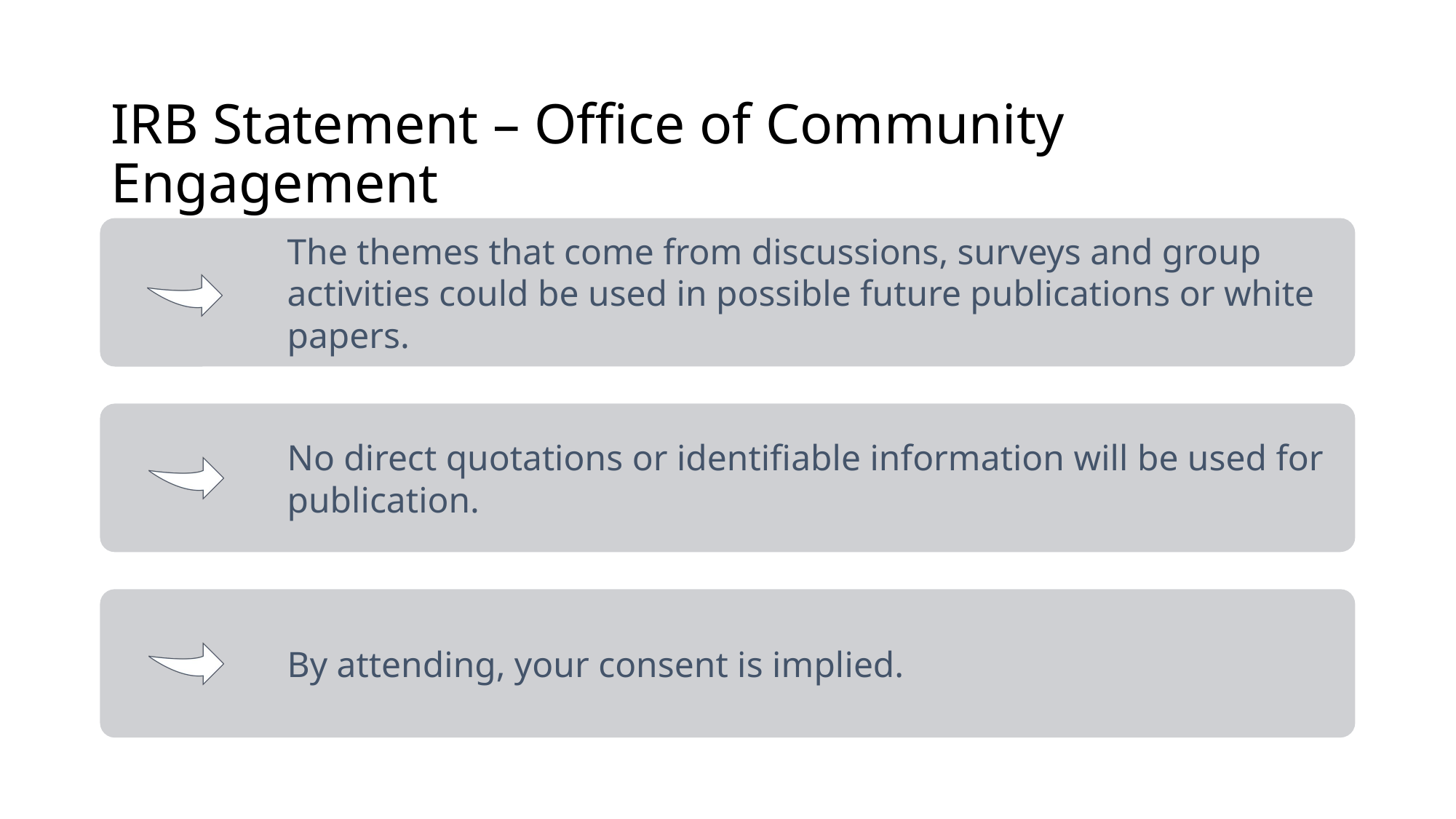

# IRB Statement – Office of Community Engagement

## Slide 5
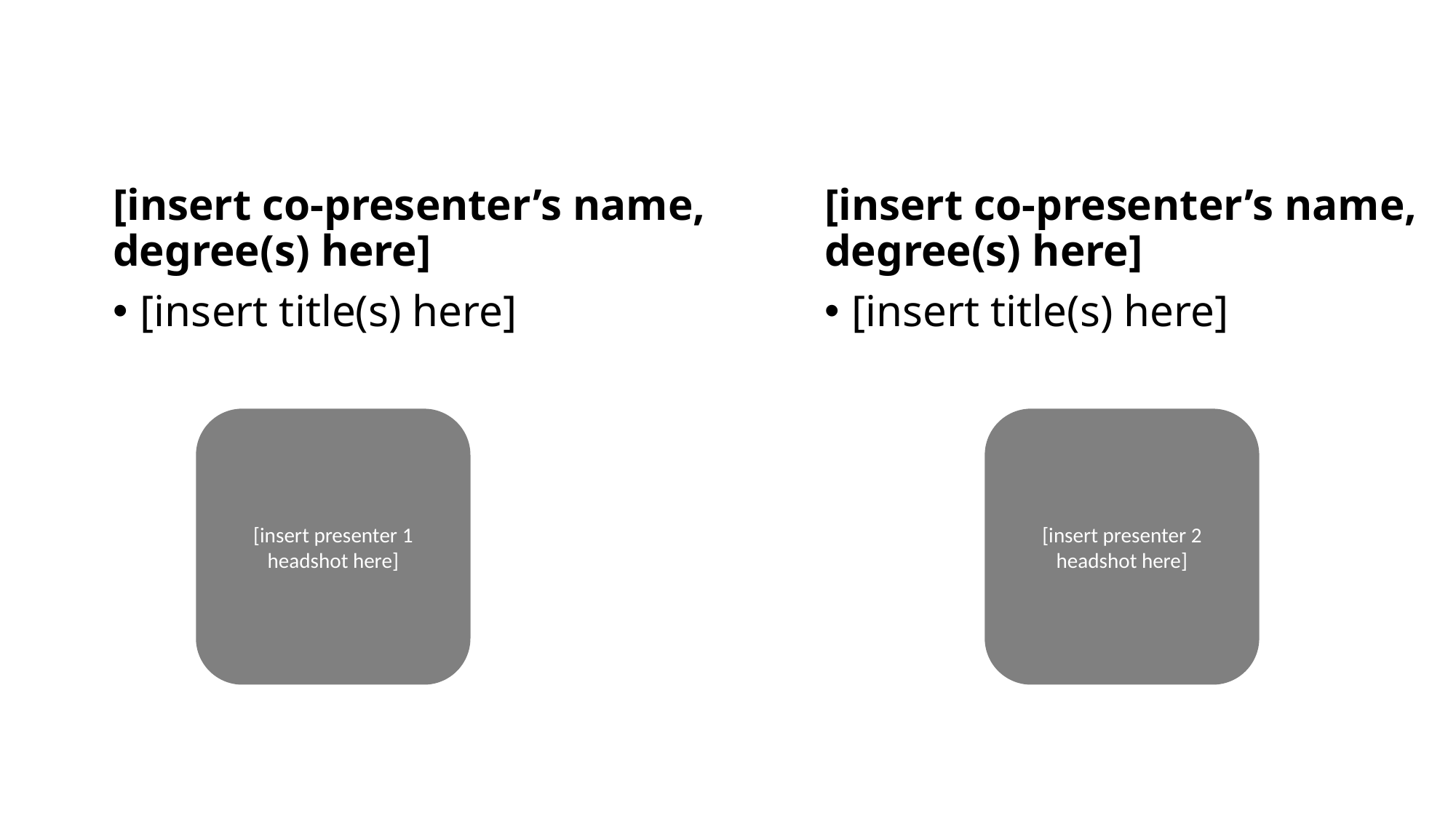

[insert co-presenter’s name, degree(s) here]
[insert title(s) here]
[insert co-presenter’s name, degree(s) here]
[insert title(s) here]
[insert presenter 1 headshot here]
[insert presenter 2 headshot here]

## Slide 6
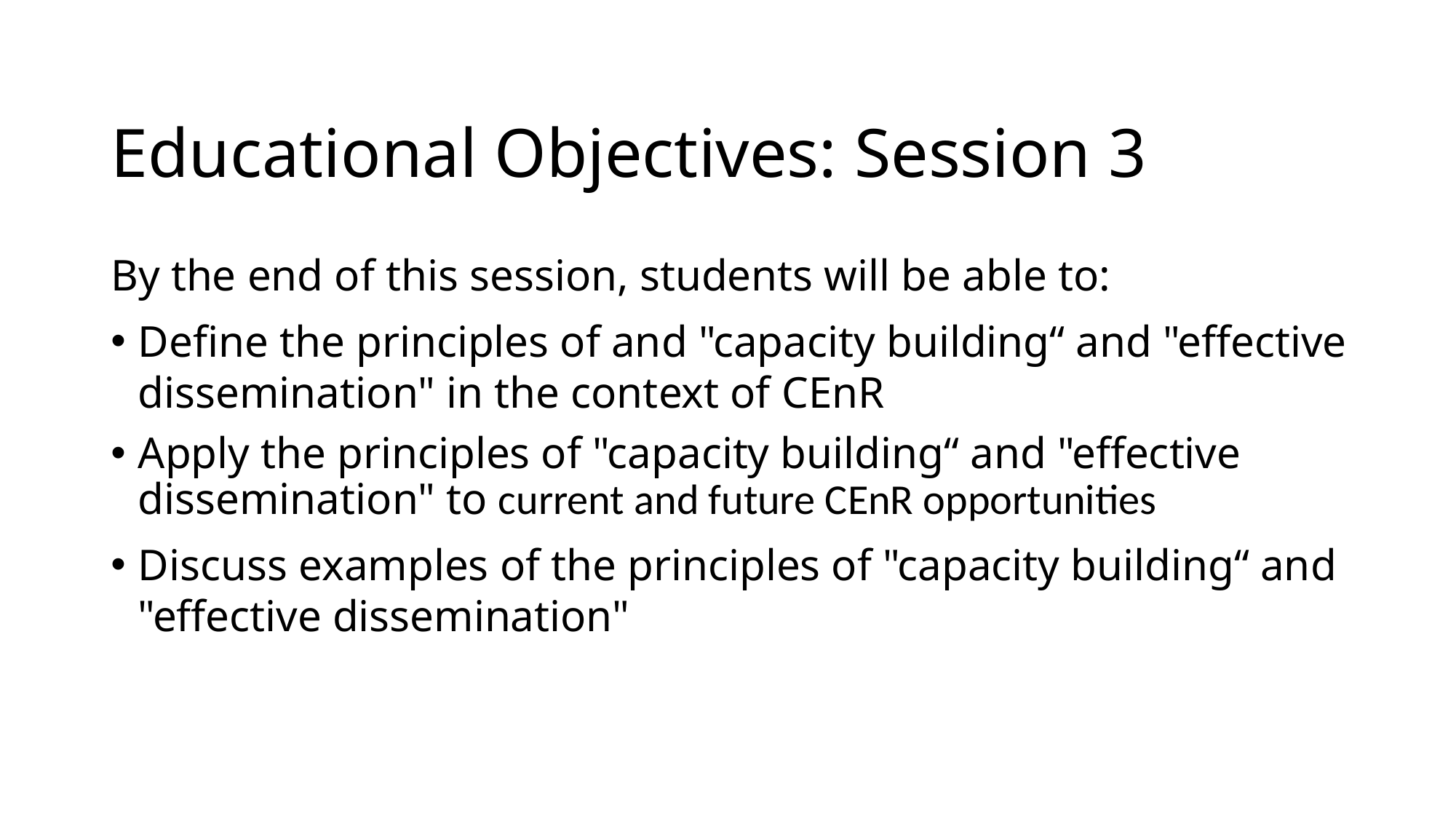

# Educational Objectives: Session 3
By the end of this session, students will be able to:
Define the principles of and "capacity building“ and "effective dissemination" in the context of CEnR
Apply the principles of "capacity building“ and "effective dissemination" to current and future CEnR opportunities
Discuss examples of the principles of "capacity building“ and "effective dissemination"

## Slide 7
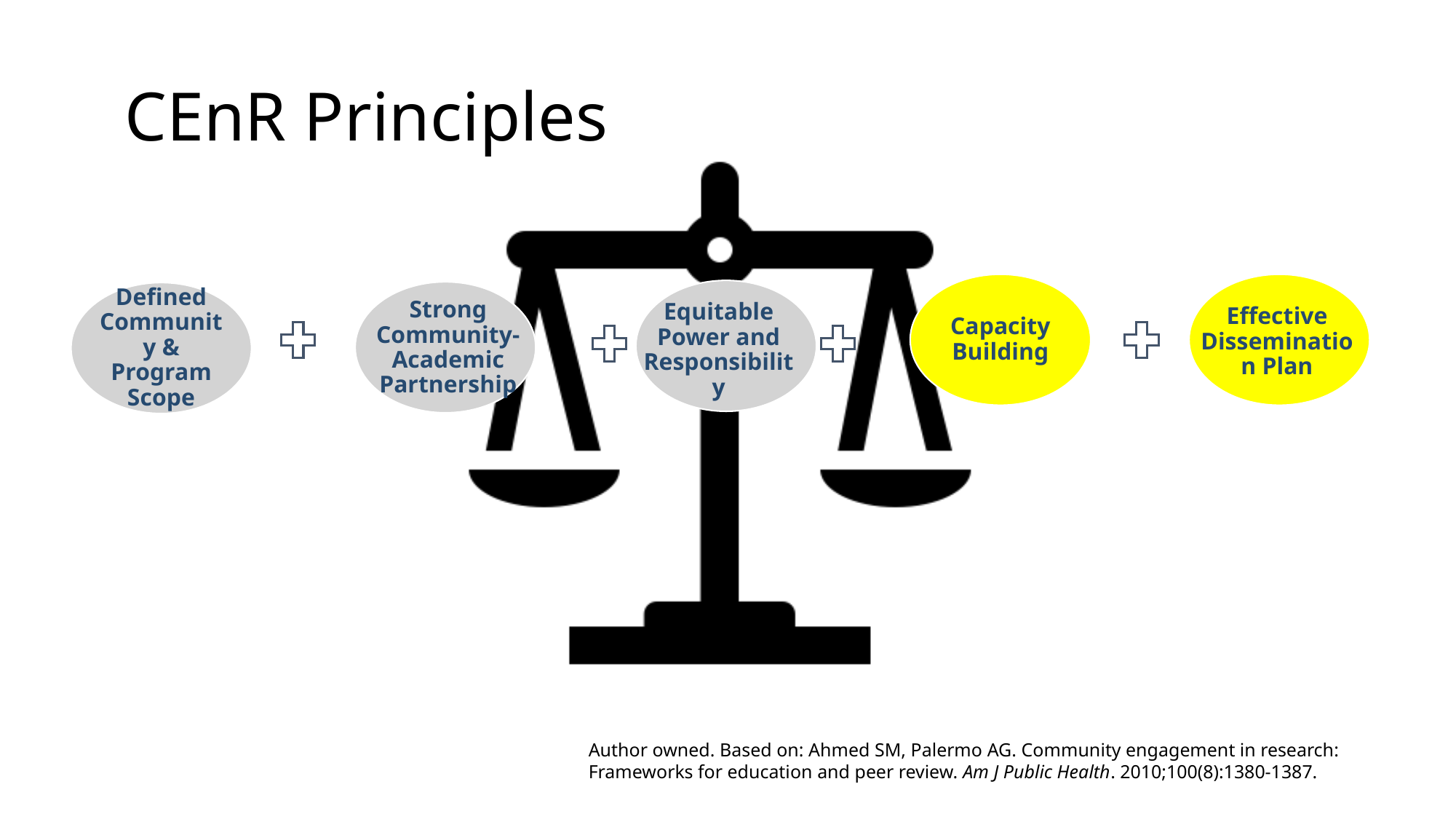

# CEnR Principles
Capacity Building
Effective Dissemination Plan
Equitable Power and Responsibility
Strong Community-Academic Partnership
Defined Community & Program Scope
Author owned. Based on: Ahmed SM, Palermo AG. Community engagement in research: Frameworks for education and peer review. Am J Public Health. 2010;100(8):1380-1387.

## Slide 8
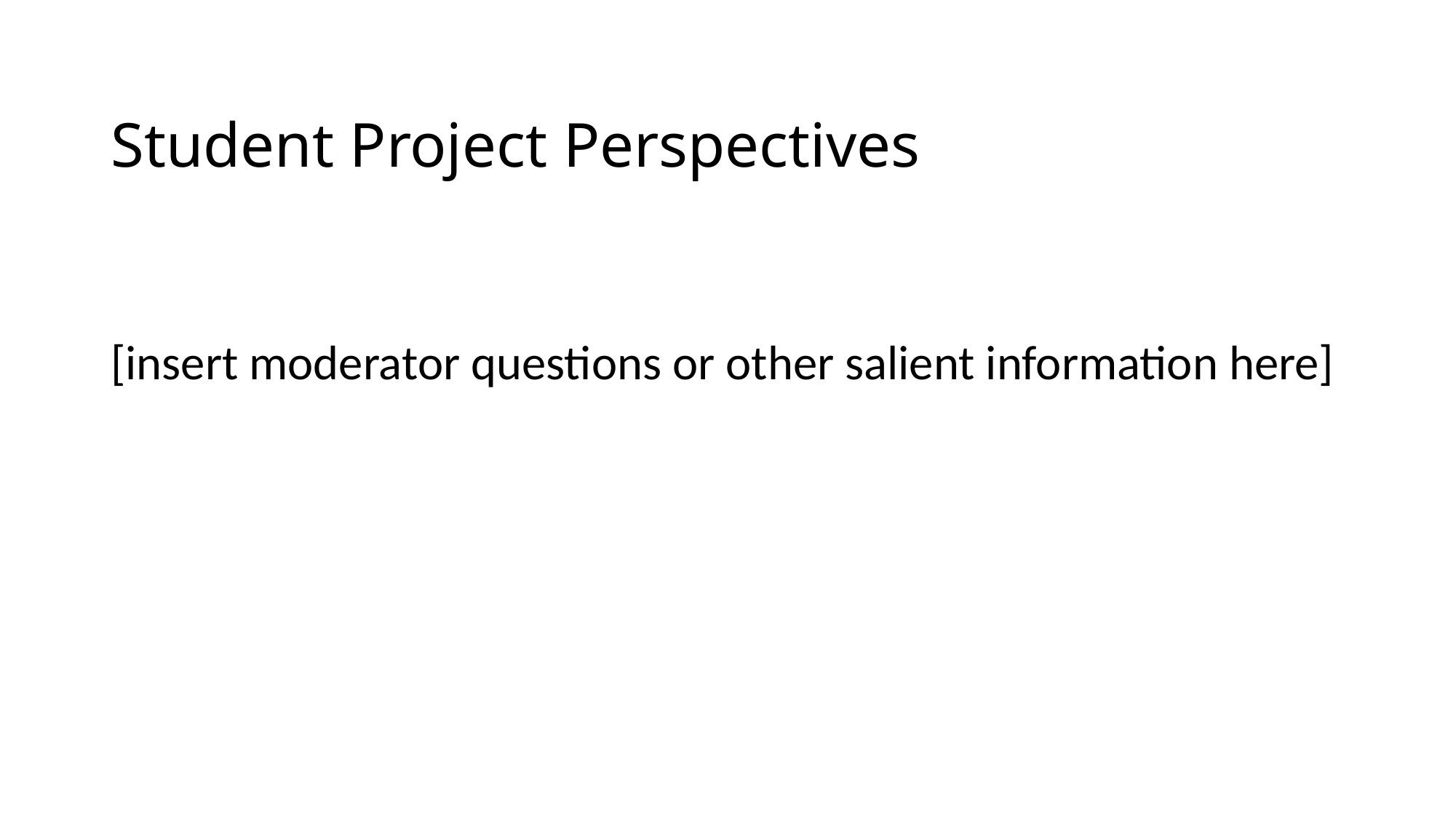

# Student Project Perspectives
[insert moderator questions or other salient information here]

## Slide 9
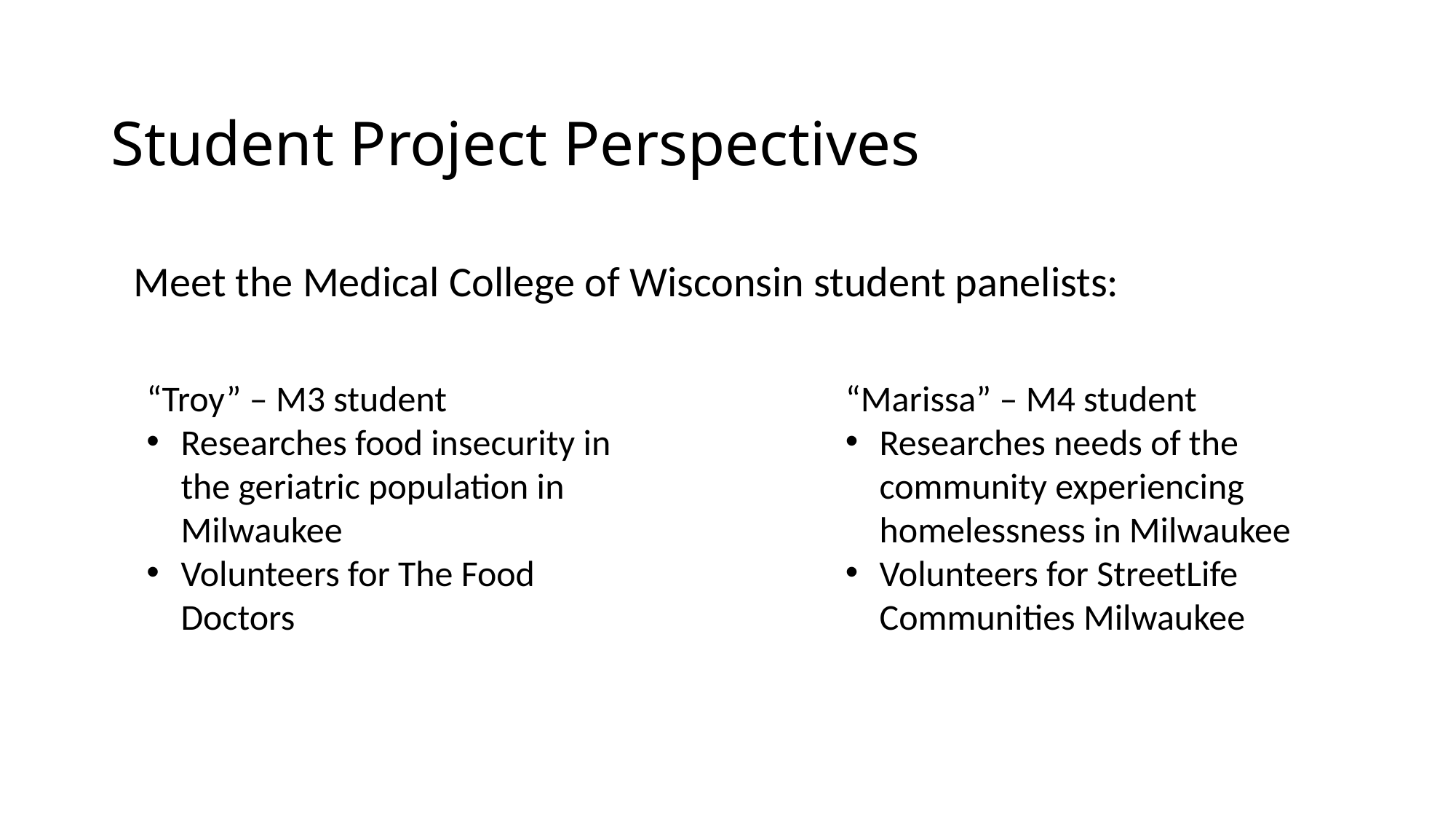

# Student Project Perspectives
Meet the Medical College of Wisconsin student panelists:
“Troy” – M3 student
Researches food insecurity in the geriatric population in Milwaukee
Volunteers for The Food Doctors
“Marissa” – M4 student
Researches needs of the community experiencing homelessness in Milwaukee
Volunteers for StreetLife Communities Milwaukee

## Slide 10
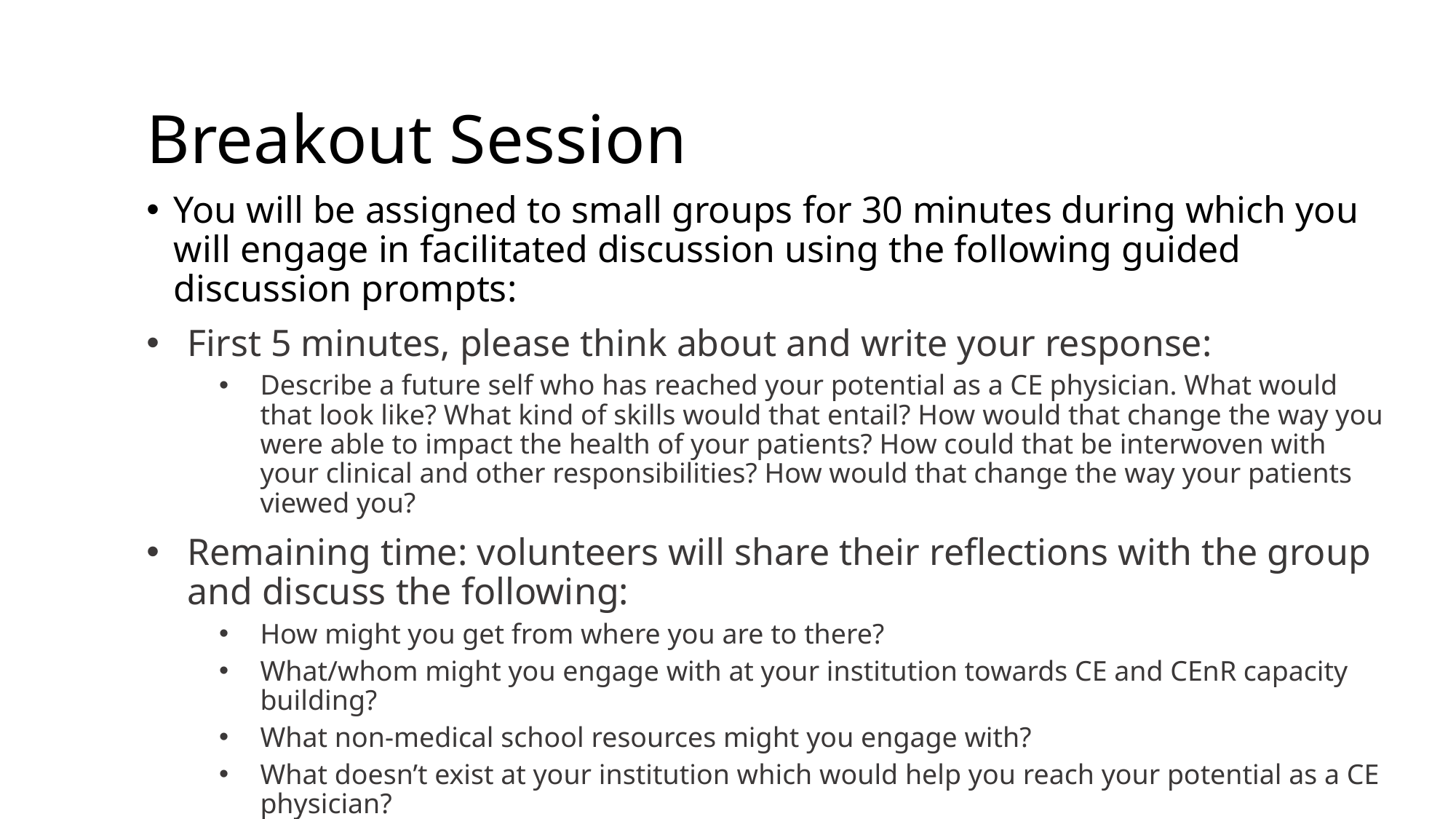

# Breakout Session
You will be assigned to small groups for 30 minutes during which you will engage in facilitated discussion using the following guided discussion prompts:
First 5 minutes, please think about and write your response:
Describe a future self who has reached your potential as a CE physician. What would that look like? What kind of skills would that entail? How would that change the way you were able to impact the health of your patients? How could that be interwoven with your clinical and other responsibilities? How would that change the way your patients viewed you?
Remaining time: volunteers will share their reflections with the group and discuss the following:
How might you get from where you are to there?
What/whom might you engage with at your institution towards CE and CEnR capacity building?
What non-medical school resources might you engage with?
What doesn’t exist at your institution which would help you reach your potential as a CE physician?
How can the [insert the name of your sponsoring department] help you reach your goals?

## Slide 11
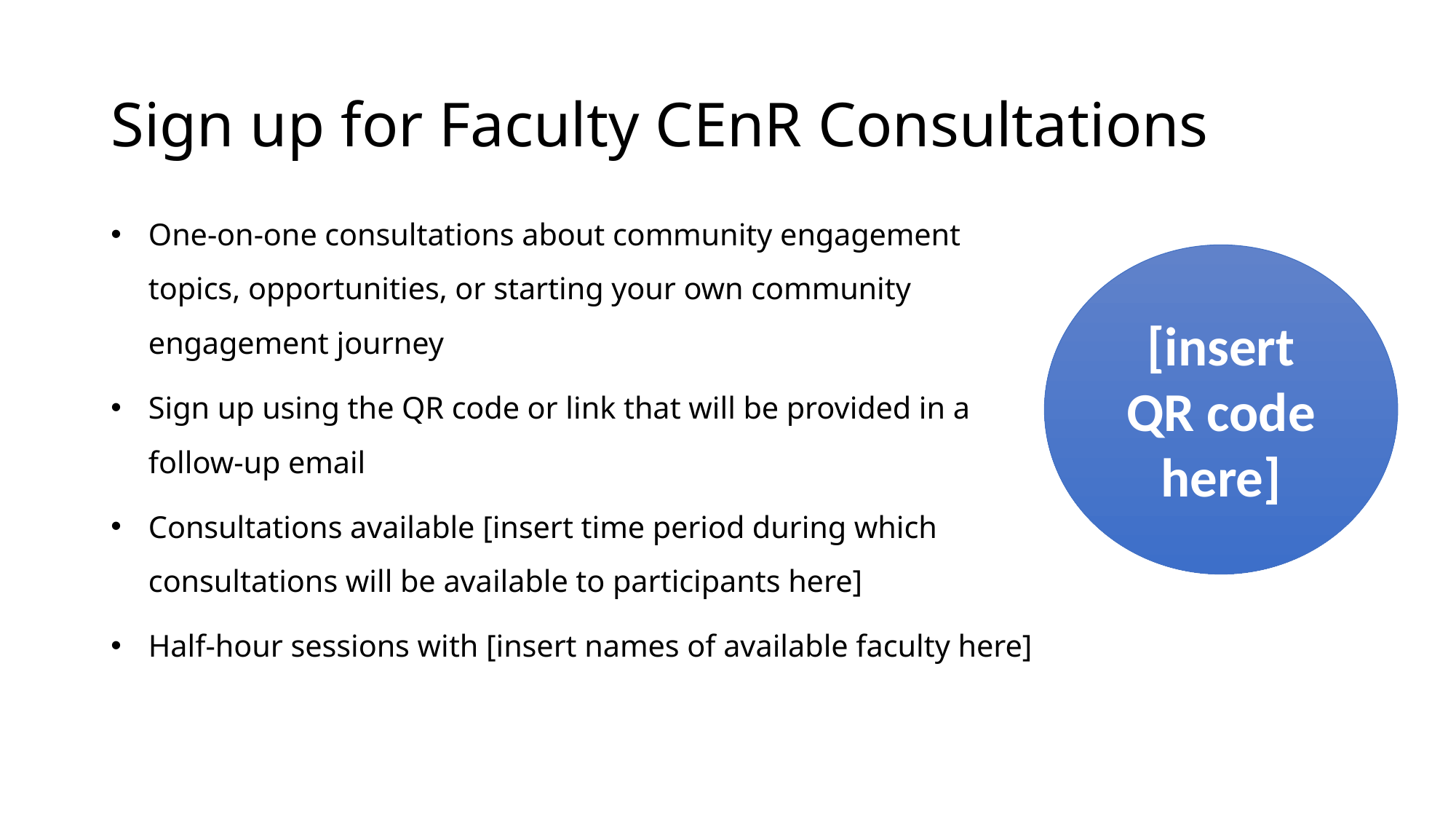

# Sign up for Faculty CEnR Consultations
One-on-one consultations about community engagement topics, opportunities, or starting your own community engagement journey
Sign up using the QR code or link that will be provided in a follow-up email
Consultations available [insert time period during which consultations will be available to participants here]
Half-hour sessions with [insert names of available faculty here]
[insert QR code here]

## Slide 12
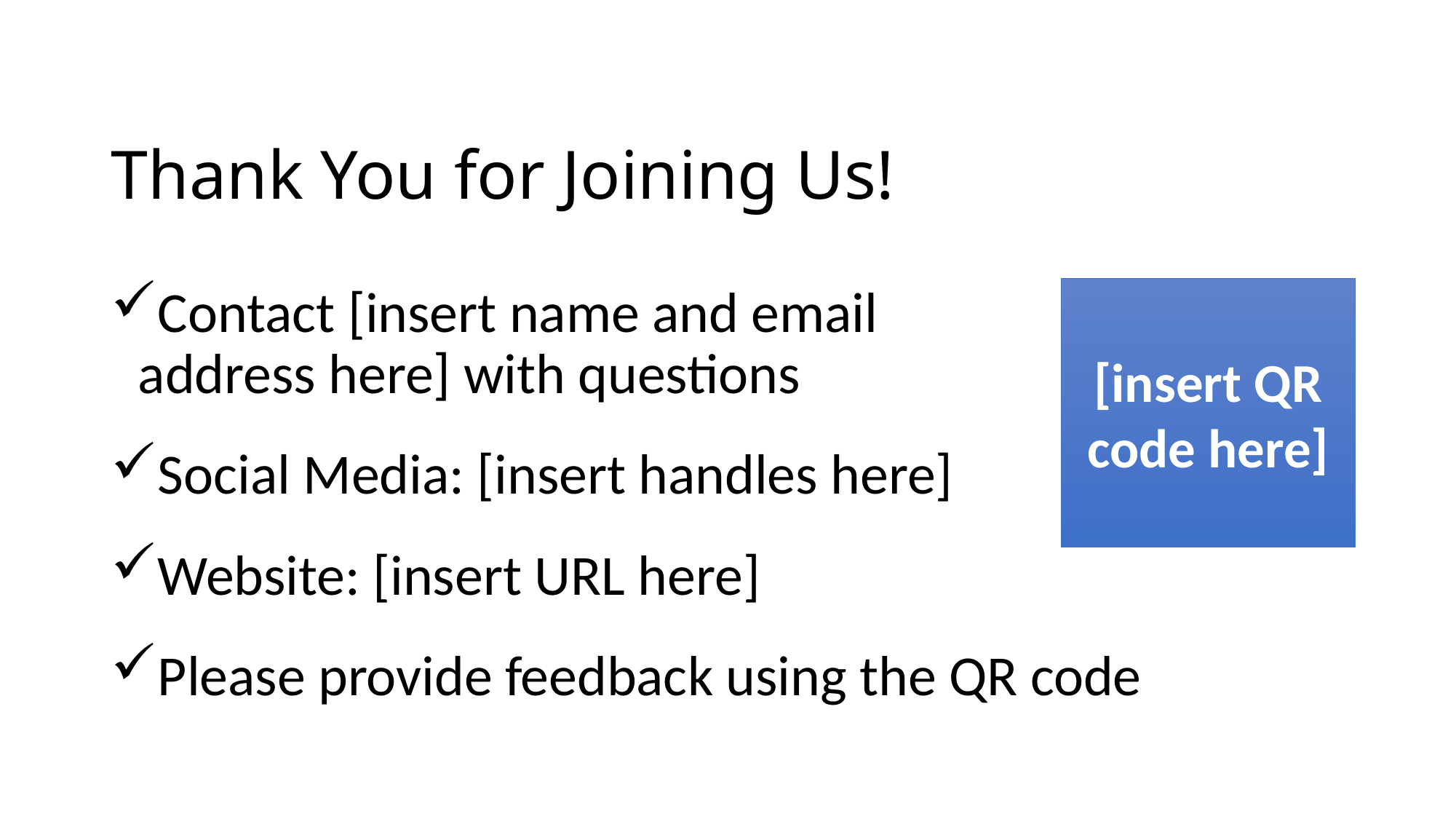

# Thank You for Joining Us!
Contact [insert name and email
address here] with questions
Social Media: [insert handles here]
Website: [insert URL here]
Please provide feedback using the QR code
[insert QR code here]

## Slide 13
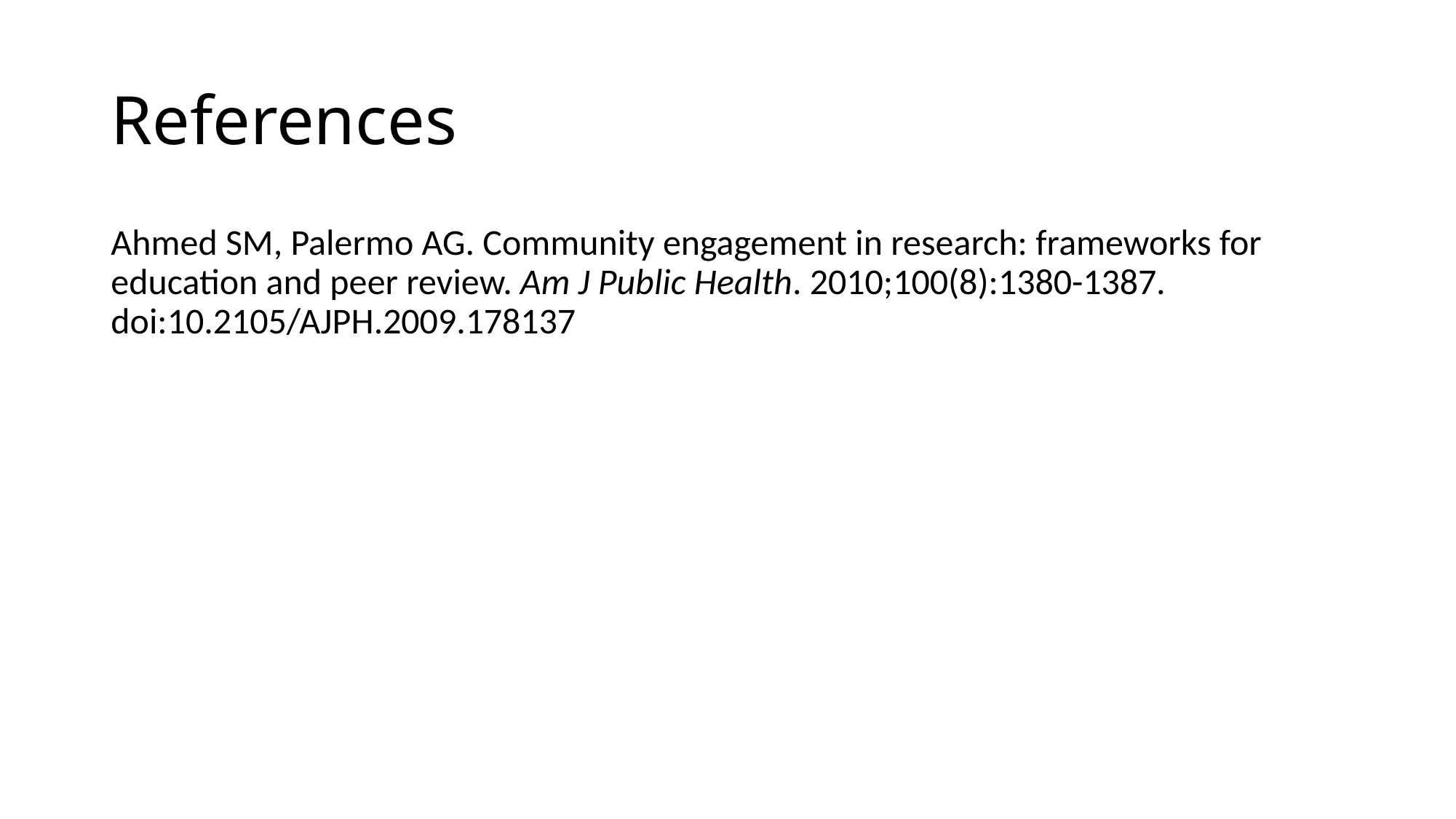

# References
Ahmed SM, Palermo AG. Community engagement in research: frameworks for education and peer review. Am J Public Health. 2010;100(8):1380-1387. doi:10.2105/AJPH.2009.178137
