# Supplementary material for: Foundations of Community Engagement: A Series for Effective Community-Engaged Research
Source: MedEdPORTAL. 2023 Oct 10;19:11350. doi: 10.15766/mep_2374-8265.11350 (PMC10562524; doi:10.15766/mep_2374-8265.11350)
Supplement: Supplementary file 1 — CE Didactic Session Slides.pptxApplication for Small-Group Series.docxCommunity-Academic Partnership Slides.pptxEquitable Power and Responsibility Slides.pptxEquitable Power and Responsibility Case Studies.docxCapacity Building and Dissemination Slides.pptxFacilitator Guide.docxCE Didactic Session Evaluation.docxSmall-Group Session Evaluation.docx [file mep_2374-8265.11350-s001.zip › I. Small-Group Session Evaluation.docx]

Virtual Community Engagement Student Summer Series Evaluation

Thank you very much for participating in the Virtual Community Engagement Student Summer Series offered by [insert institutional affiliations here].

Your feedback is important to us! Please respond to the questions below. Your answers will remain anonymous unless you choose to provide your name and email address for further discussion.

Q1 I found this session worthwhile.

- Strongly agree
- Somewhat agree
- Neither agree nor disagree
- Somewhat disagree
- Strongly disagree

Q2 I learned something that I will use in my practice/profession.

- Strongly agree
- Somewhat agree
- Neither agree nor disagree
- Somewhat disagree
- Strongly disagree

Q3 Overall the speakers were effective at communicating session content.

- Strongly agree
- Somewhat agree
- Neither agree nor disagree
- Somewhat disagree
- Strongly disagree

Display this question (Q4) if answer to Q3 is:

If Overall the speakers were effective at communicating session content. = Somewhat agree

Or Overall the speakers were effective at communicating session content. = Somewhat disagree

Or Overall the speakers were effective at communicating session content. = Strongly disagree

Or Overall the speakers were effective at communicating session content. = Neither agree nor disagree

Q4 Please explain how you felt about the speakers:

________________________________________________________________

Q5 I found the small group discussion worthwhile.

- Strongly agree
- Somewhat agree
- Neither agree nor disagree
- Somewhat disagree
- Strongly disagree

Display this question (Q6) if answer to Q5 is:

If I found the small group discussion worthwhile. = Somewhat agree

Or I found the small group discussion worthwhile. = Neither agree nor disagree

Or I found the small group discussion worthwhile. = Somewhat disagree

Or I found the small group discussion worthwhile. = Strongly disagree

Q6 Please explain what could have been done better:

________________________________________________________________

Q7 Please indicate who facilitated your small group discussion:

- [insert name of facilitator 1 here]
- [insert name of facilitator 2 here]
- [insert name of facilitator 3 here]

Q8 On a technical level, how did the platform work for you?

- I had no technical difficulties and was very satisfied with the platform.
- I had no technical difficulties, but I wish the format had been different.
- I had a few technical difficulties, but I was still able to participate fully.
- I had major technical difficulties that limited my ability to participate fully.

Display this question (Q9) if answer to Q8 is:

If On a technical level, how did the platform work for you? = I had no technical difficulties, but I wish the format had been different.

Q9 Please explain what could have been done better:

________________________________________________________________

Display this question (Q10) if answer to Q8 is:

If On a technical level, how did the platform work for you? = I had major technical difficulties that limited my ability to participate fully.

Q10 We are sorry the platform did not work well for you! Please describe the difficulties you had:

________________________________________________________________

Q11 Would you be interested in continuing discussion on this topic beyond this session?

- Yes
- No

Display these questions (Q12, Q13, Q14) if answer to Q11 is:

If Would you be interested in continuing discussion on this topic beyond this session? = Yes

Q12 Which of the following outlets would be of interest to you? (Select all that apply)

- Curated readings
- Discussion group
- Discussion board or social media group
- Private reflective exercises
- Public reflective exercises
- Peer-to-peer conversations
- Engagement with presenters
- Other ________________________________________________

Q13 Please provide your first and last name so we may identify you:

________________________________________________________________

Q14 Please provide your email address:

________________________________________________________________

Q15 Please use the space below to share any other thoughts about the session, including ways we can improve future sessions:

________________________________________________________________

Thank you for your feedback!
