# Supplementary material for: Foundations of Community Engagement: A Series for Effective Community-Engaged Research
Source: MedEdPORTAL. 2023 Oct 10;19:11350. doi: 10.15766/mep_2374-8265.11350 (PMC10562524; doi:10.15766/mep_2374-8265.11350)
Supplement: Supplementary file 1 — CE Didactic Session Slides.pptxApplication for Small-Group Series.docxCommunity-Academic Partnership Slides.pptxEquitable Power and Responsibility Slides.pptxEquitable Power and Responsibility Case Studies.docxCapacity Building and Dissemination Slides.pptxFacilitator Guide.docxCE Didactic Session Evaluation.docxSmall-Group Session Evaluation.docx [file mep_2374-8265.11350-s001.zip › A. CE Didactic Session Slides.pptx]

## Slide 1
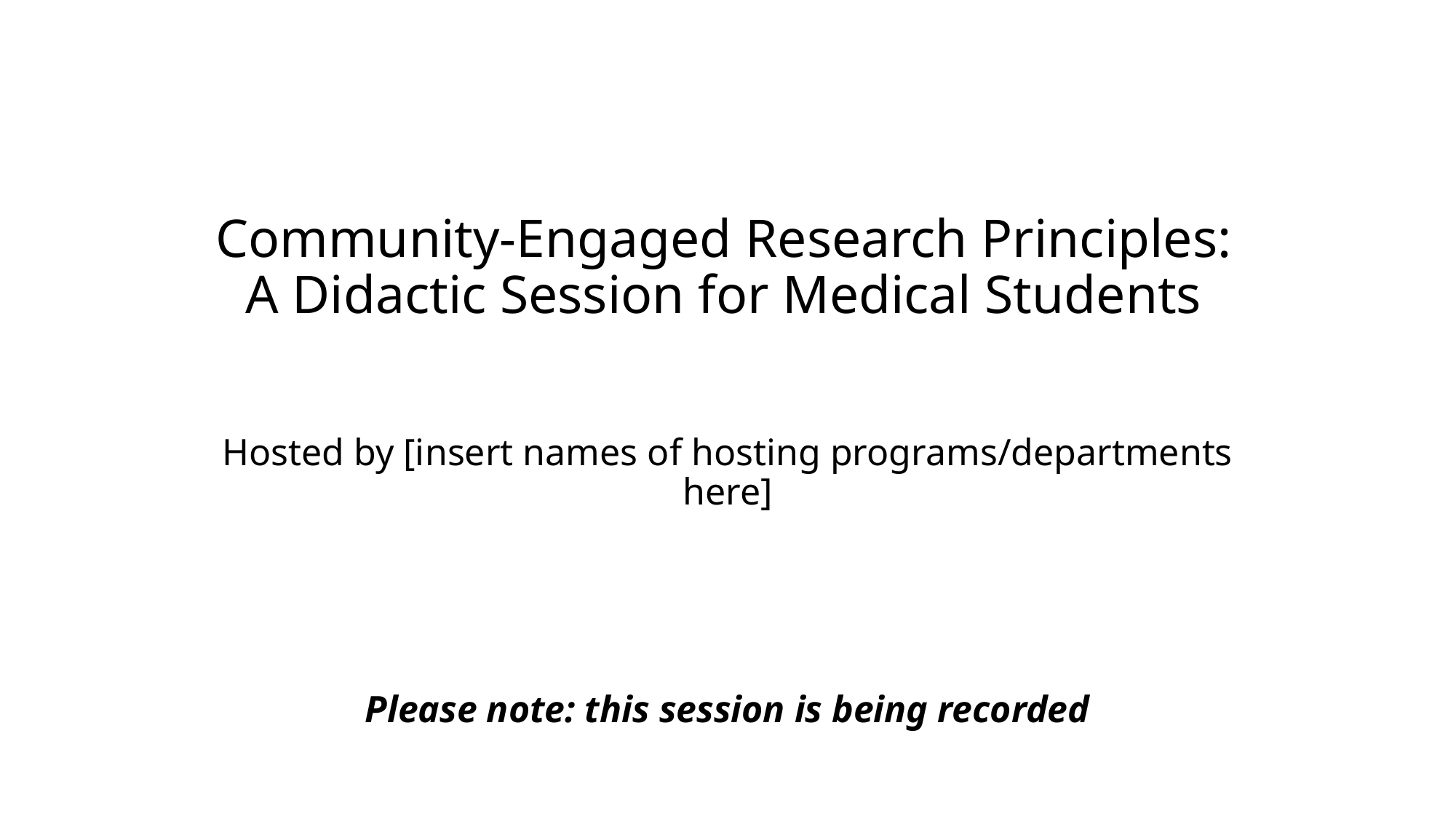

# Community-Engaged Research Principles: A Didactic Session for Medical Students
Hosted by [insert names of hosting programs/departments here]
Please note: this session is being recorded

## Slide 2
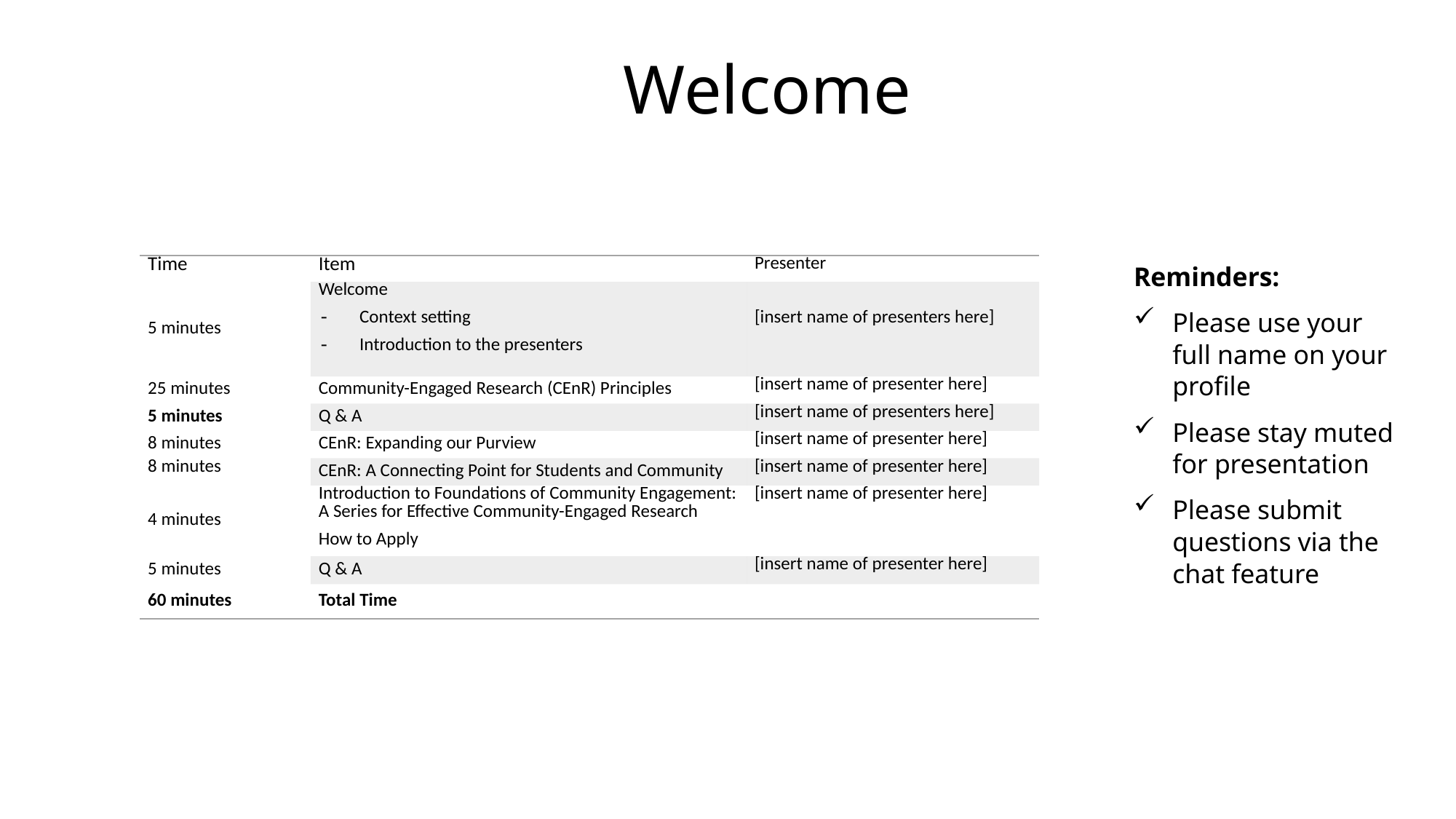

# Welcome
| Time | Item | Presenter |
| --- | --- | --- |
| 5 minutes | Welcome Context setting Introduction to the presenters | [insert name of presenters here] |
| 25 minutes | Community-Engaged Research (CEnR) Principles | [insert name of presenter here] |
| 5 minutes | Q & A | [insert name of presenters here] |
| 8 minutes | CEnR: Expanding our Purview | [insert name of presenter here] |
| 8 minutes | CEnR: A Connecting Point for Students and Community | [insert name of presenter here] |
| 4 minutes | Introduction to Foundations of Community Engagement: A Series for Effective Community-Engaged Research How to Apply | [insert name of presenter here] |
| 5 minutes | Q & A | [insert name of presenter here] |
| 60 minutes | Total Time | |
Reminders:
Please use your full name on your profile
Please stay muted for presentation
Please submit questions via the chat feature

## Slide 3
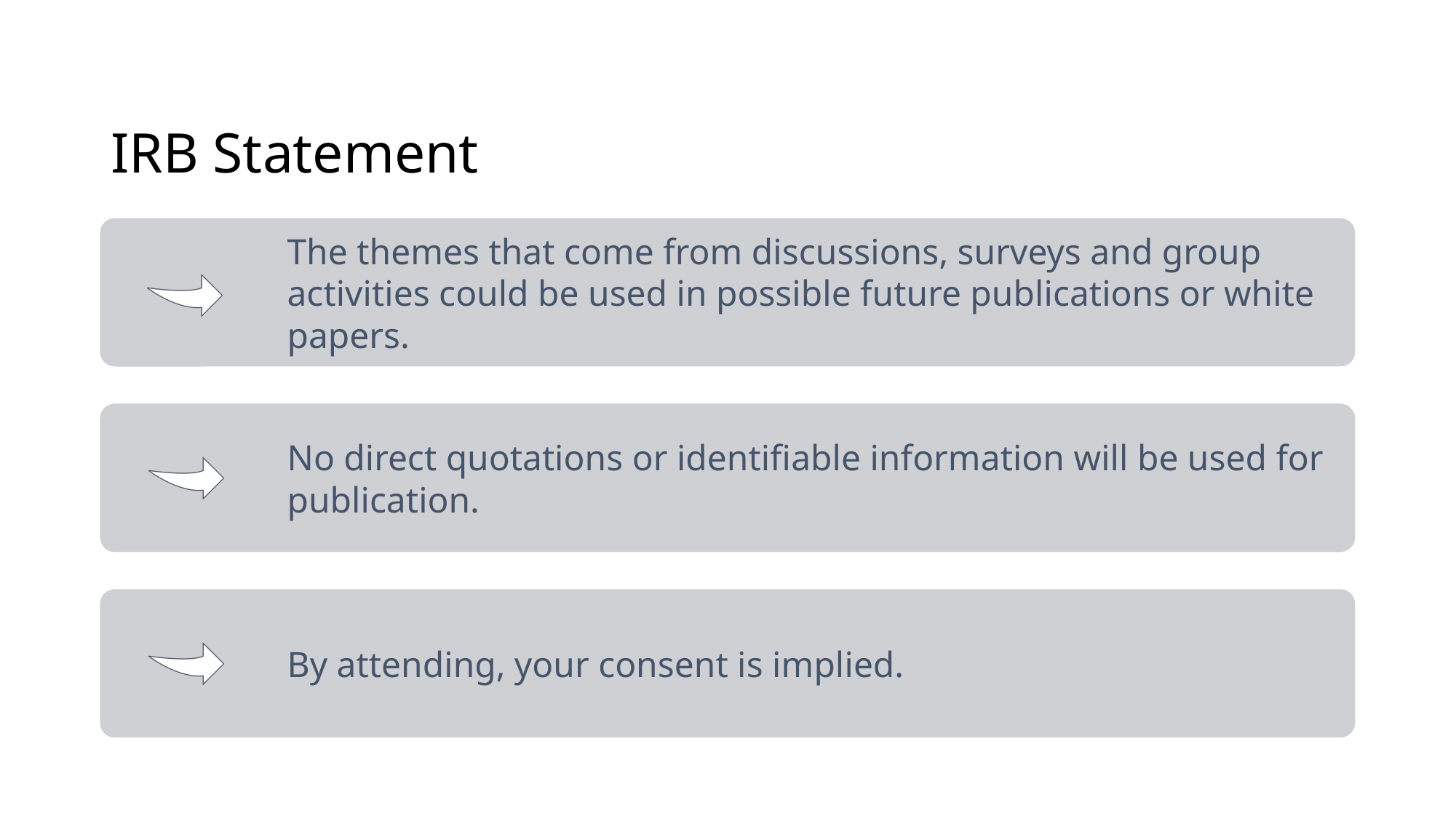

# IRB Statement

## Slide 4
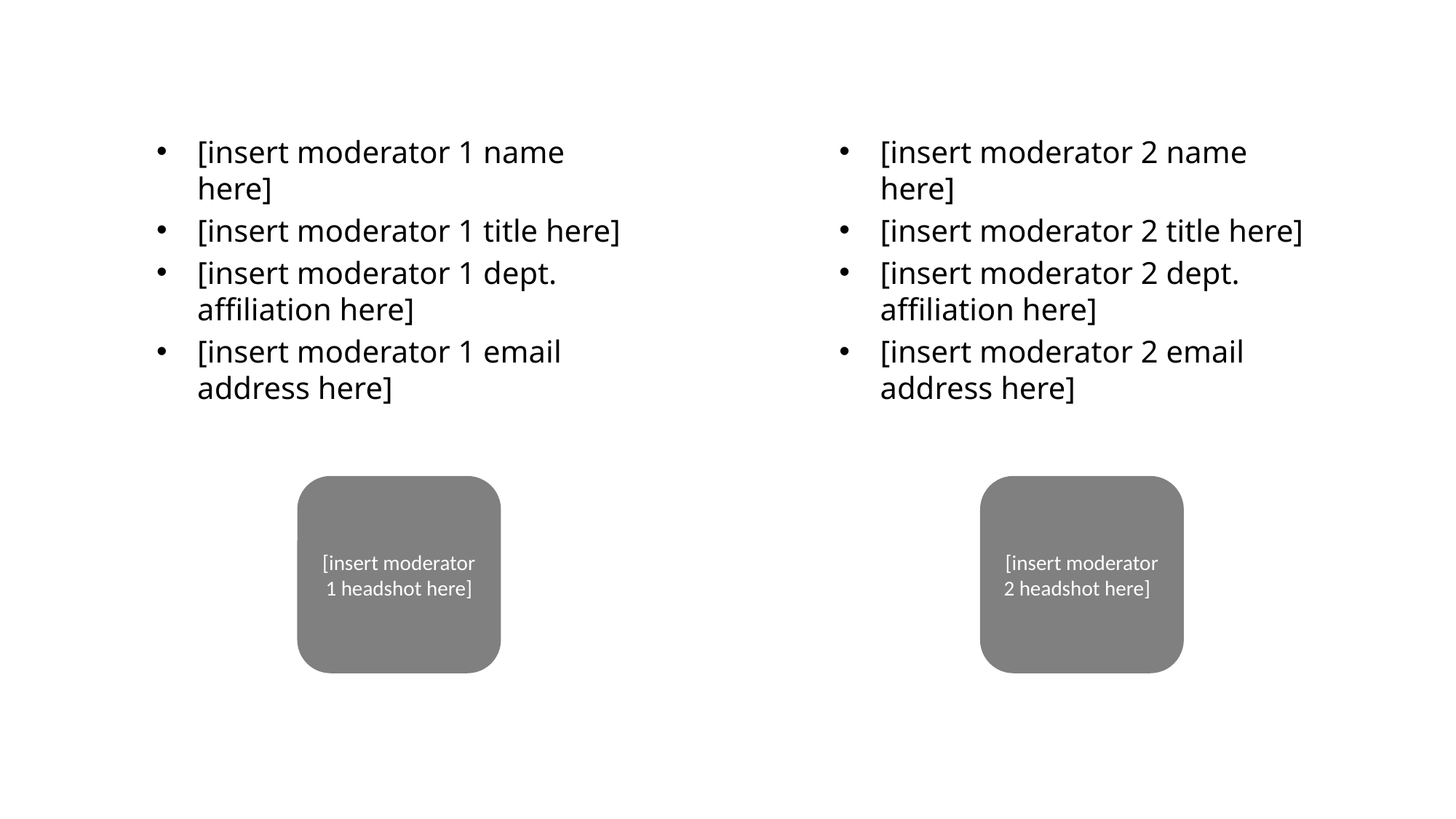

[insert moderator 1 name here]
[insert moderator 1 title here]
[insert moderator 1 dept. affiliation here]
[insert moderator 1 email address here]
[insert moderator 2 name here]
[insert moderator 2 title here]
[insert moderator 2 dept. affiliation here]
[insert moderator 2 email address here]
[insert moderator 1 headshot here]
[insert moderator 2 headshot here]

## Slide 5
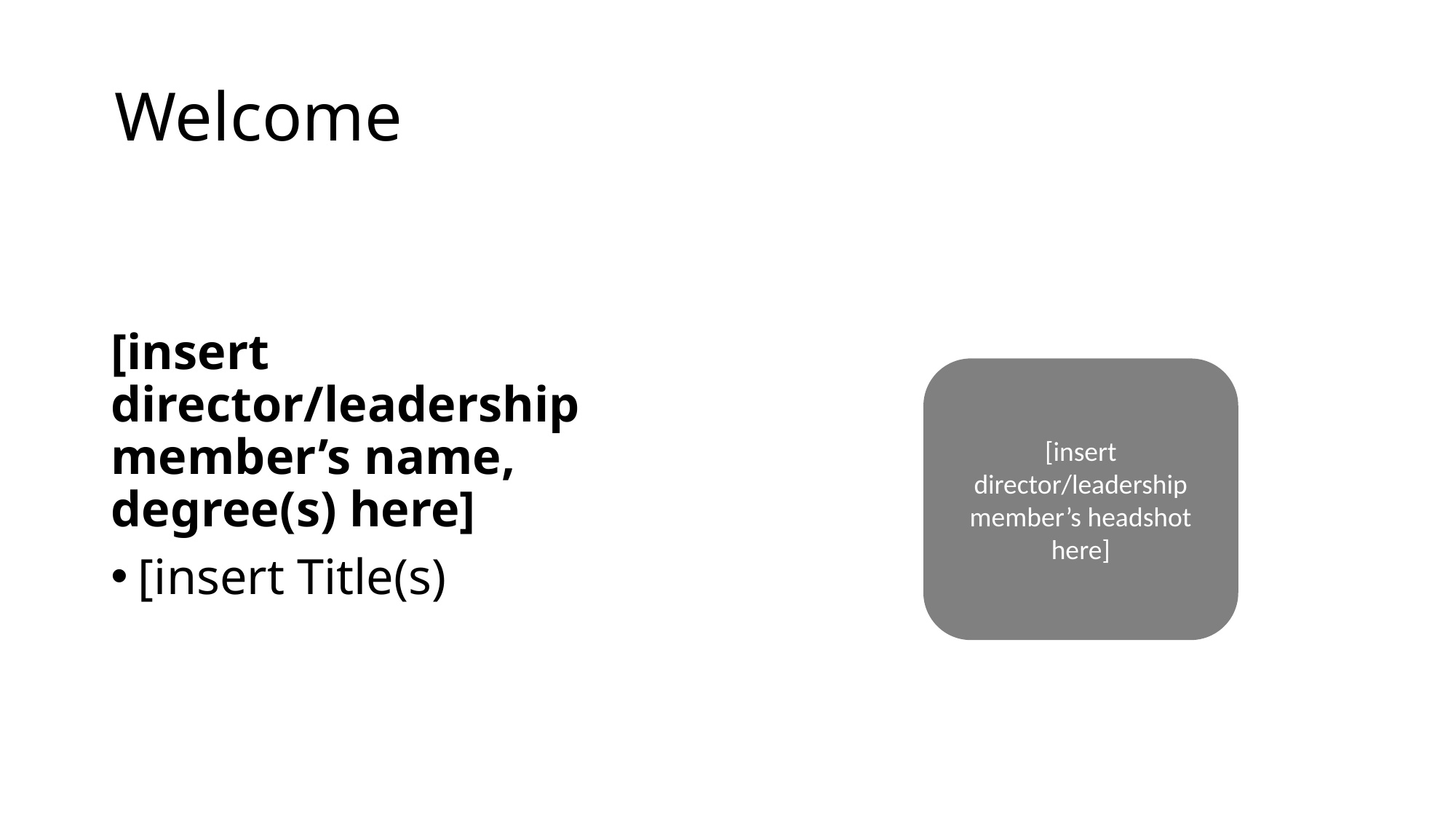

# Welcome
[insert director/leadership member’s name, degree(s) here]
[insert Title(s)
[insert director/leadership member’s headshot here]

## Slide 6
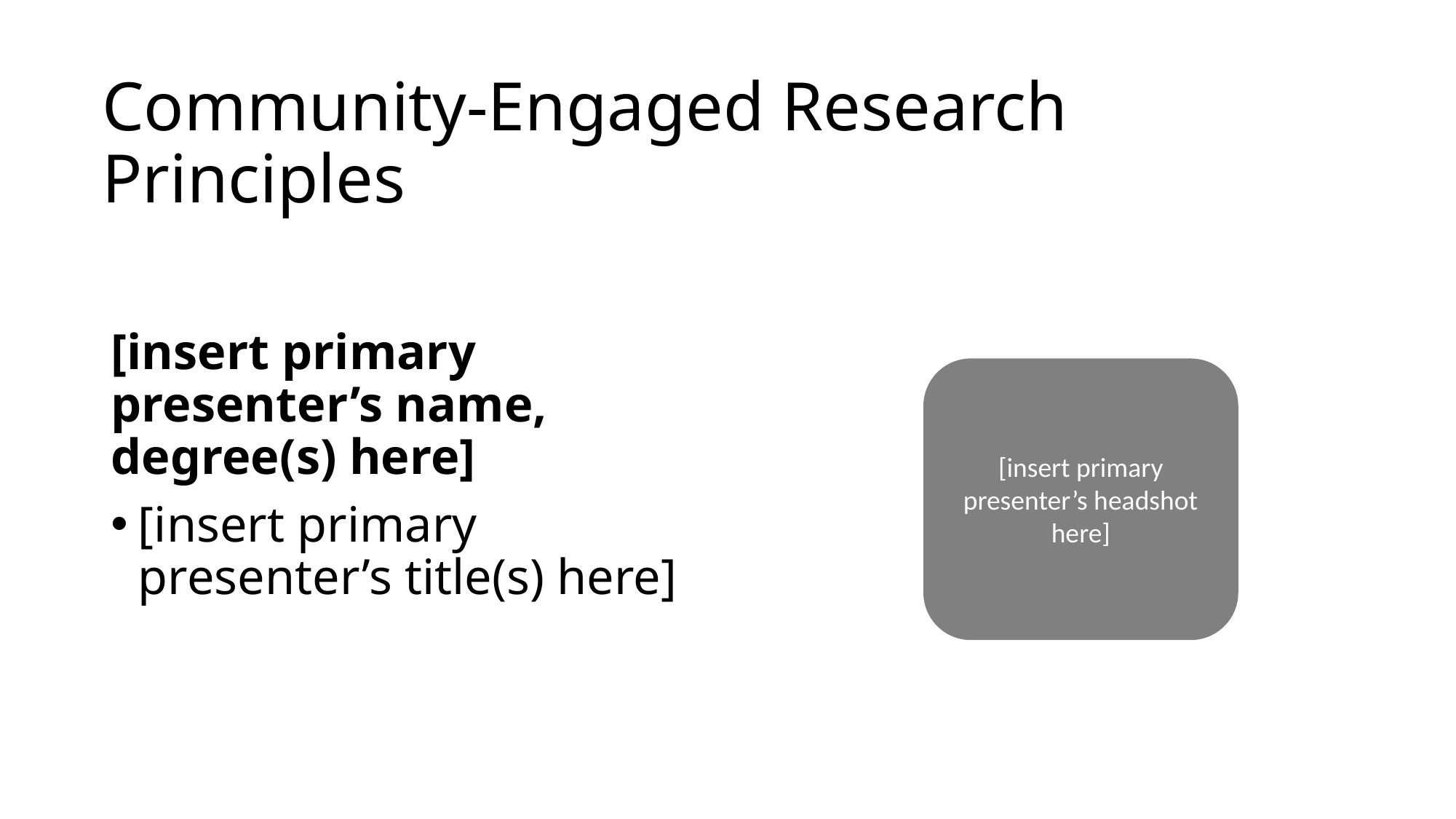

# Community-Engaged Research Principles
[insert primary presenter’s name, degree(s) here]
[insert primary presenter’s title(s) here]
[insert primary presenter’s headshot here]

## Slide 7
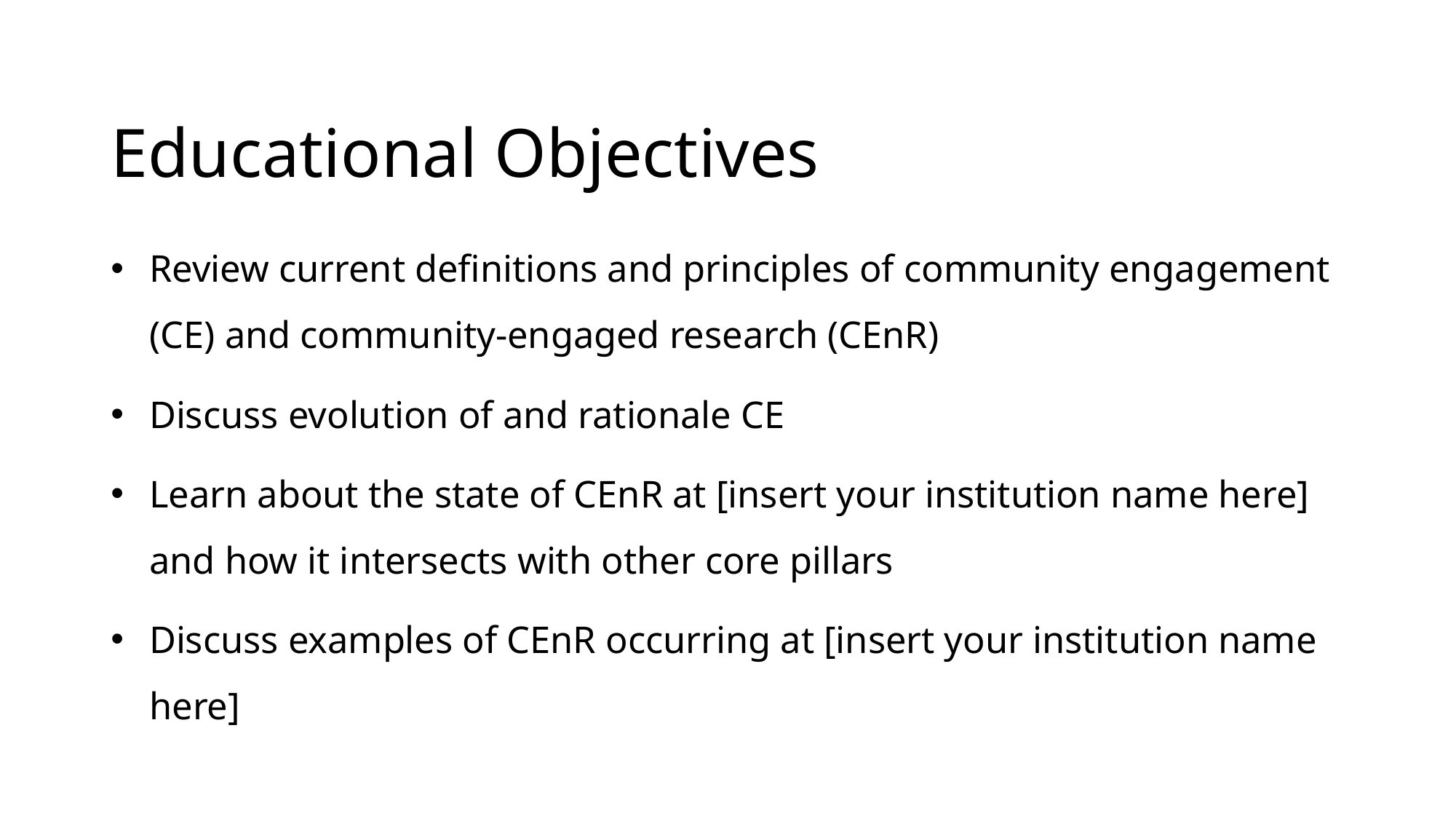

# Educational Objectives
Review current definitions and principles of community engagement (CE) and community-engaged research (CEnR)
Discuss evolution of and rationale CE
Learn about the state of CEnR at [insert your institution name here] and how it intersects with other core pillars
Discuss examples of CEnR occurring at [insert your institution name here]

## Slide 8
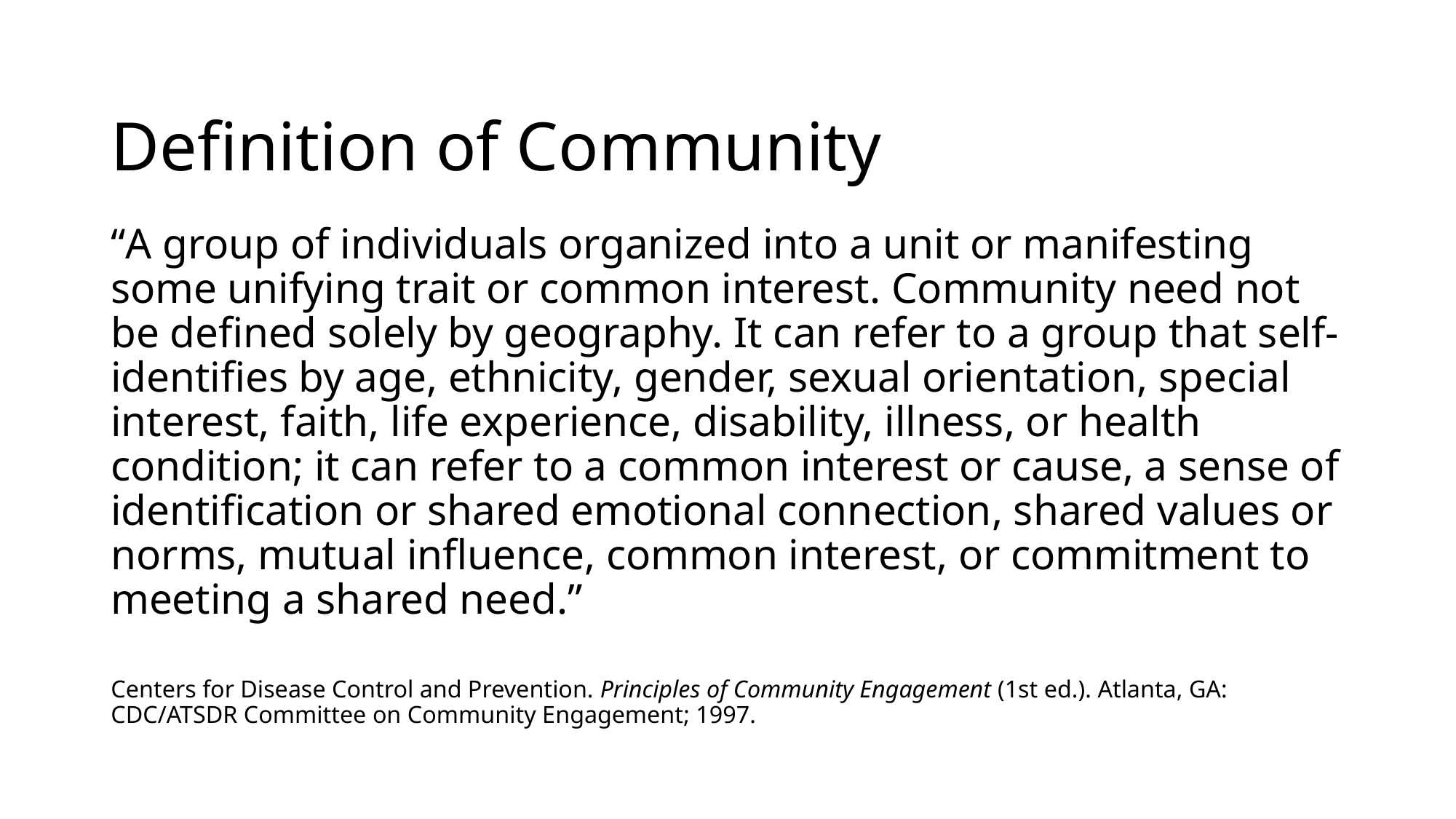

# Definition of Community
“A group of individuals organized into a unit or manifesting some unifying trait or common interest. Community need not be defined solely by geography. It can refer to a group that self-identifies by age, ethnicity, gender, sexual orientation, special interest, faith, life experience, disability, illness, or health condition; it can refer to a common interest or cause, a sense of identification or shared emotional connection, shared values or norms, mutual influence, common interest, or commitment to meeting a shared need.”
Centers for Disease Control and Prevention. Principles of Community Engagement (1st ed.). Atlanta, GA: CDC/ATSDR Committee on Community Engagement; 1997.

## Slide 9
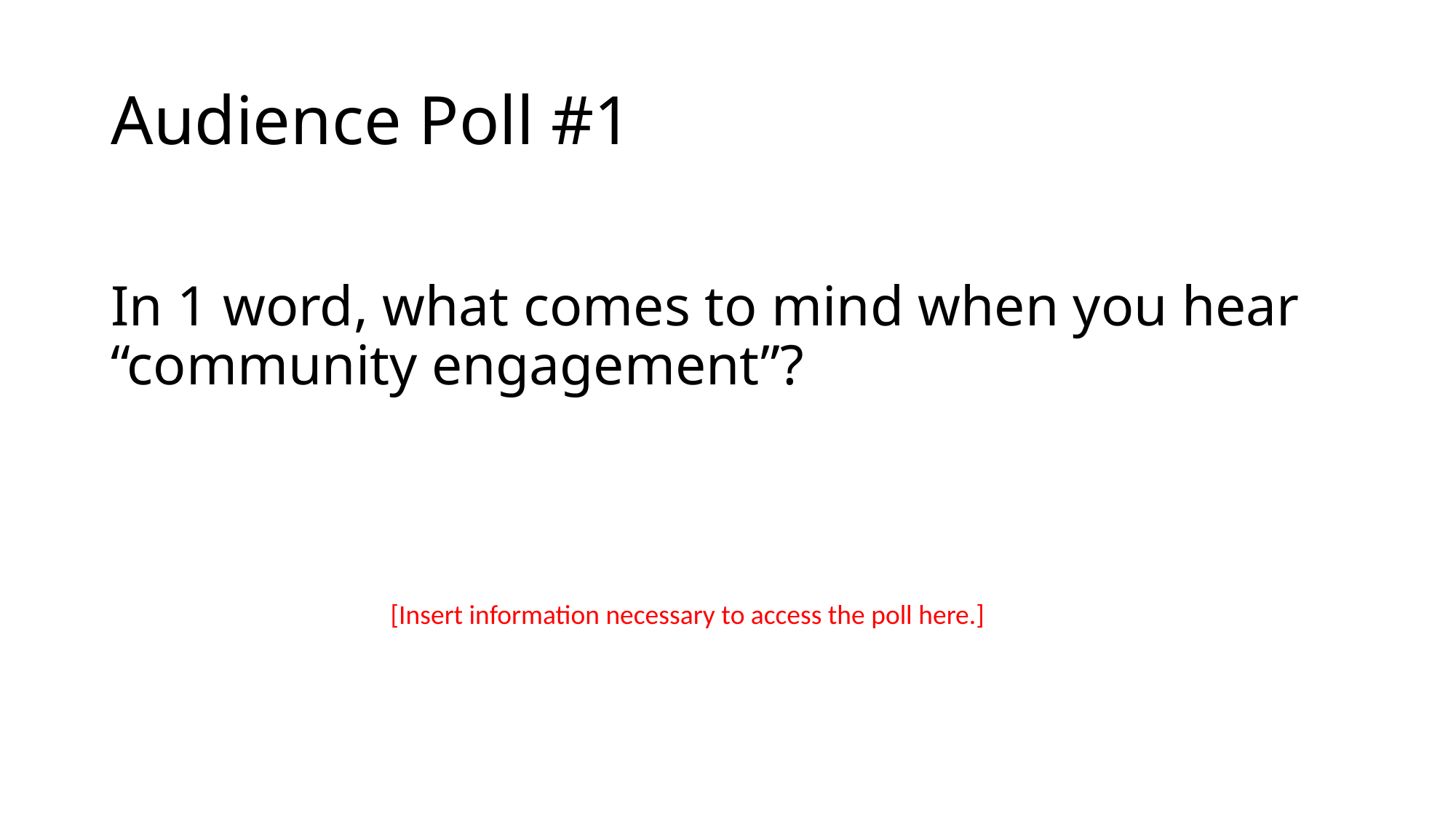

# Audience Poll #1
In 1 word, what comes to mind when you hear “community engagement”?
[Insert information necessary to access the poll here.]

## Slide 10
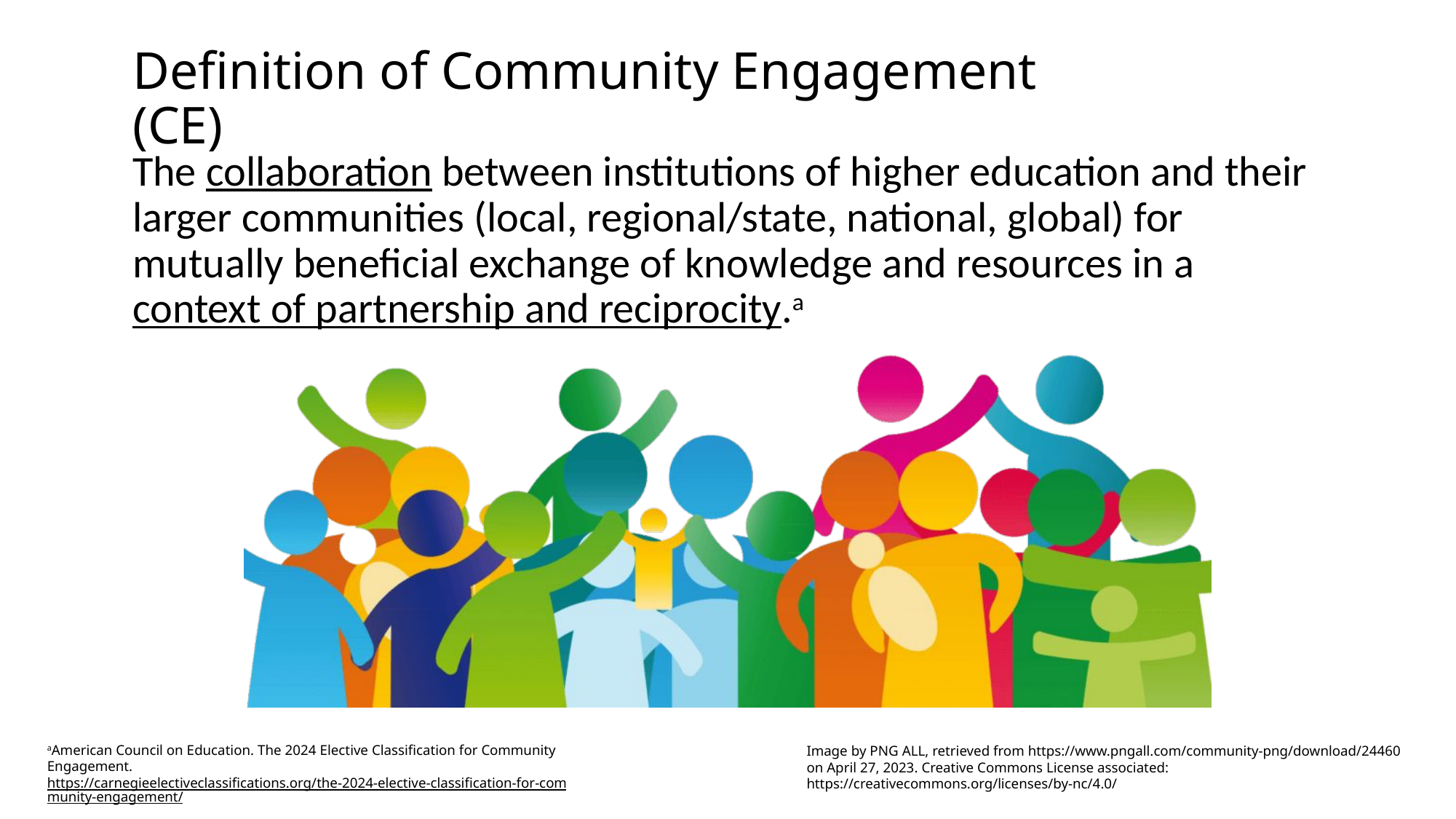

# Definition of Community Engagement (CE)
The collaboration between institutions of higher education and their larger communities (local, regional/state, national, global) for mutually beneficial exchange of knowledge and resources in a context of partnership and reciprocity.a
aAmerican Council on Education. The 2024 Elective Classification for Community Engagement. https://carnegieelectiveclassifications.org/the-2024-elective-classification-for-community-engagement/
Image by PNG ALL, retrieved from https://www.pngall.com/community-png/download/24460 on April 27, 2023. Creative Commons License associated: https://creativecommons.org/licenses/by-nc/4.0/

## Slide 11
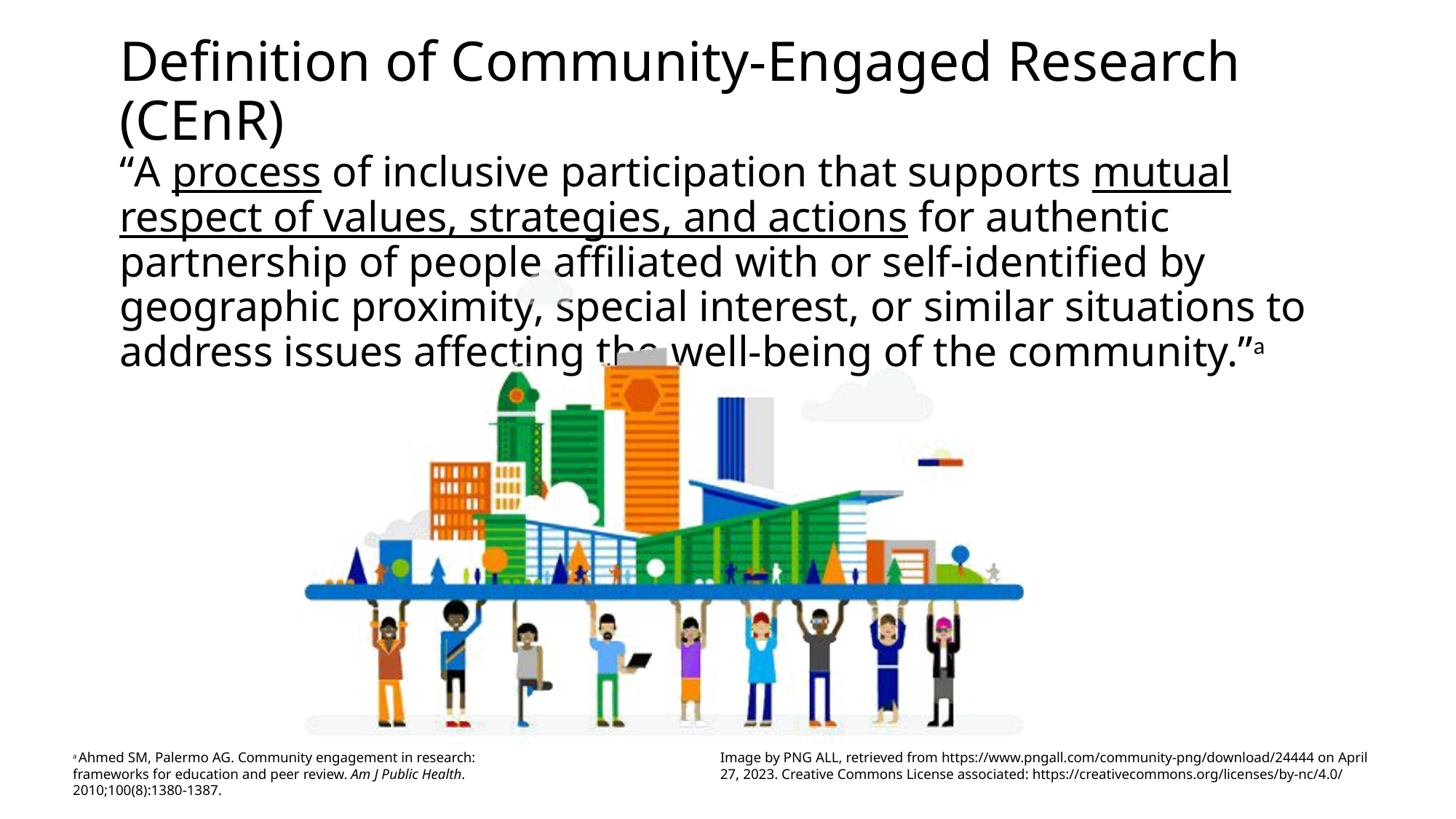

# Definition of Community-Engaged Research (CEnR)
“A process of inclusive participation that supports mutual respect of values, strategies, and actions for authentic partnership of people affiliated with or self-identified by geographic proximity, special interest, or similar situations to address issues affecting the well-being of the community.”a
a Ahmed SM, Palermo AG. Community engagement in research: frameworks for education and peer review. Am J Public Health. 2010;100(8):1380-1387.
Image by PNG ALL, retrieved from https://www.pngall.com/community-png/download/24444 on April 27, 2023. Creative Commons License associated: https://creativecommons.org/licenses/by-nc/4.0/

## Slide 12
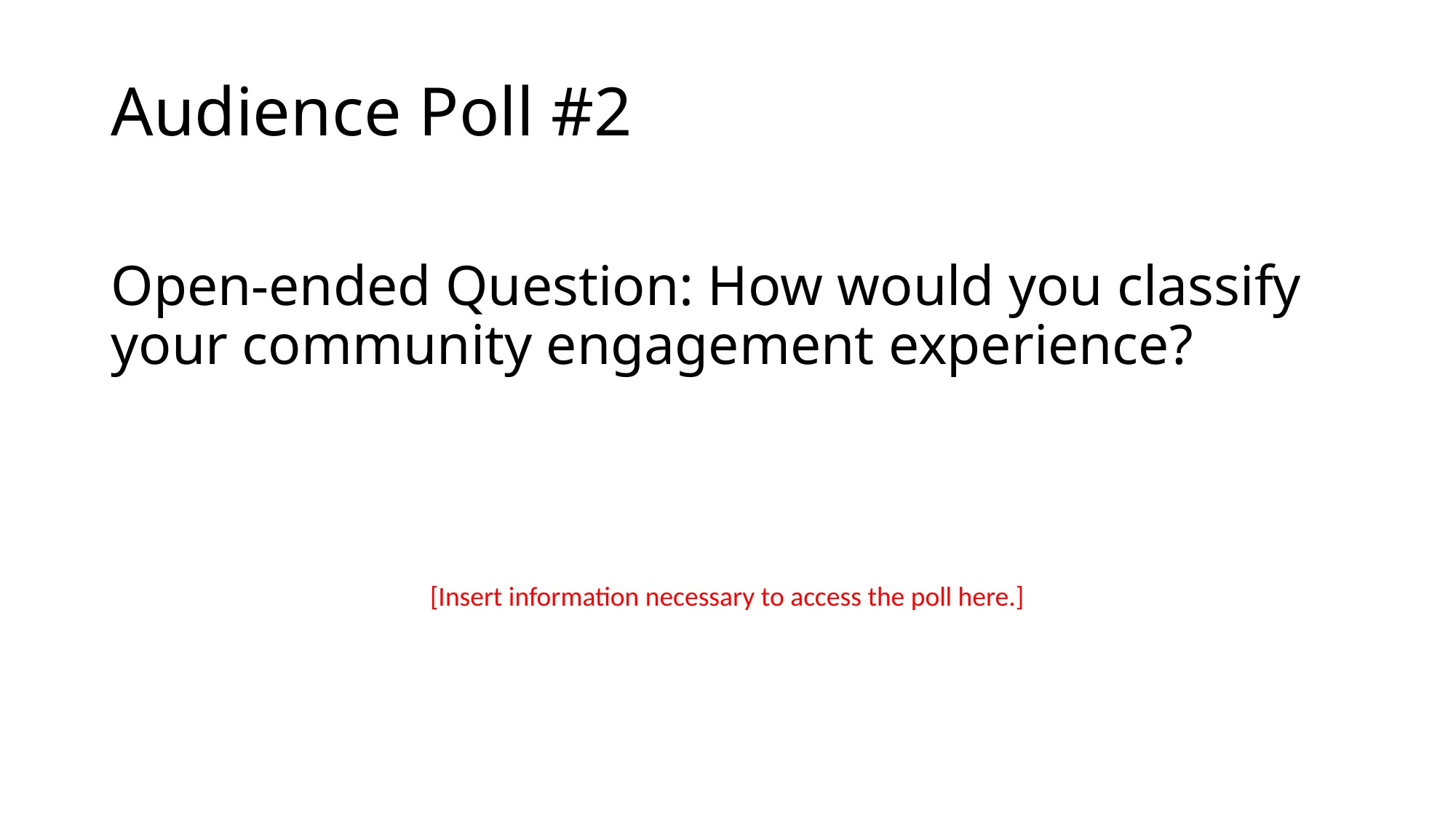

# Audience Poll #2
Open-ended Question: How would you classify your community engagement experience?
[Insert information necessary to access the poll here.]

## Slide 13
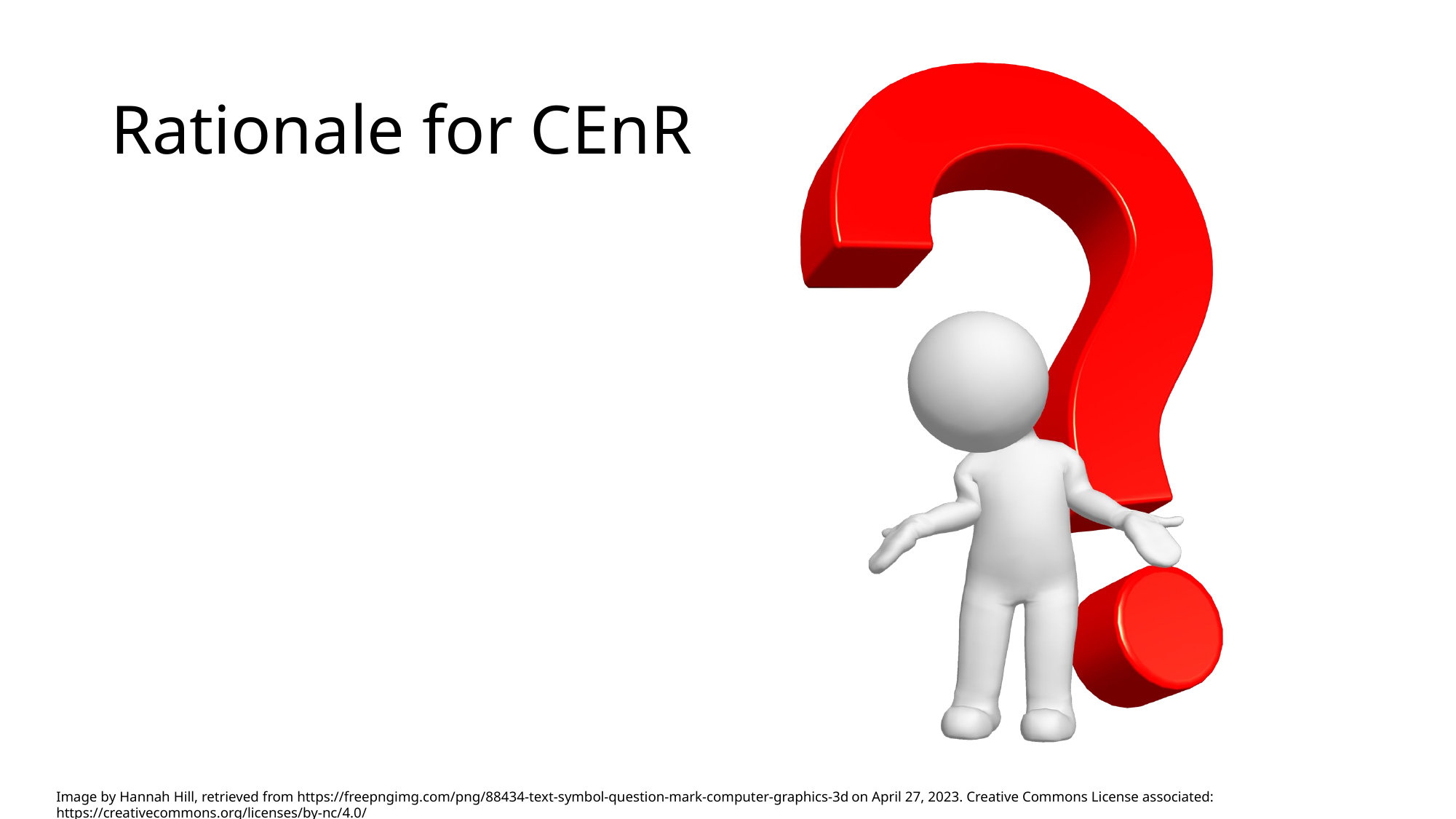

# Rationale for CEnR
Image by Hannah Hill, retrieved from https://freepngimg.com/png/88434-text-symbol-question-mark-computer-graphics-3d on April 27, 2023. Creative Commons License associated: https://creativecommons.org/licenses/by-nc/4.0/

## Slide 14
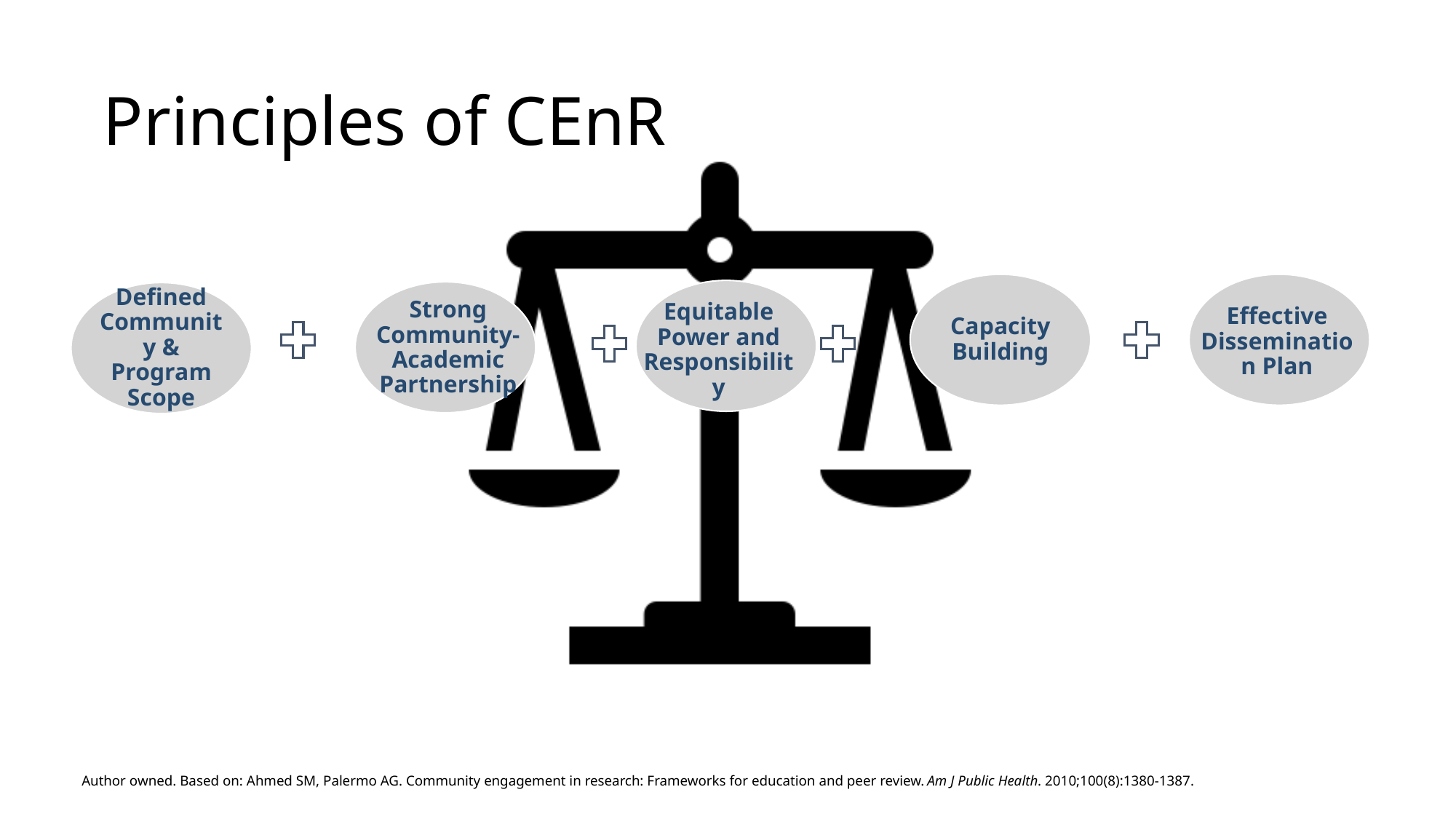

# Principles of CEnR
Capacity Building
Effective Dissemination Plan
Equitable Power and Responsibility
Strong Community-Academic Partnership
Defined Community & Program Scope
Author owned. Based on: Ahmed SM, Palermo AG. Community engagement in research: Frameworks for education and peer review. Am J Public Health. 2010;100(8):1380-1387.

## Slide 15
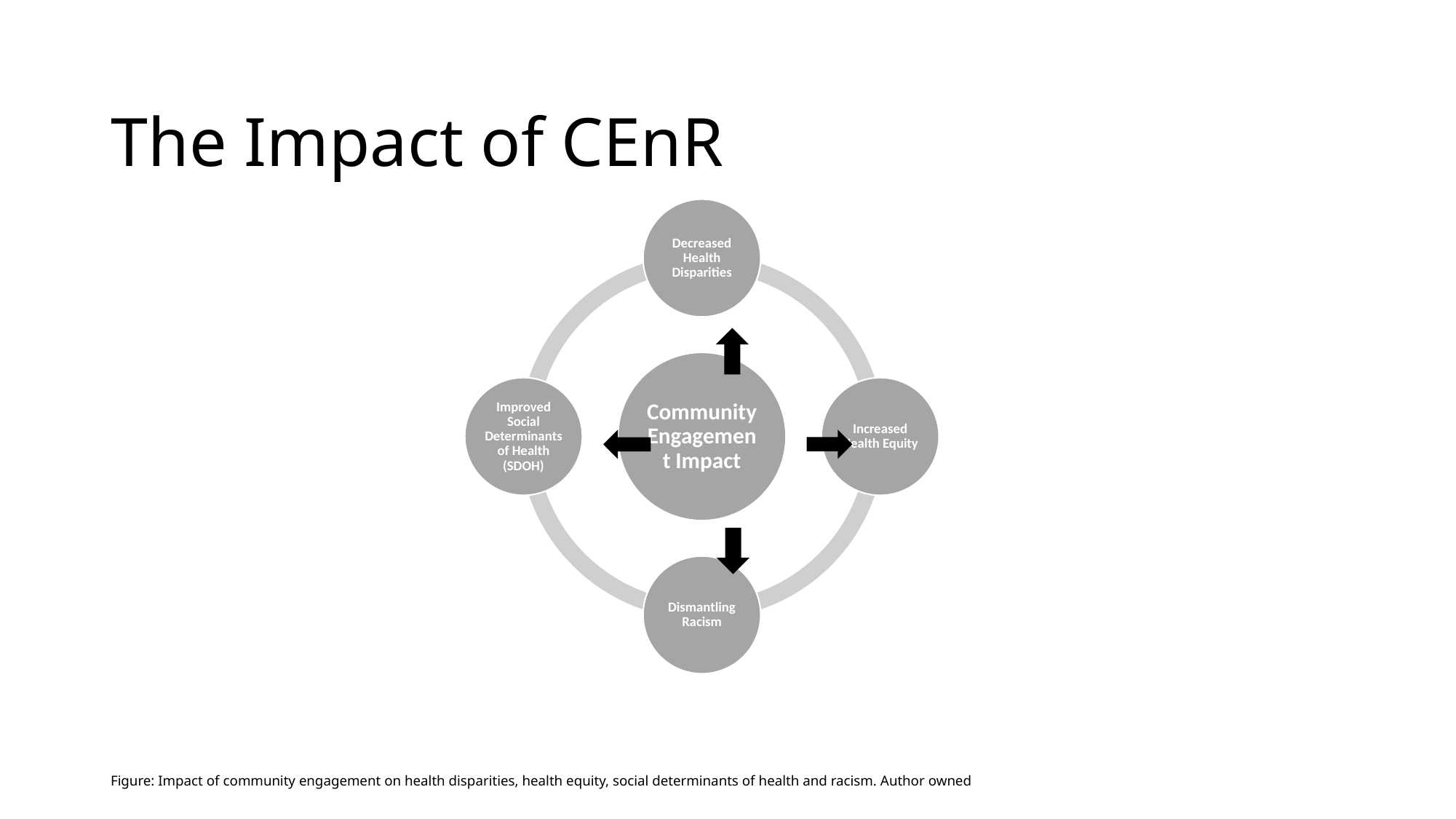

# The Impact of CEnR
Figure: Impact of community engagement on health disparities, health equity, social determinants of health and racism. Author owned

## Slide 16
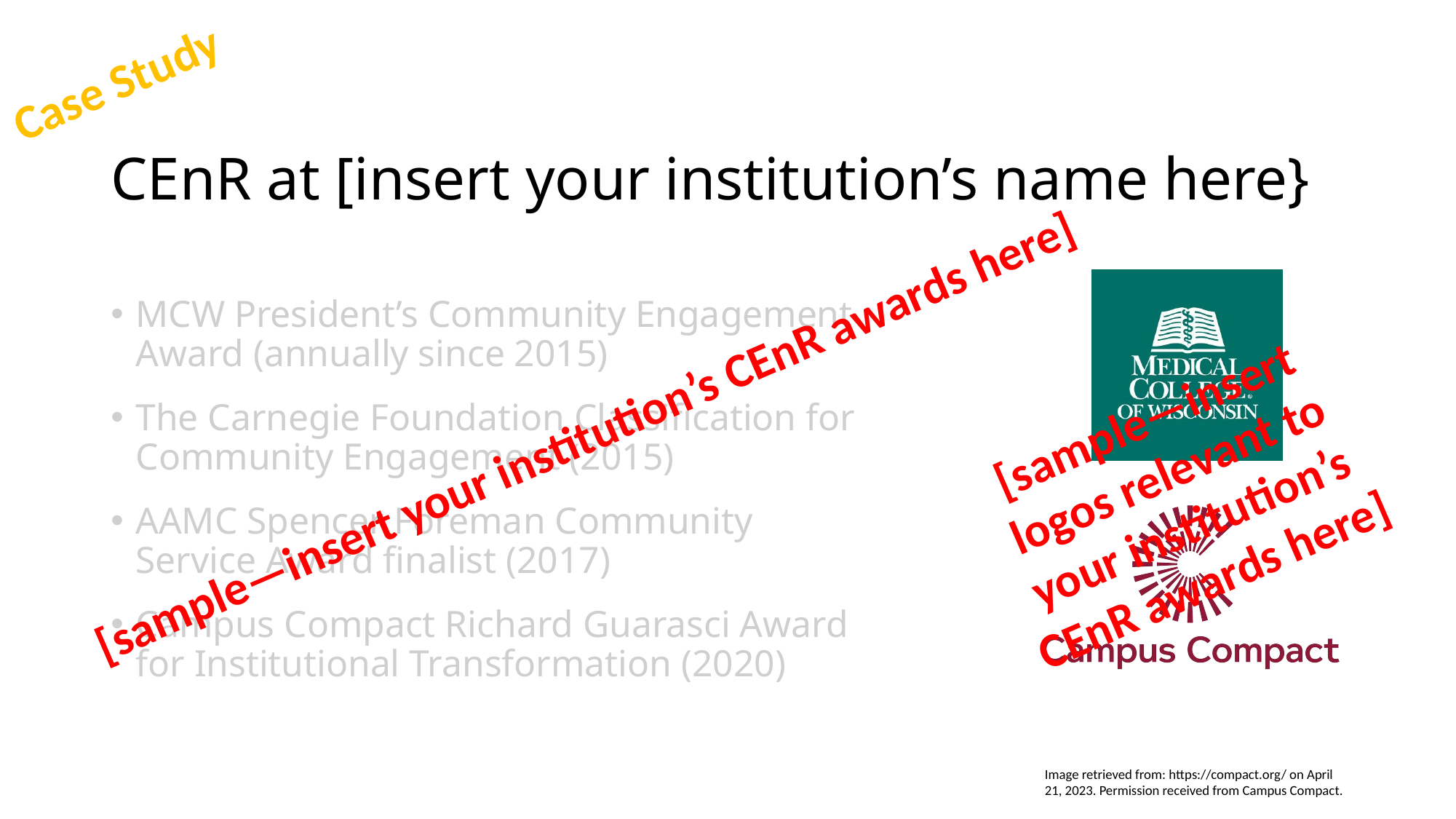

Case Study
# CEnR at [insert your institution’s name here}
MCW President’s Community Engagement Award (annually since 2015)
The Carnegie Foundation Classification for Community Engagement (2015)
AAMC Spencer Foreman Community Service Award finalist (2017)
Campus Compact Richard Guarasci Award for Institutional Transformation (2020)
[sample—insert logos relevant to your institution’s CEnR awards here]
[sample—insert your institution’s CEnR awards here]
Image retrieved from: https://compact.org/ on April 21, 2023. Permission received from Campus Compact.

## Slide 17
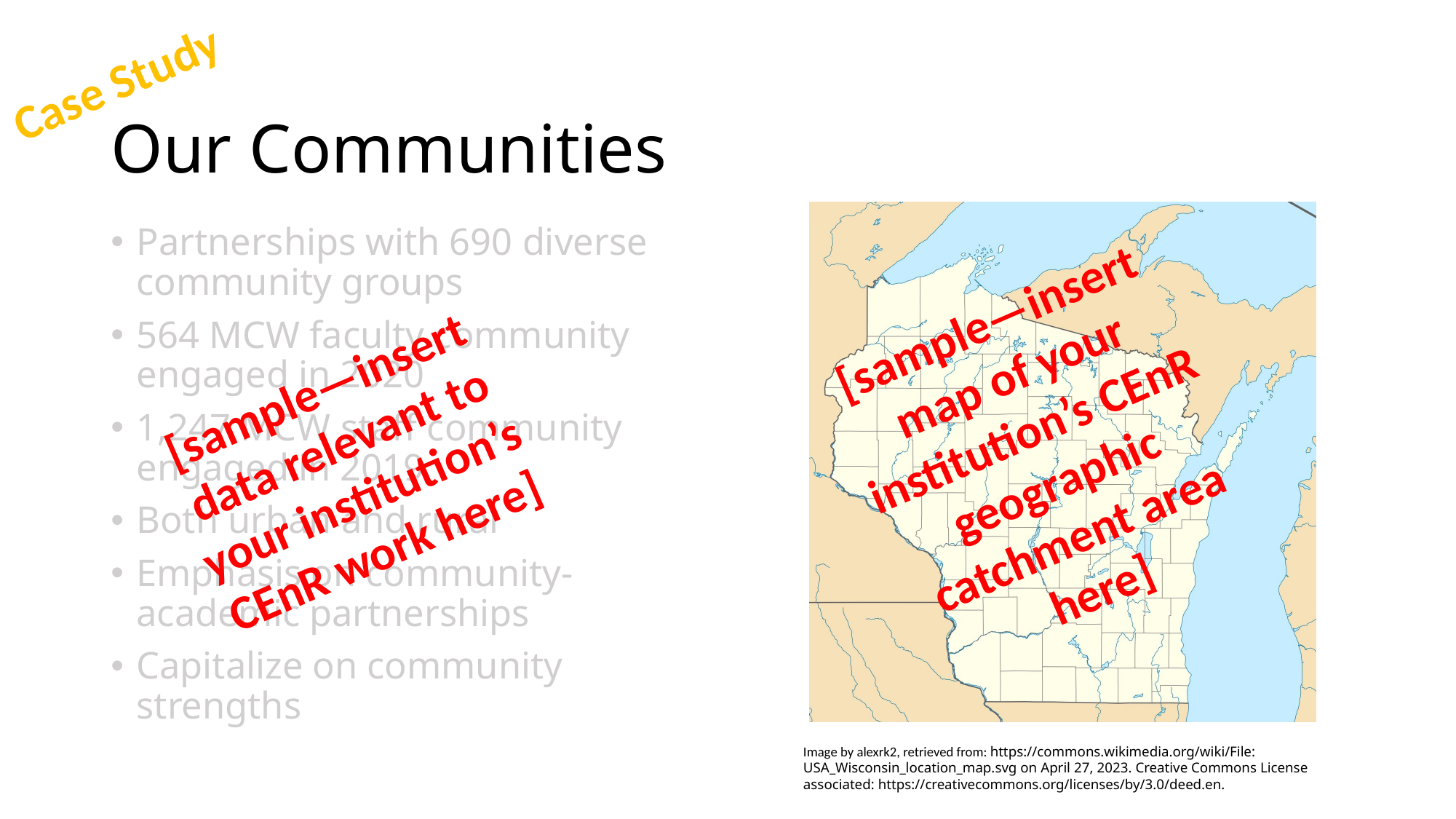

Case Study
# Our Communities
[sample—insert map of your institution’s CEnR geographic catchment area here]
Partnerships with 690 diverse community groups
564 MCW faculty community engaged in 2020
1,247 MCW staff community engaged in 2019
Both urban and rural
Emphasis on community-academic partnerships
Capitalize on community strengths
[sample—insert data relevant to your institution’s CEnR work here]
Image by alexrk2, retrieved from: https://commons.wikimedia.org/wiki/File:
USA_Wisconsin_location_map.svg on April 27, 2023. Creative Commons License associated: https://creativecommons.org/licenses/by/3.0/deed.en.

## Slide 18
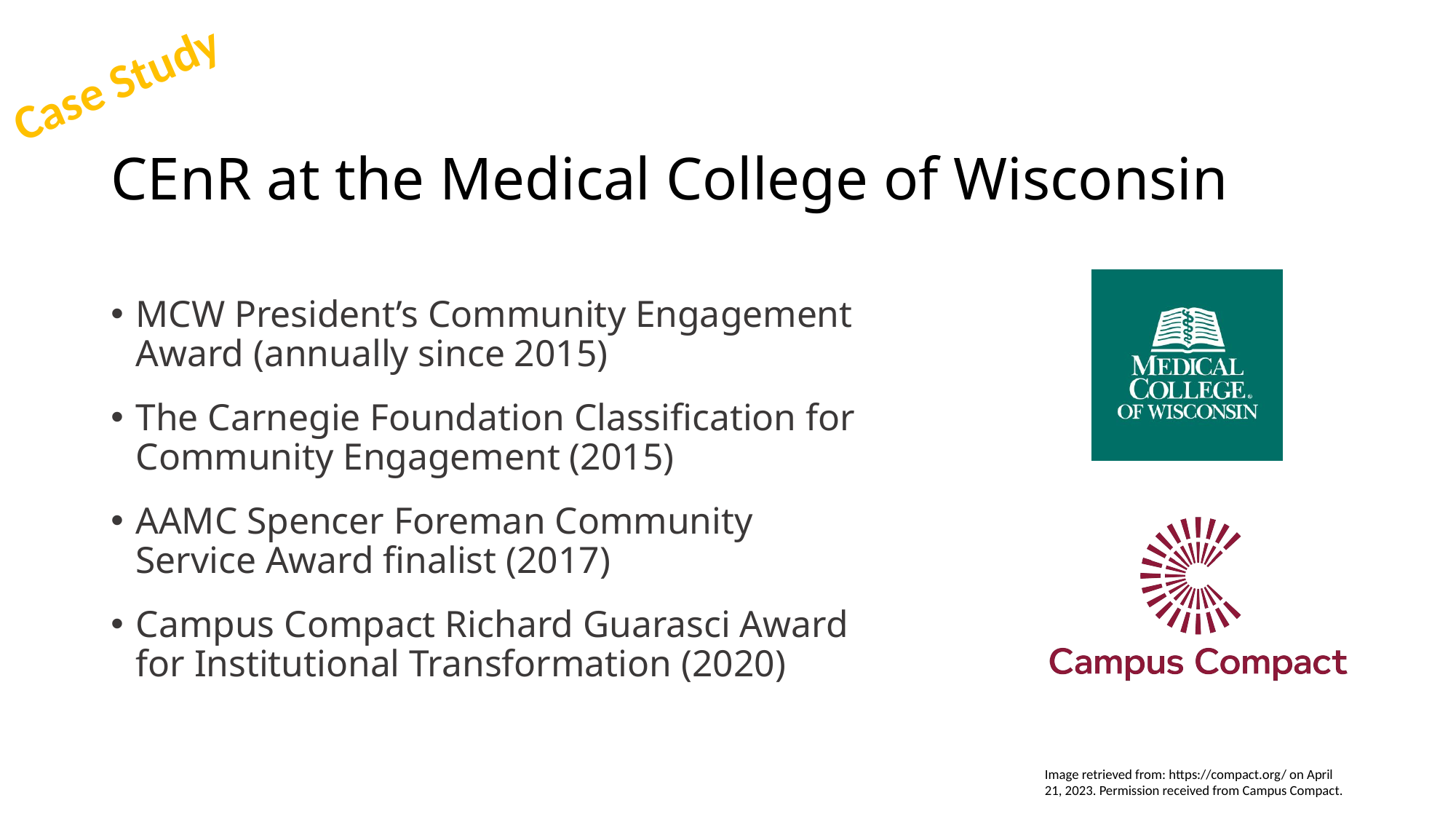

Case Study
# CEnR at the Medical College of Wisconsin
MCW President’s Community Engagement Award (annually since 2015)
The Carnegie Foundation Classification for Community Engagement (2015)
AAMC Spencer Foreman Community Service Award finalist (2017)
Campus Compact Richard Guarasci Award for Institutional Transformation (2020)
Image retrieved from: https://compact.org/ on April 21, 2023. Permission received from Campus Compact.

## Slide 19
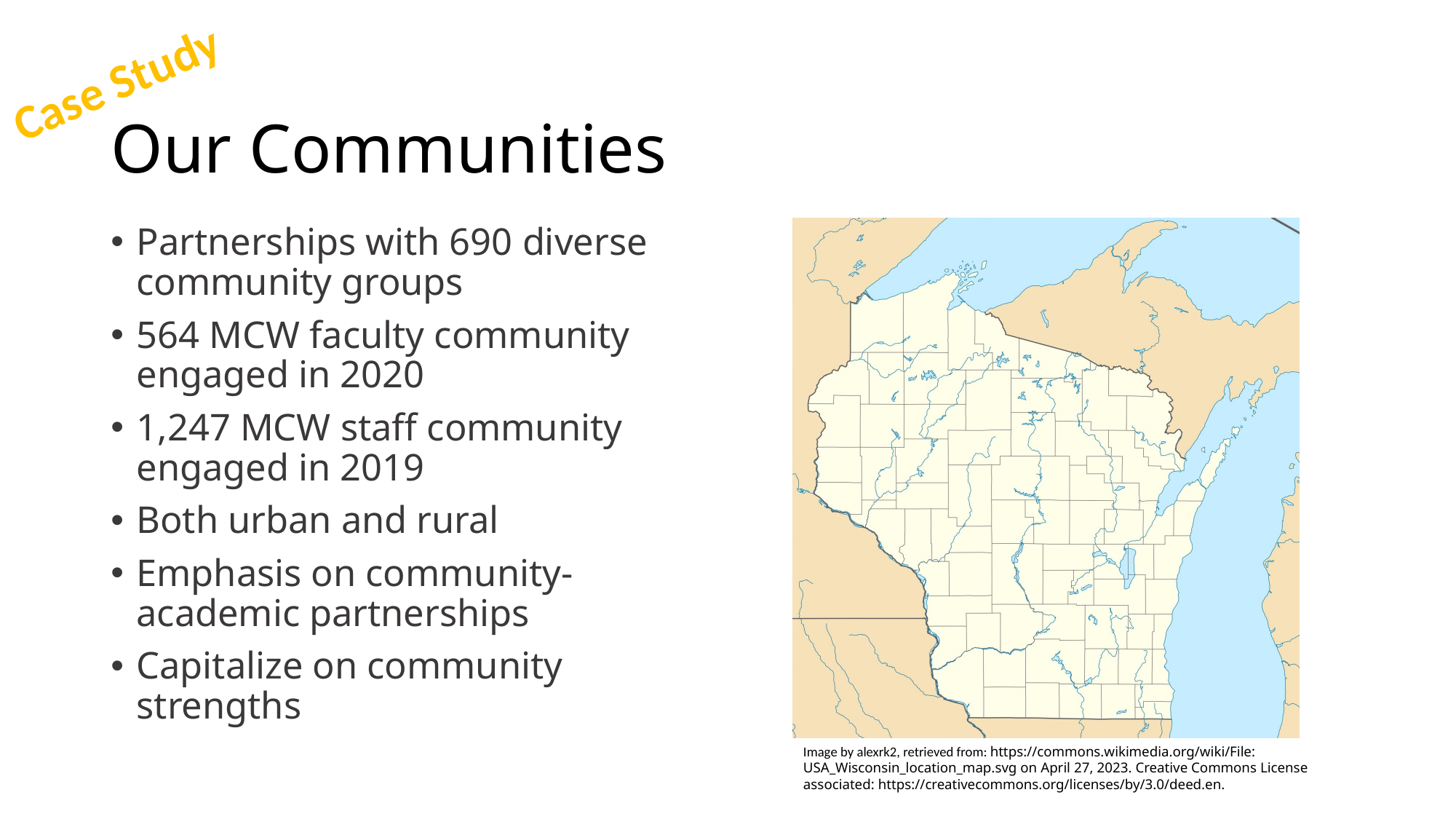

Case Study
# Our Communities
Partnerships with 690 diverse community groups
564 MCW faculty community engaged in 2020
1,247 MCW staff community engaged in 2019
Both urban and rural
Emphasis on community-academic partnerships
Capitalize on community strengths
Image by alexrk2, retrieved from: https://commons.wikimedia.org/wiki/File:
USA_Wisconsin_location_map.svg on April 27, 2023. Creative Commons License associated: https://creativecommons.org/licenses/by/3.0/deed.en.

## Slide 20
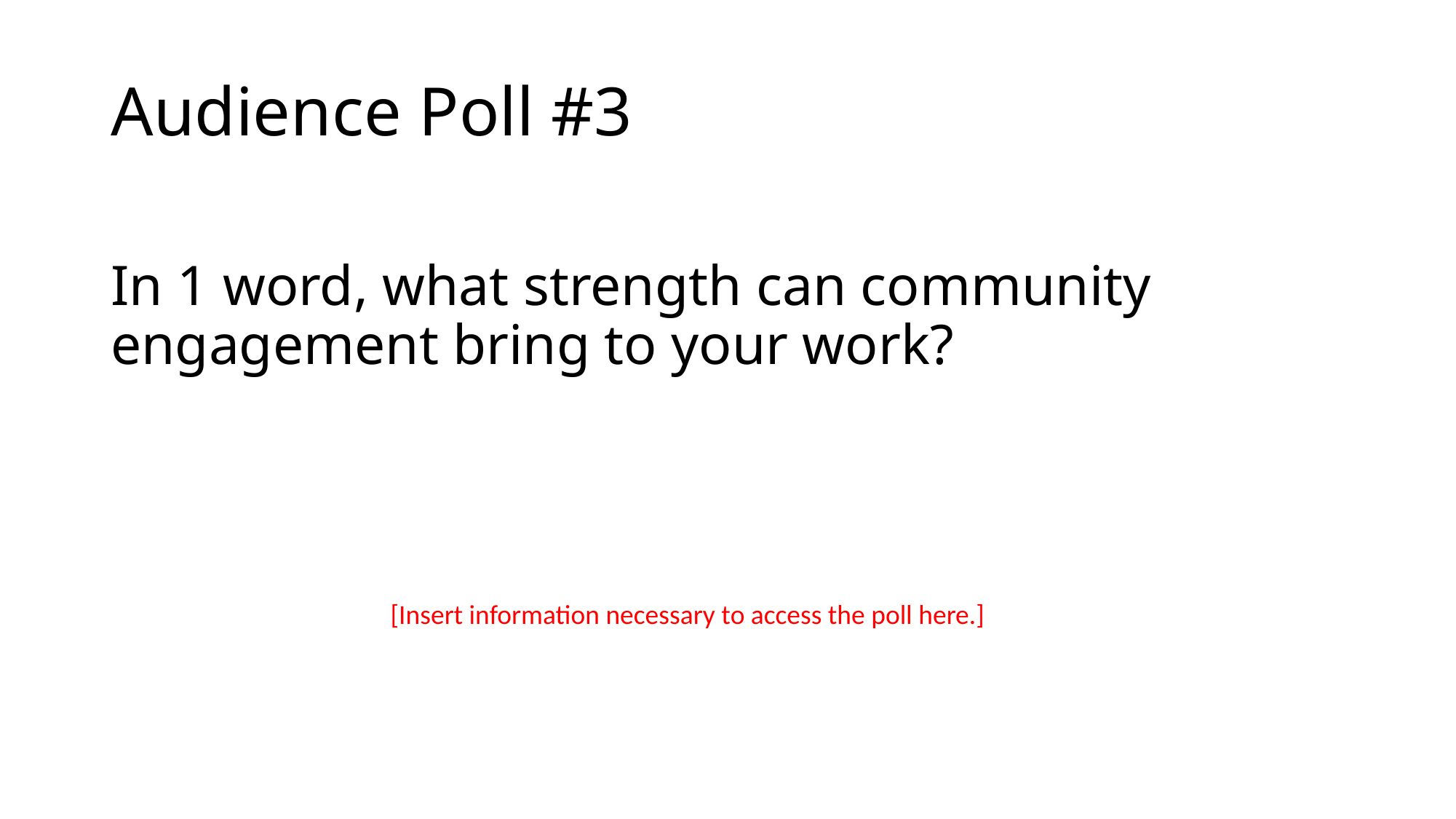

# Audience Poll #3
In 1 word, what strength can community engagement bring to your work?
[Insert information necessary to access the poll here.]

## Slide 21
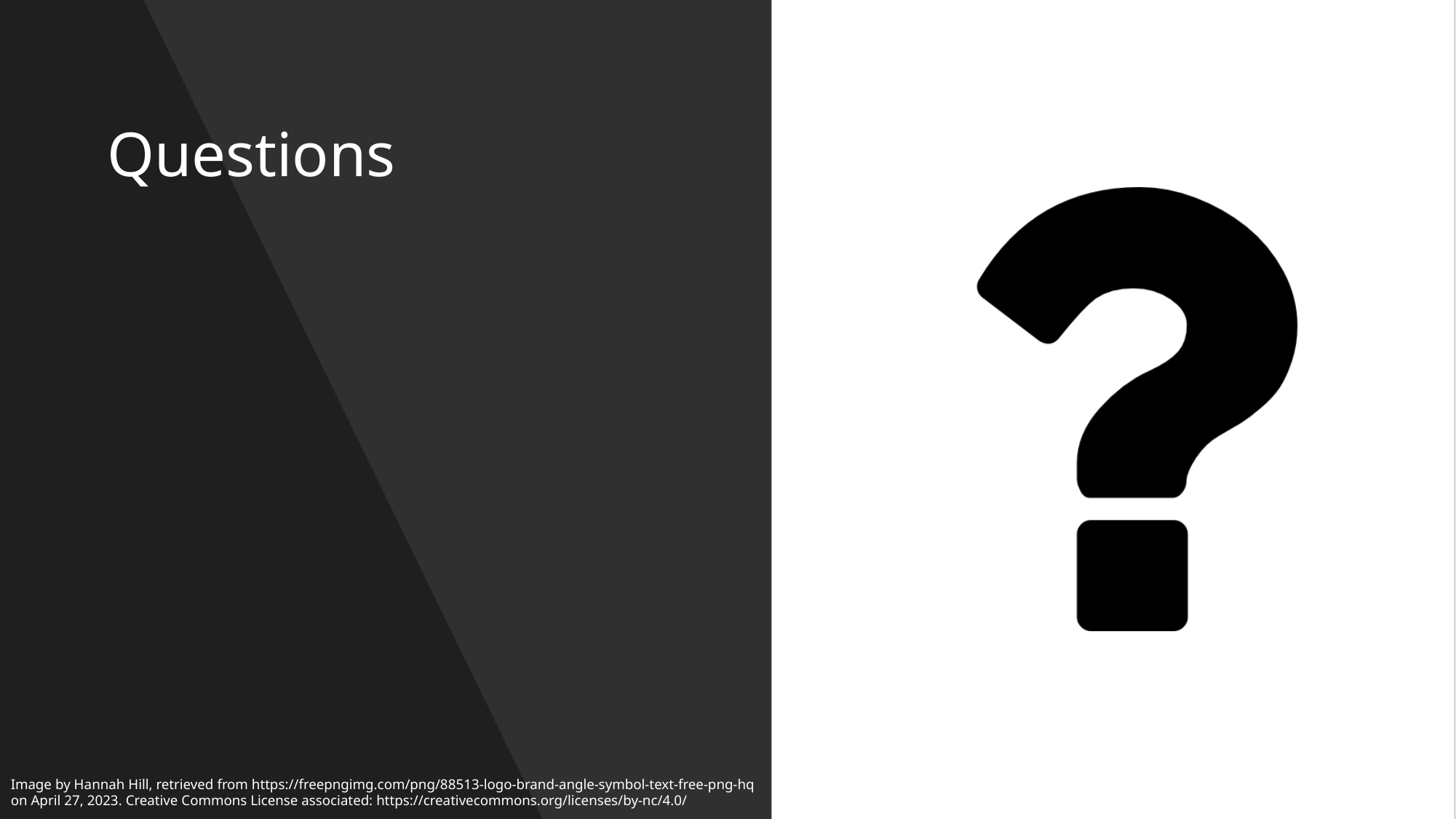

# Questions
Image by Hannah Hill, retrieved from https://freepngimg.com/png/88513-logo-brand-angle-symbol-text-free-png-hq on April 27, 2023. Creative Commons License associated: https://creativecommons.org/licenses/by-nc/4.0/

## Slide 22
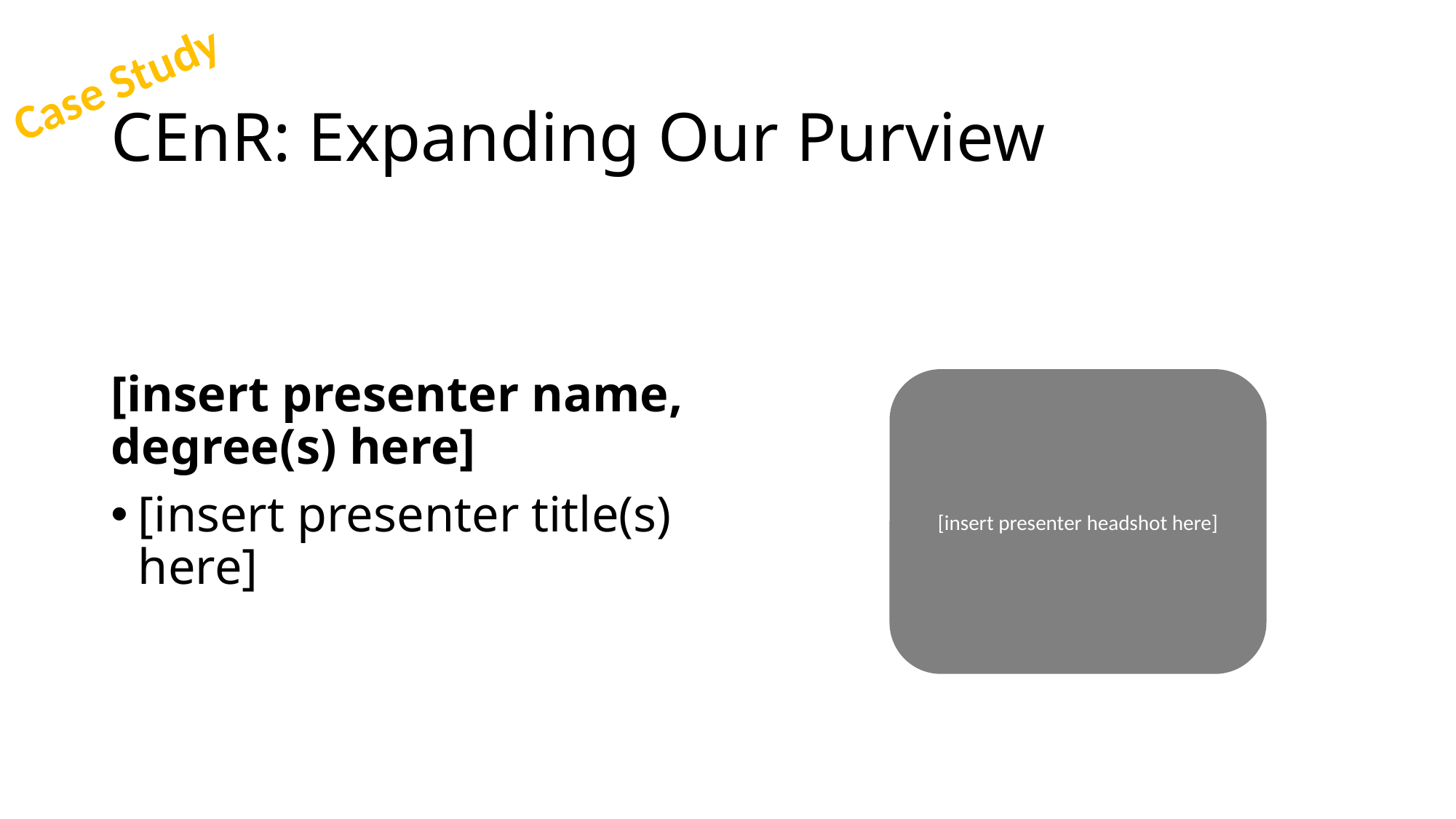

Case Study
# CEnR: Expanding Our Purview
[insert presenter name, degree(s) here]
[insert presenter title(s) here]
[insert presenter headshot here]

## Slide 23
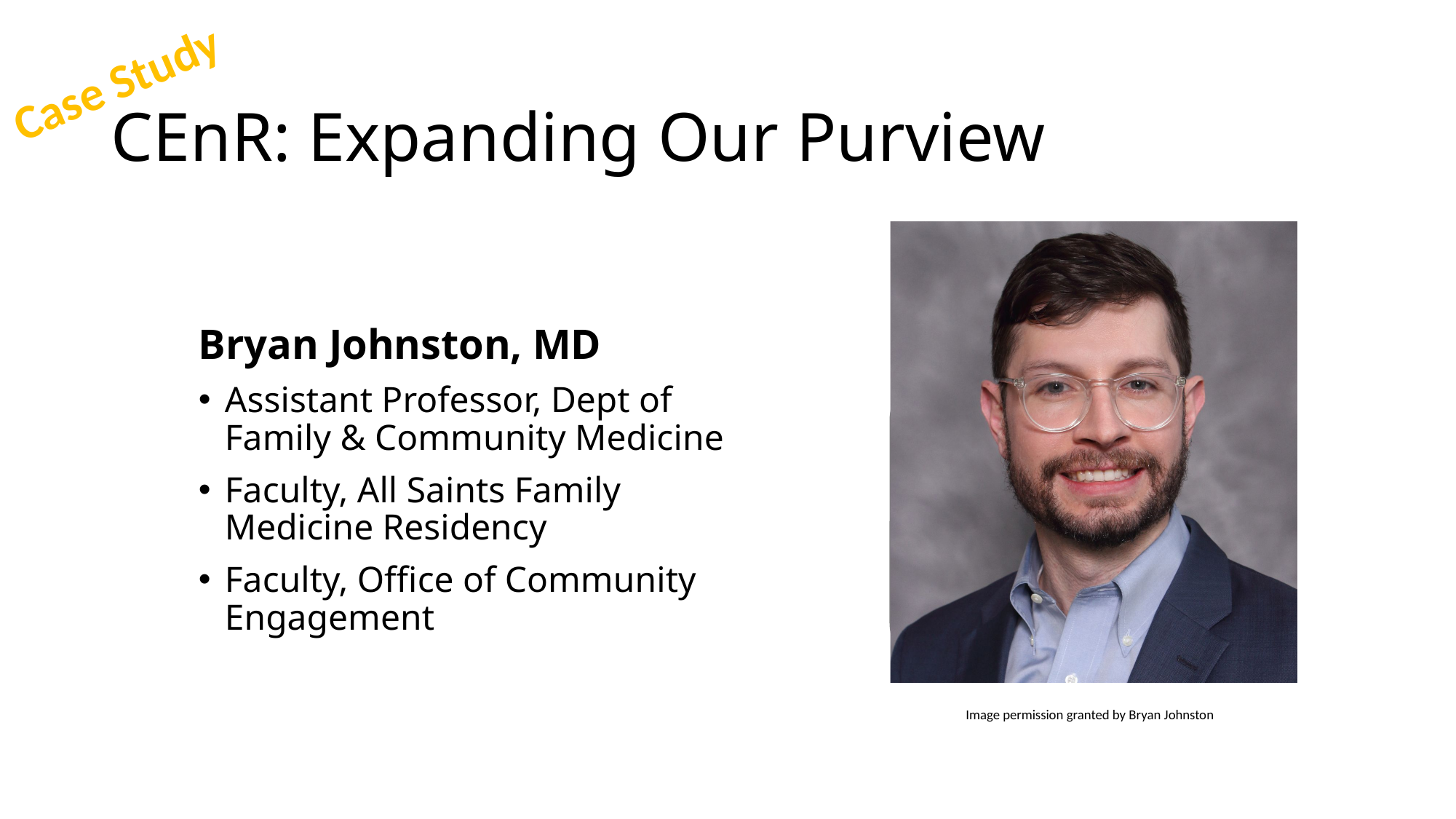

Case Study
# CEnR: Expanding Our Purview
Bryan Johnston, MD
Assistant Professor, Dept of Family & Community Medicine
Faculty, All Saints Family Medicine Residency
Faculty, Office of Community Engagement
[insert presenter headshot here]
Image permission granted by Bryan Johnston

## Slide 24
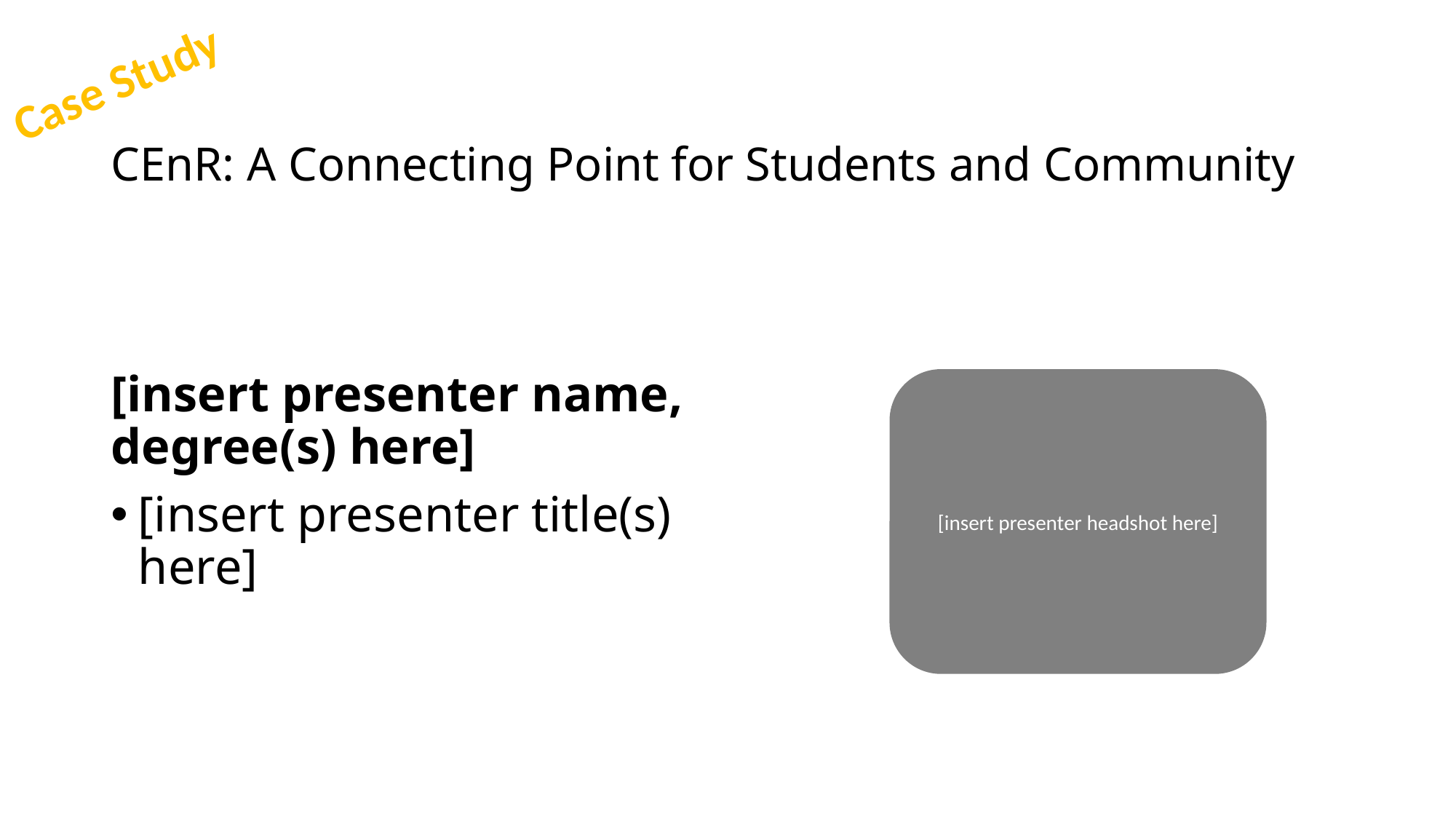

Case Study
# CEnR: A Connecting Point for Students and Community
[insert presenter name, degree(s) here]
[insert presenter title(s) here]
[insert presenter headshot here]

## Slide 25
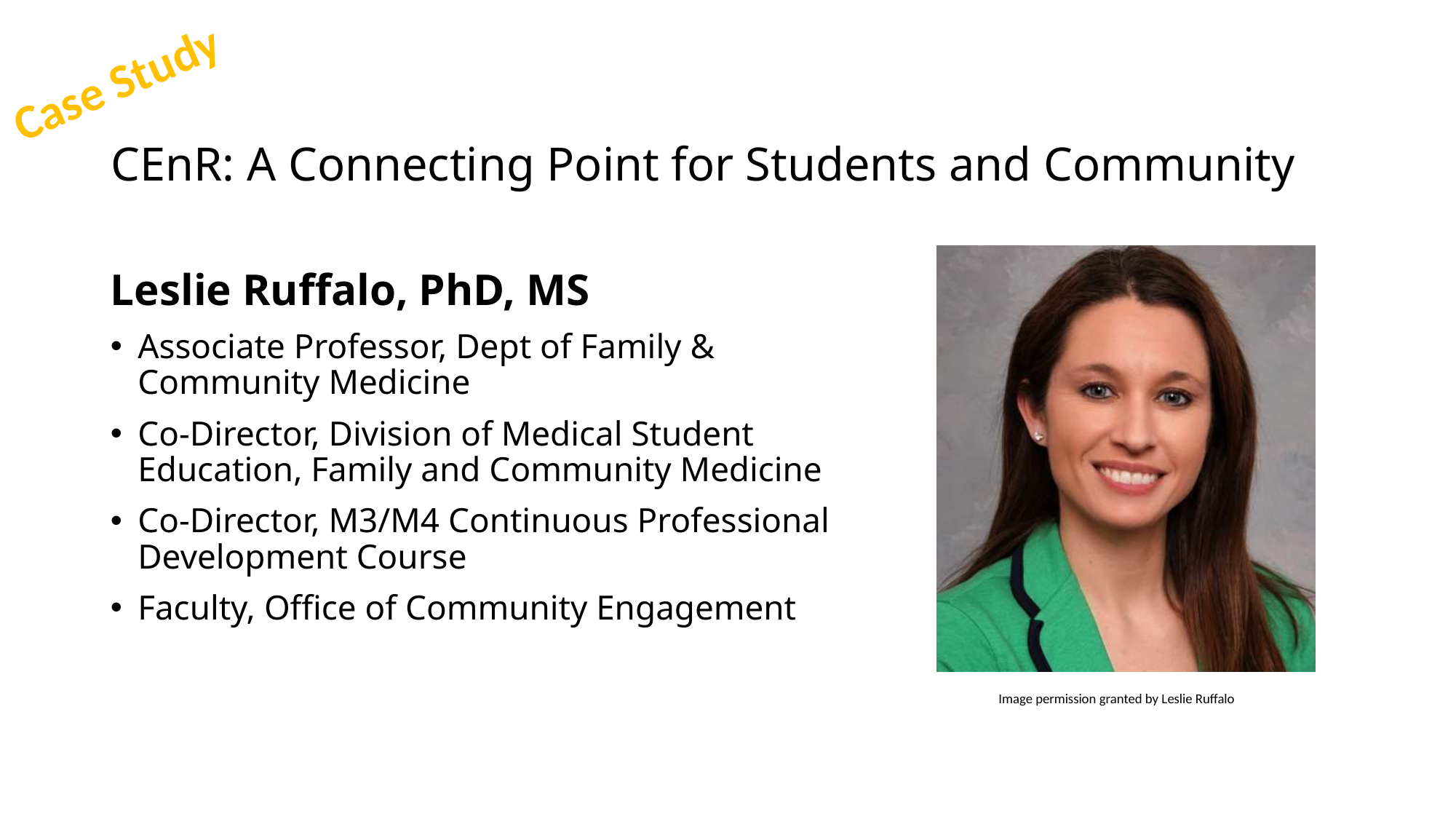

Case Study
# CEnR: A Connecting Point for Students and Community
Leslie Ruffalo, PhD, MS
Associate Professor, Dept of Family & Community Medicine
Co-Director, Division of Medical Student Education, Family and Community Medicine
Co-Director, M3/M4 Continuous Professional Development Course
Faculty, Office of Community Engagement
Image permission granted by Leslie Ruffalo

## Slide 26
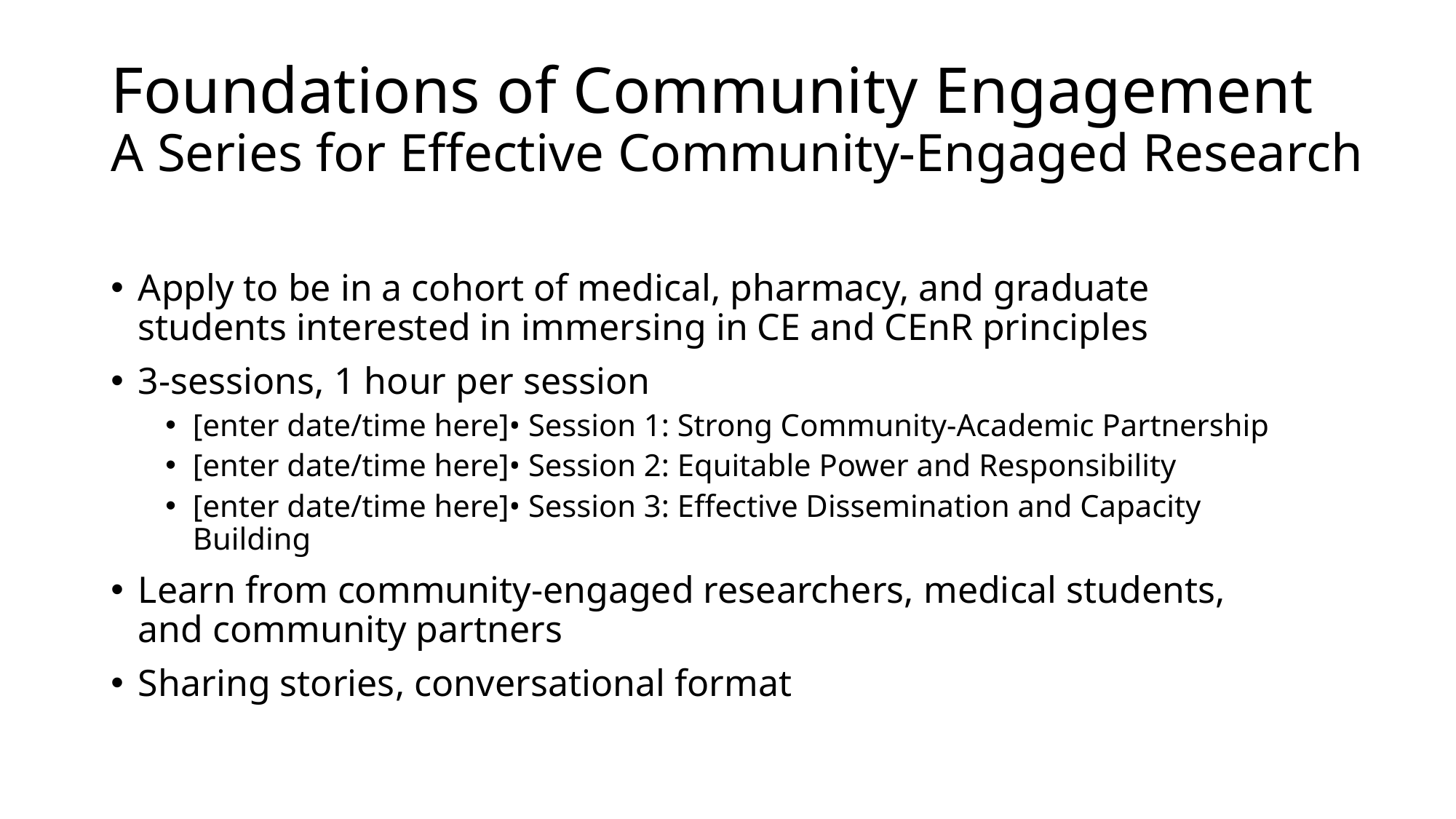

# Foundations of Community Engagement A Series for Effective Community-Engaged Research
Apply to be in a cohort of medical, pharmacy, and graduate students interested in immersing in CE and CEnR principles
3-sessions, 1 hour per session
[enter date/time here]• Session 1: Strong Community-Academic Partnership
[enter date/time here]• Session 2: Equitable Power and Responsibility
[enter date/time here]• Session 3: Effective Dissemination and Capacity Building
Learn from community-engaged researchers, medical students, and community partners
Sharing stories, conversational format

## Slide 27
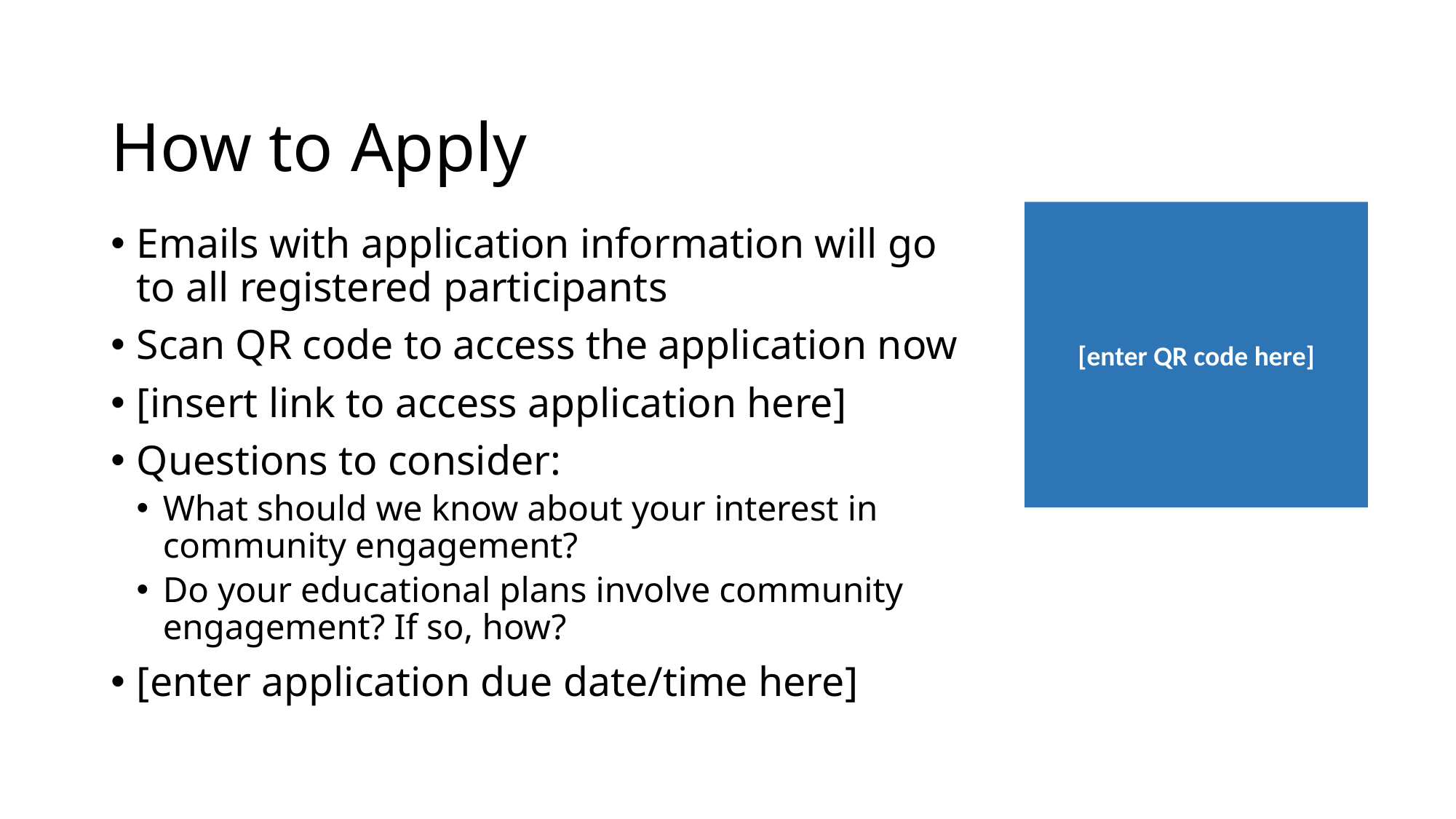

# How to Apply
[enter QR code here]
Emails with application information will go to all registered participants
Scan QR code to access the application now
[insert link to access application here]
Questions to consider:
What should we know about your interest in community engagement?
Do your educational plans involve community engagement? If so, how?
[enter application due date/time here]

## Slide 28
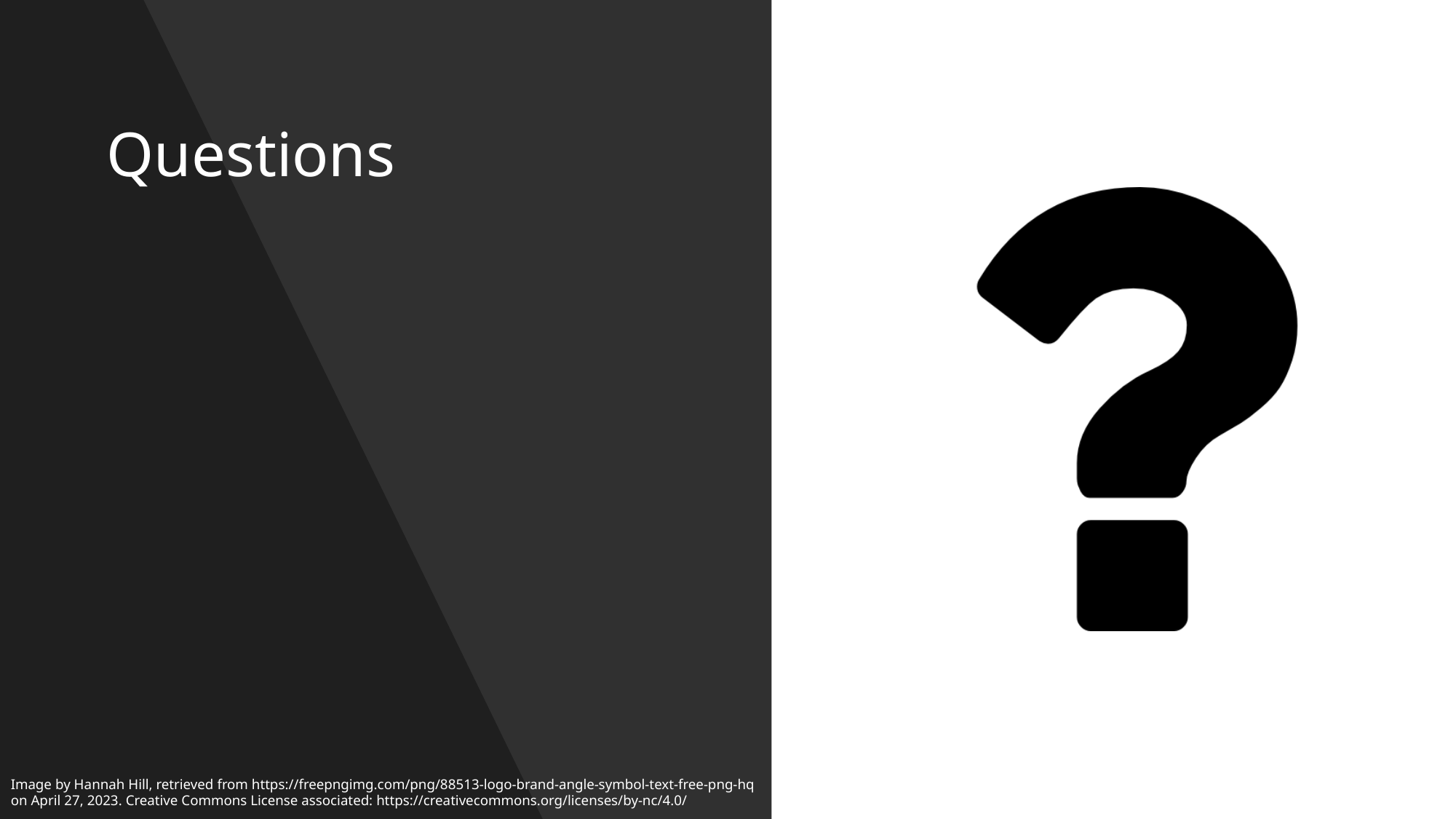

Questions
Image by Hannah Hill, retrieved from https://freepngimg.com/png/88513-logo-brand-angle-symbol-text-free-png-hq on April 27, 2023. Creative Commons License associated: https://creativecommons.org/licenses/by-nc/4.0/

## Slide 29
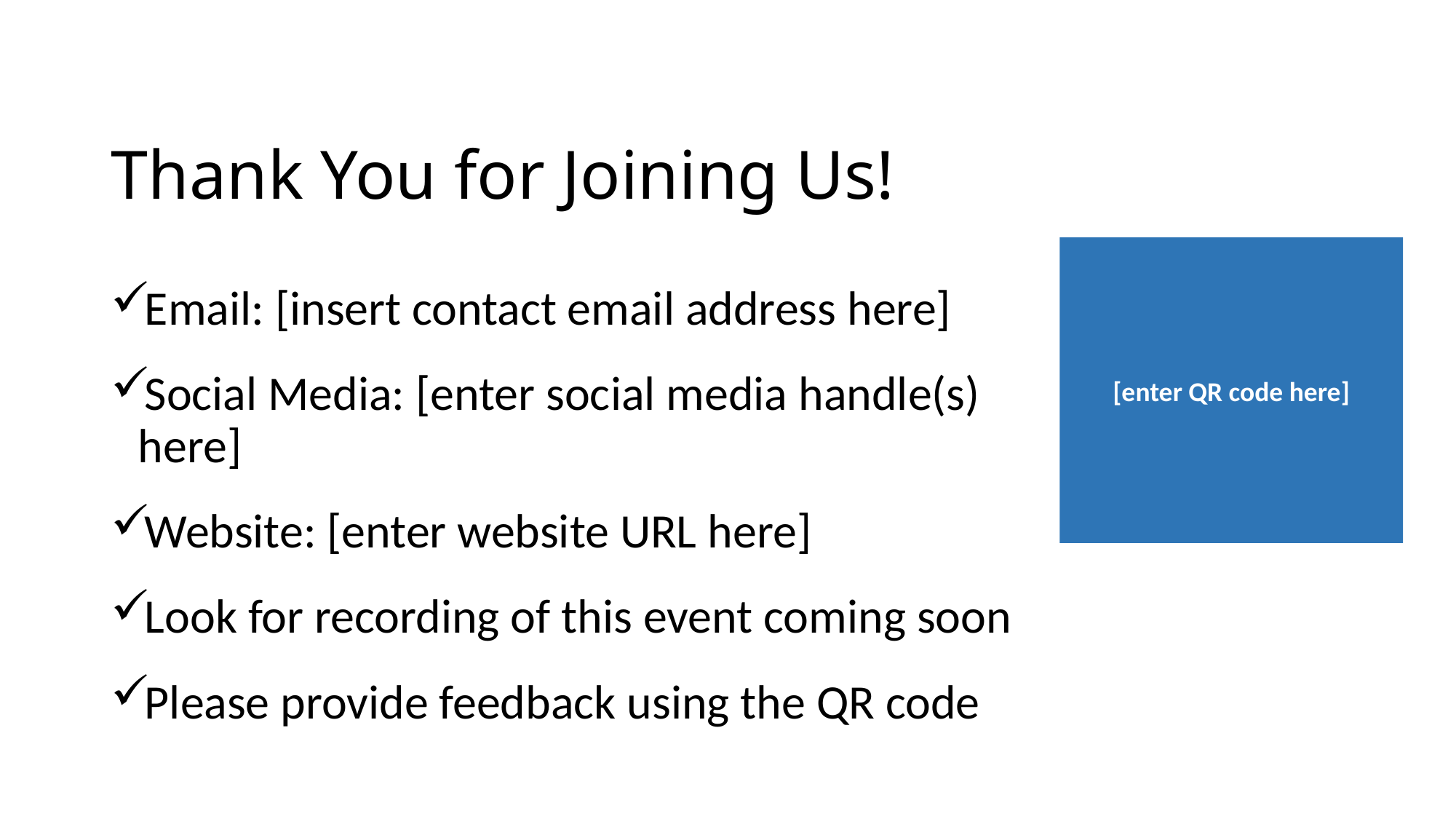

# Thank You for Joining Us!
[enter QR code here]
Email: [insert contact email address here]
Social Media: [enter social media handle(s) here]
Website: [enter website URL here]
Look for recording of this event coming soon
Please provide feedback using the QR code

## Slide 30
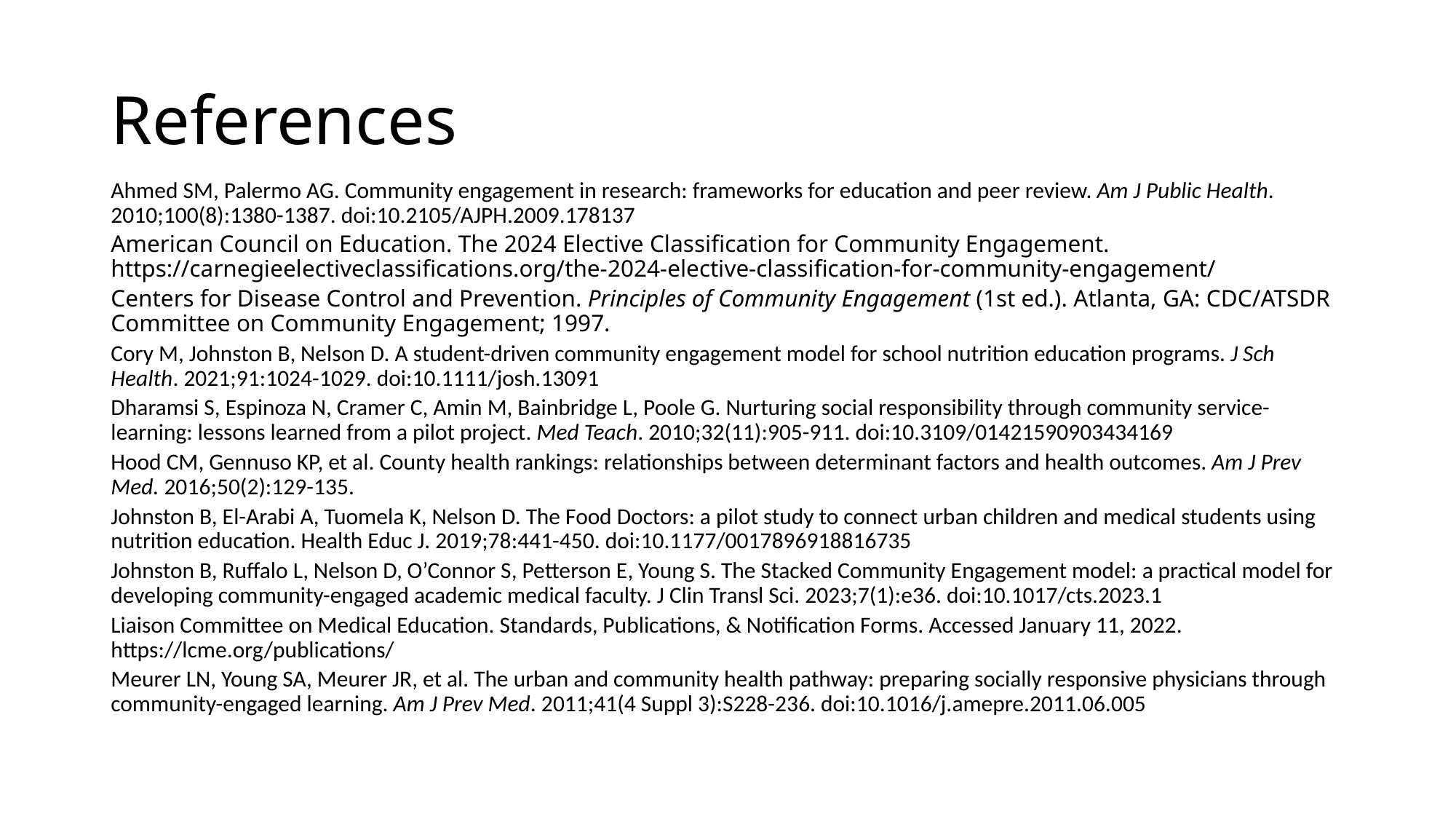

# References
Ahmed SM, Palermo AG. Community engagement in research: frameworks for education and peer review. Am J Public Health. 2010;100(8):1380-1387. doi:10.2105/AJPH.2009.178137
American Council on Education. The 2024 Elective Classification for Community Engagement. https://carnegieelectiveclassifications.org/the-2024-elective-classification-for-community-engagement/
Centers for Disease Control and Prevention. Principles of Community Engagement (1st ed.). Atlanta, GA: CDC/ATSDR Committee on Community Engagement; 1997.
Cory M, Johnston B, Nelson D. A student-driven community engagement model for school nutrition education programs. J Sch Health. 2021;91:1024-1029. doi:10.1111/josh.13091
Dharamsi S, Espinoza N, Cramer C, Amin M, Bainbridge L, Poole G. Nurturing social responsibility through community service-learning: lessons learned from a pilot project. Med Teach. 2010;32(11):905-911. doi:10.3109/01421590903434169
Hood CM, Gennuso KP, et al. County health rankings: relationships between determinant factors and health outcomes. Am J Prev Med. 2016;50(2):129-135.
Johnston B, El-Arabi A, Tuomela K, Nelson D. The Food Doctors: a pilot study to connect urban children and medical students using nutrition education. Health Educ J. 2019;78:441-450. doi:10.1177/0017896918816735
Johnston B, Ruffalo L, Nelson D, O’Connor S, Petterson E, Young S. The Stacked Community Engagement model: a practical model for developing community-engaged academic medical faculty. J Clin Transl Sci. 2023;7(1):e36. doi:10.1017/cts.2023.1
Liaison Committee on Medical Education. Standards, Publications, & Notification Forms. Accessed January 11, 2022. https://lcme.org/publications/
Meurer LN, Young SA, Meurer JR, et al. The urban and community health pathway: preparing socially responsive physicians through community-engaged learning. Am J Prev Med. 2011;41(4 Suppl 3):S228-236. doi:10.1016/j.amepre.2011.06.005
