# Supplementary material for: Foundations of Community Engagement: A Series for Effective Community-Engaged Research
Source: MedEdPORTAL. 2023 Oct 10;19:11350. doi: 10.15766/mep_2374-8265.11350 (PMC10562524; doi:10.15766/mep_2374-8265.11350)
Supplement: Supplementary file 1 — CE Didactic Session Slides.pptxApplication for Small-Group Series.docxCommunity-Academic Partnership Slides.pptxEquitable Power and Responsibility Slides.pptxEquitable Power and Responsibility Case Studies.docxCapacity Building and Dissemination Slides.pptxFacilitator Guide.docxCE Didactic Session Evaluation.docxSmall-Group Session Evaluation.docx [file mep_2374-8265.11350-s001.zip › C. Community-Academic Partnership Slides.pptx]

## Slide 1
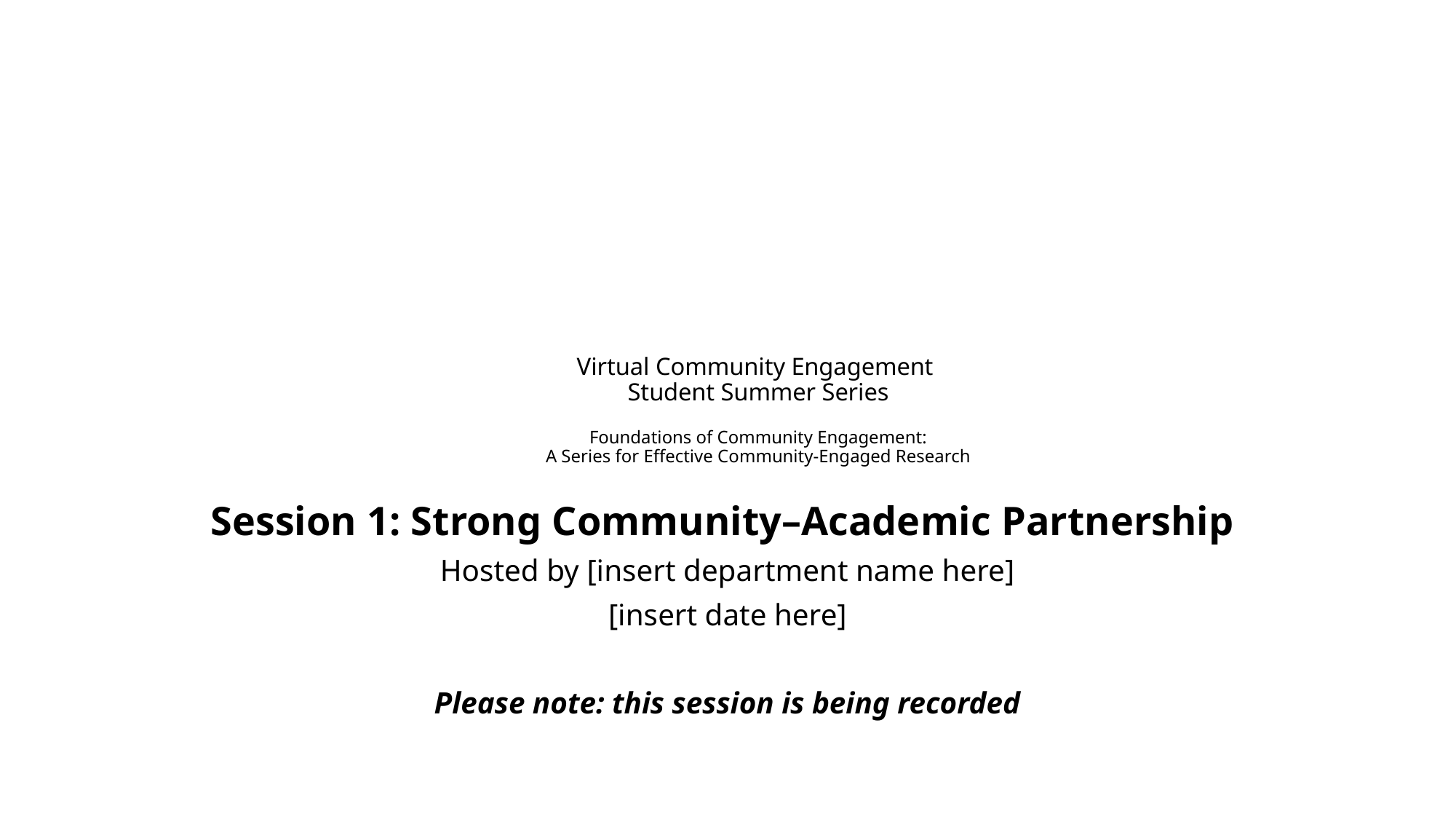

# Virtual Community Engagement Student Summer Series Foundations of Community Engagement: A Series for Effective Community-Engaged Research
Session 1: Strong Community–Academic Partnership
Hosted by [insert department name here]
[insert date here]
Please note: this session is being recorded

## Slide 2
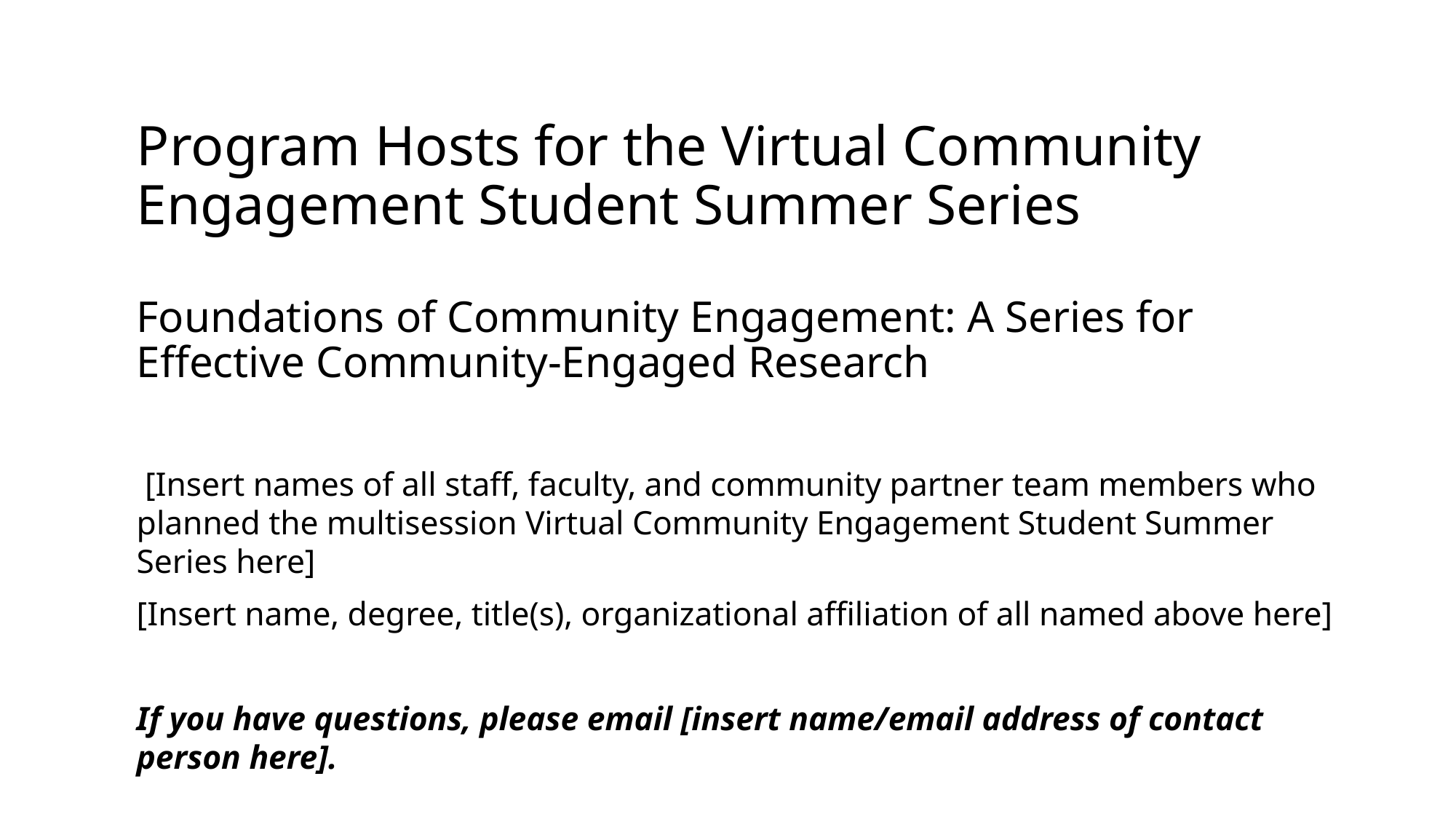

# Program Hosts for the Virtual Community Engagement Student Summer Series Foundations of Community Engagement: A Series for Effective Community-Engaged Research
 [Insert names of all staff, faculty, and community partner team members who planned the multisession Virtual Community Engagement Student Summer Series here]
[Insert name, degree, title(s), organizational affiliation of all named above here]
If you have questions, please email [insert name/email address of contact person here].

## Slide 3
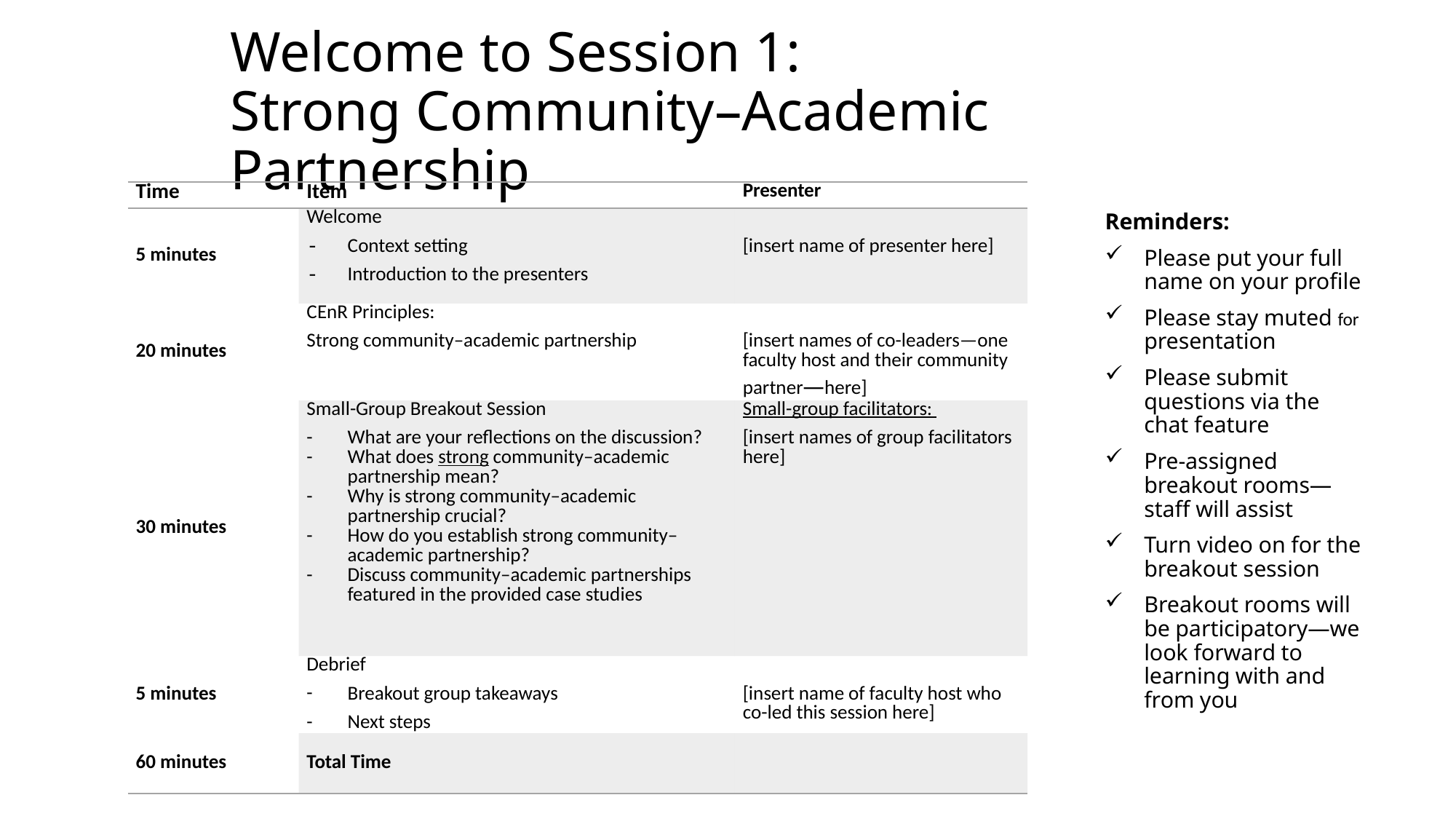

# Welcome to Session 1: Strong Community–Academic Partnership
| Time | Item | Presenter |
| --- | --- | --- |
| 5 minutes | Welcome Context setting Introduction to the presenters | [insert name of presenter here] |
| 20 minutes | CEnR Principles: Strong community–academic partnership | [insert names of co-leaders—one faculty host and their community partner—here] |
| 30 minutes | Small-Group Breakout Session What are your reflections on the discussion? What does strong community–academic partnership mean? Why is strong community–academic partnership crucial? How do you establish strong community–academic partnership? Discuss community–academic partnerships featured in the provided case studies | Small-group facilitators: [insert names of group facilitators here] |
| 5 minutes | Debrief Breakout group takeaways Next steps | [insert name of faculty host who co-led this session here] |
| 60 minutes | Total Time | |
Reminders:
Please put your full name on your profile
Please stay muted for presentation
Please submit questions via the chat feature
Pre-assigned breakout rooms—staff will assist
Turn video on for the breakout session
Breakout rooms will be participatory—we look forward to learning with and from you

## Slide 4
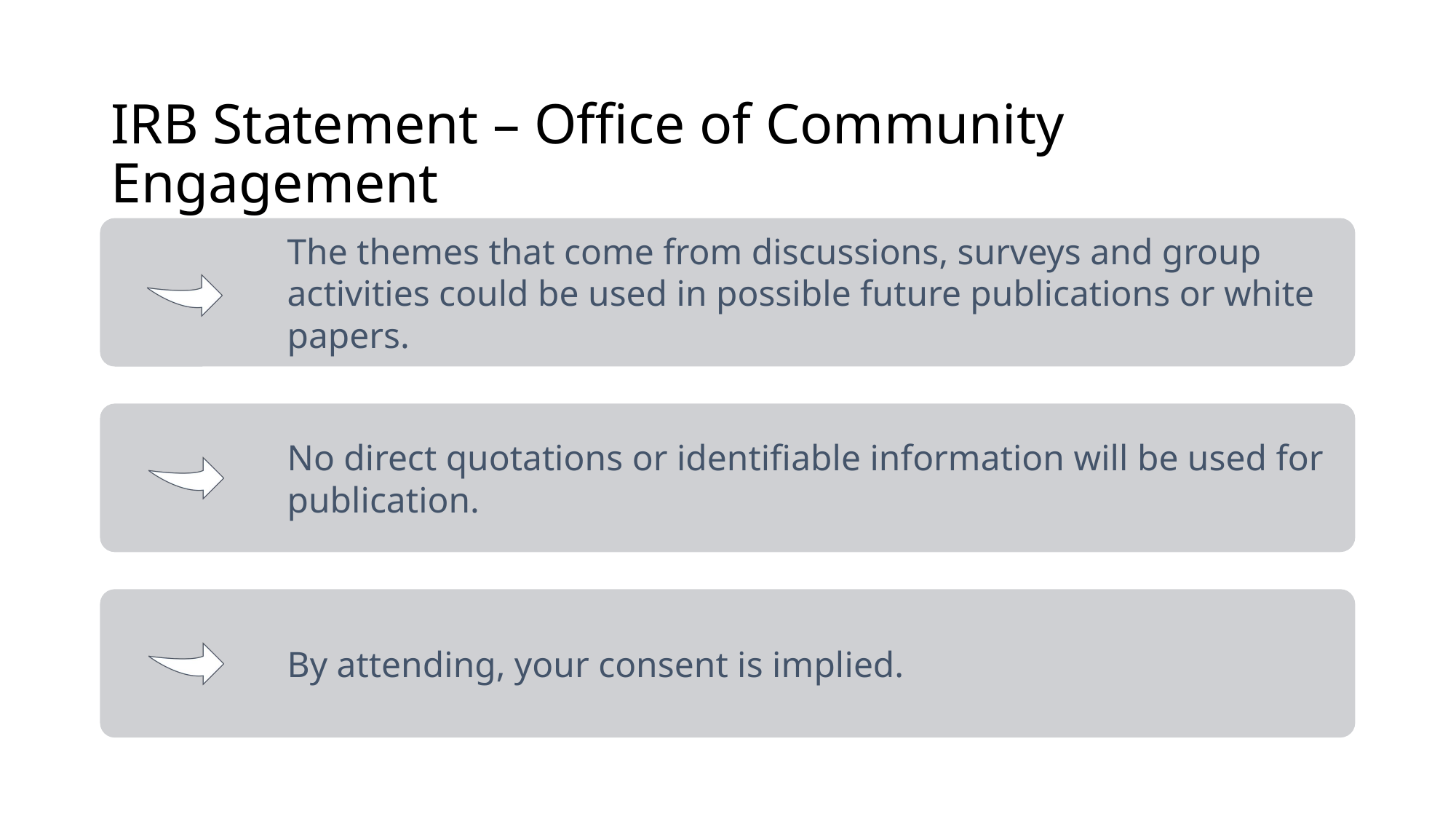

# IRB Statement – Office of Community Engagement

## Slide 5
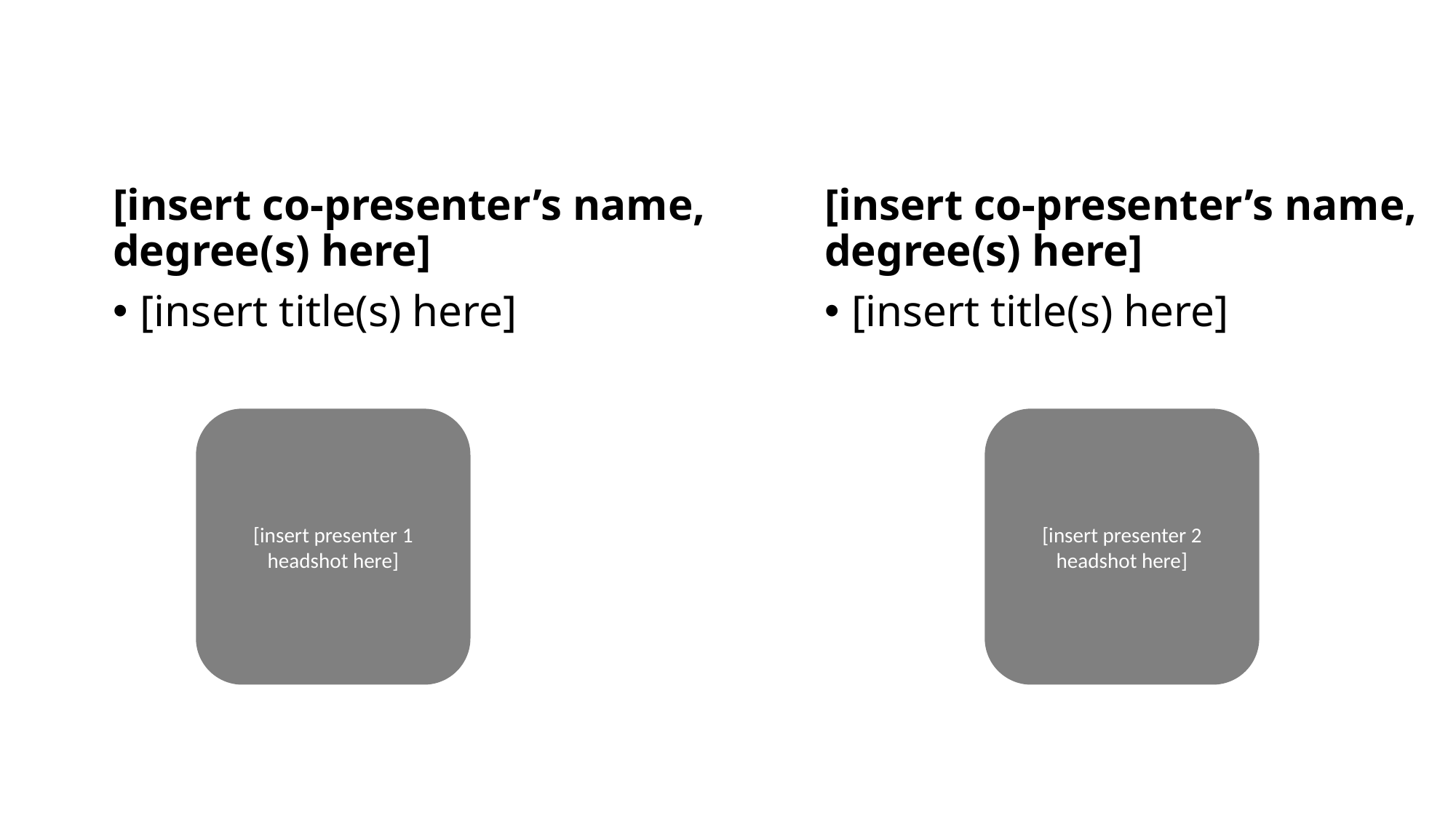

[insert co-presenter’s name, degree(s) here]
[insert title(s) here]
[insert co-presenter’s name, degree(s) here]
[insert title(s) here]
[insert presenter 1 headshot here]
[insert presenter 2 headshot here]

## Slide 6
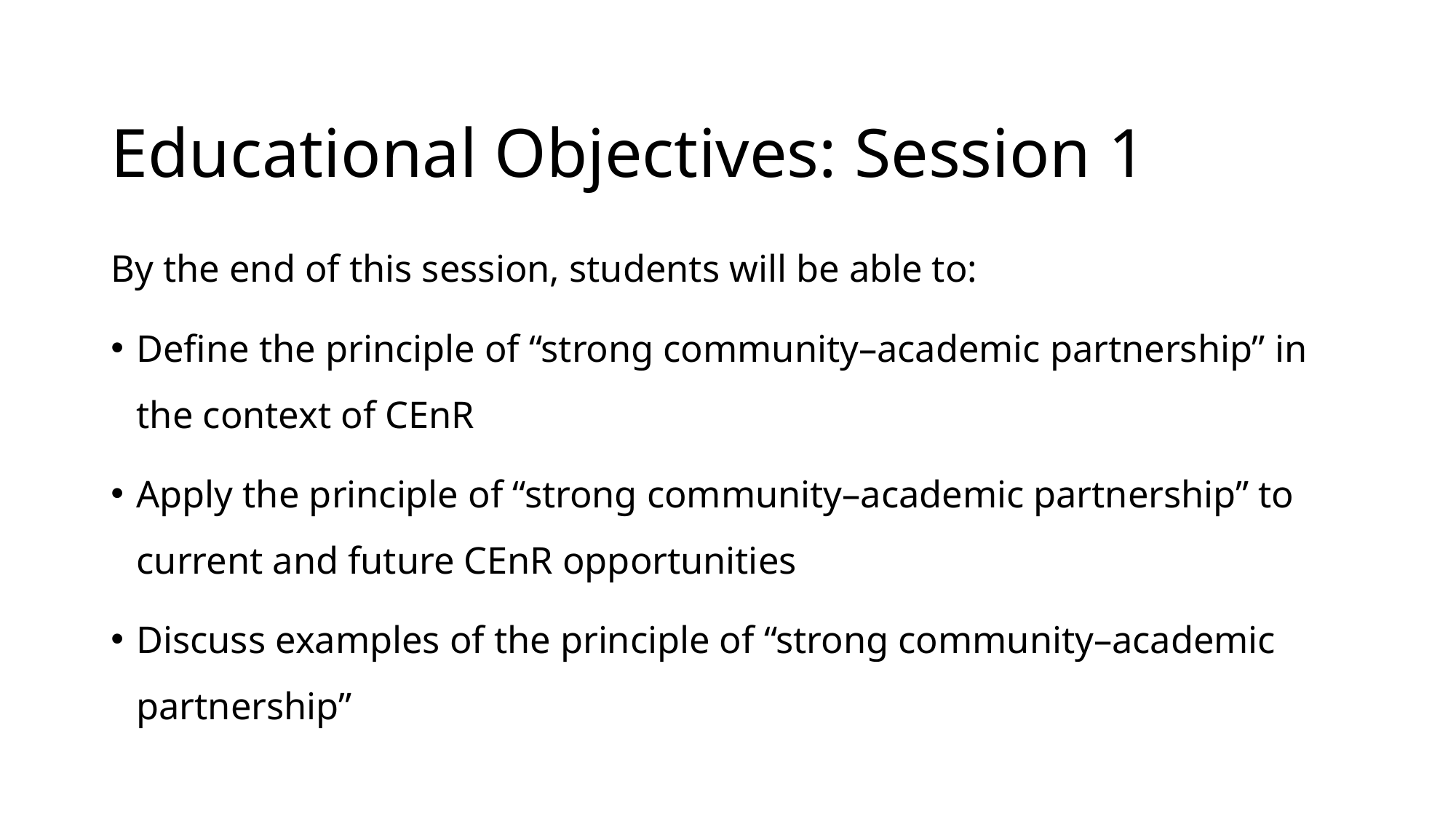

# Educational Objectives: Session 1
By the end of this session, students will be able to:
Define the principle of “strong community–academic partnership” in the context of CEnR
Apply the principle of “strong community–academic partnership” to current and future CEnR opportunities
Discuss examples of the principle of “strong community–academic partnership”

## Slide 7
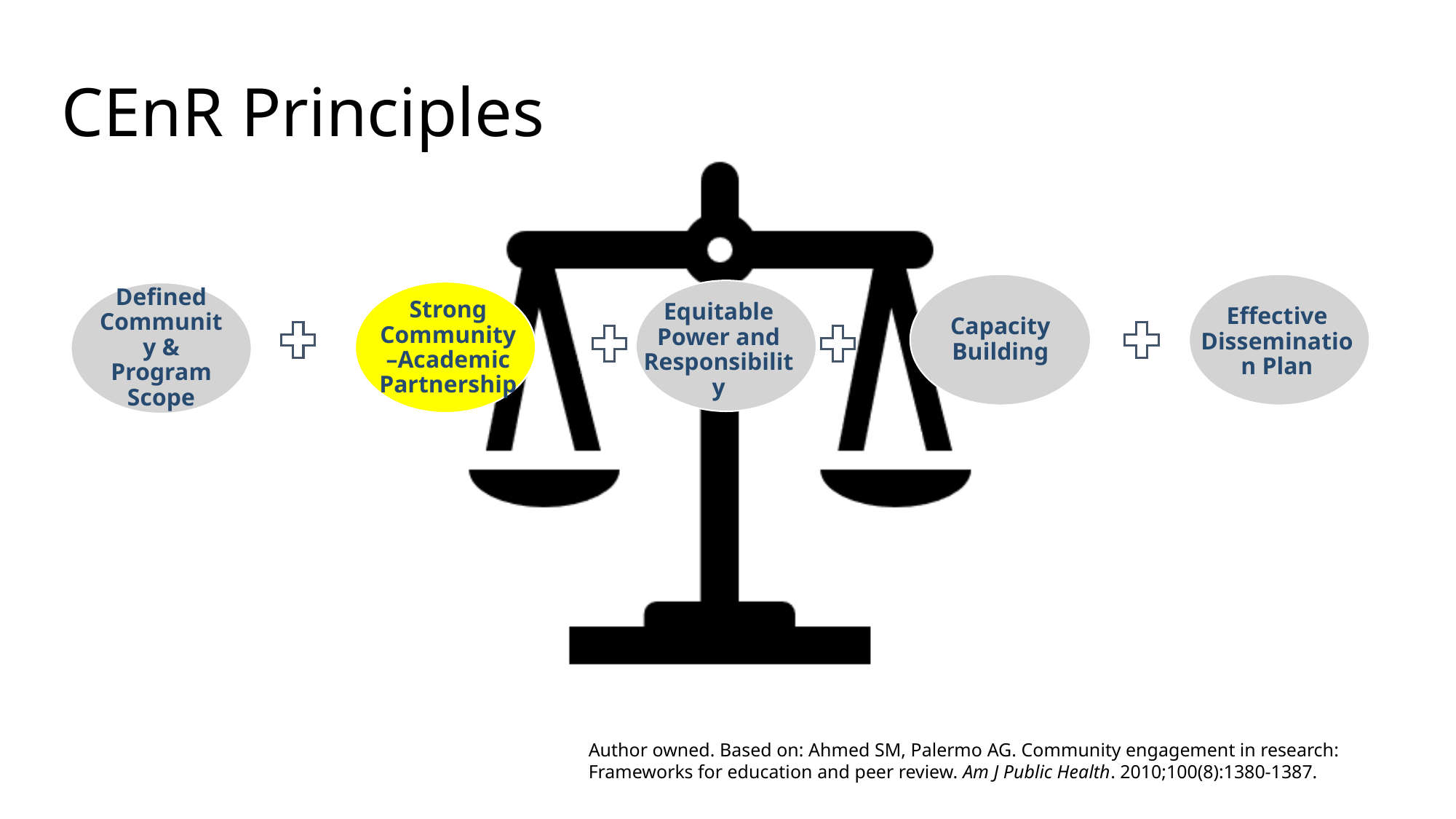

# CEnR Principles
Capacity Building
Effective Dissemination Plan
Equitable Power and Responsibility
Strong Community–Academic Partnership
Defined Community & Program Scope
Author owned. Based on: Ahmed SM, Palermo AG. Community engagement in research: Frameworks for education and peer review. Am J Public Health. 2010;100(8):1380-1387.

## Slide 8
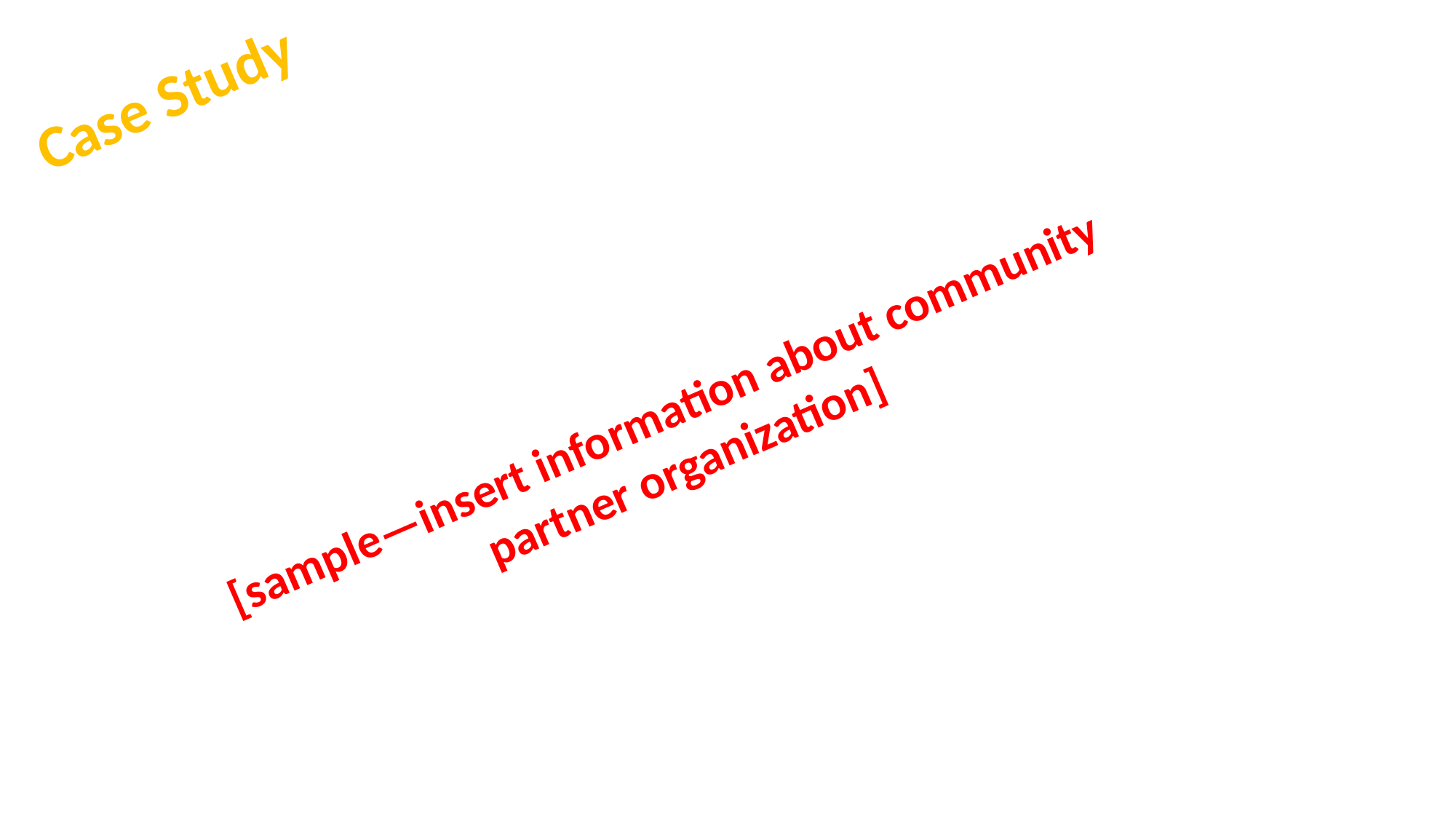

#
Case Study
[sample—insert information about community partner organization]

## Slide 9
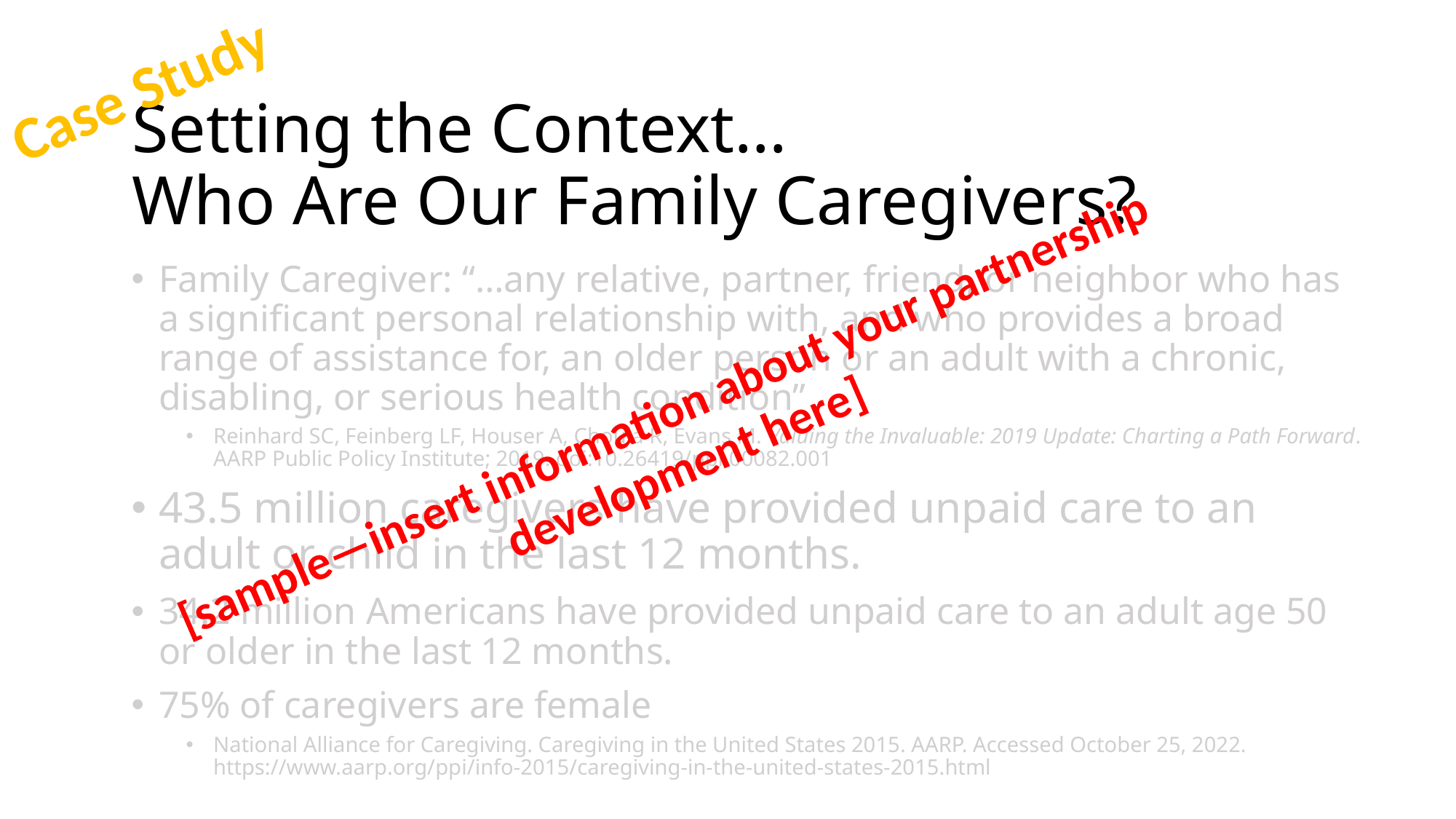

Case Study
# Setting the Context… Who Are Our Family Caregivers?
Family Caregiver: “…any relative, partner, friend, or neighbor who has a significant personal relationship with, and who provides a broad range of assistance for, an older person or an adult with a chronic, disabling, or serious health condition”
Reinhard SC, Feinberg LF, Houser A, Choula R, Evans M. Valuing the Invaluable: 2019 Update: Charting a Path Forward. AARP Public Policy Institute; 2019. doi:10.26419/ppi.00082.001
43.5 million caregivers have provided unpaid care to an adult or child in the last 12 months.
34.2 million Americans have provided unpaid care to an adult age 50 or older in the last 12 months.
75% of caregivers are female
National Alliance for Caregiving. Caregiving in the United States 2015. AARP. Accessed October 25, 2022. https://www.aarp.org/ppi/info-2015/caregiving-in-the-united-states-2015.html
[sample—insert information about your partnership development here]

## Slide 10
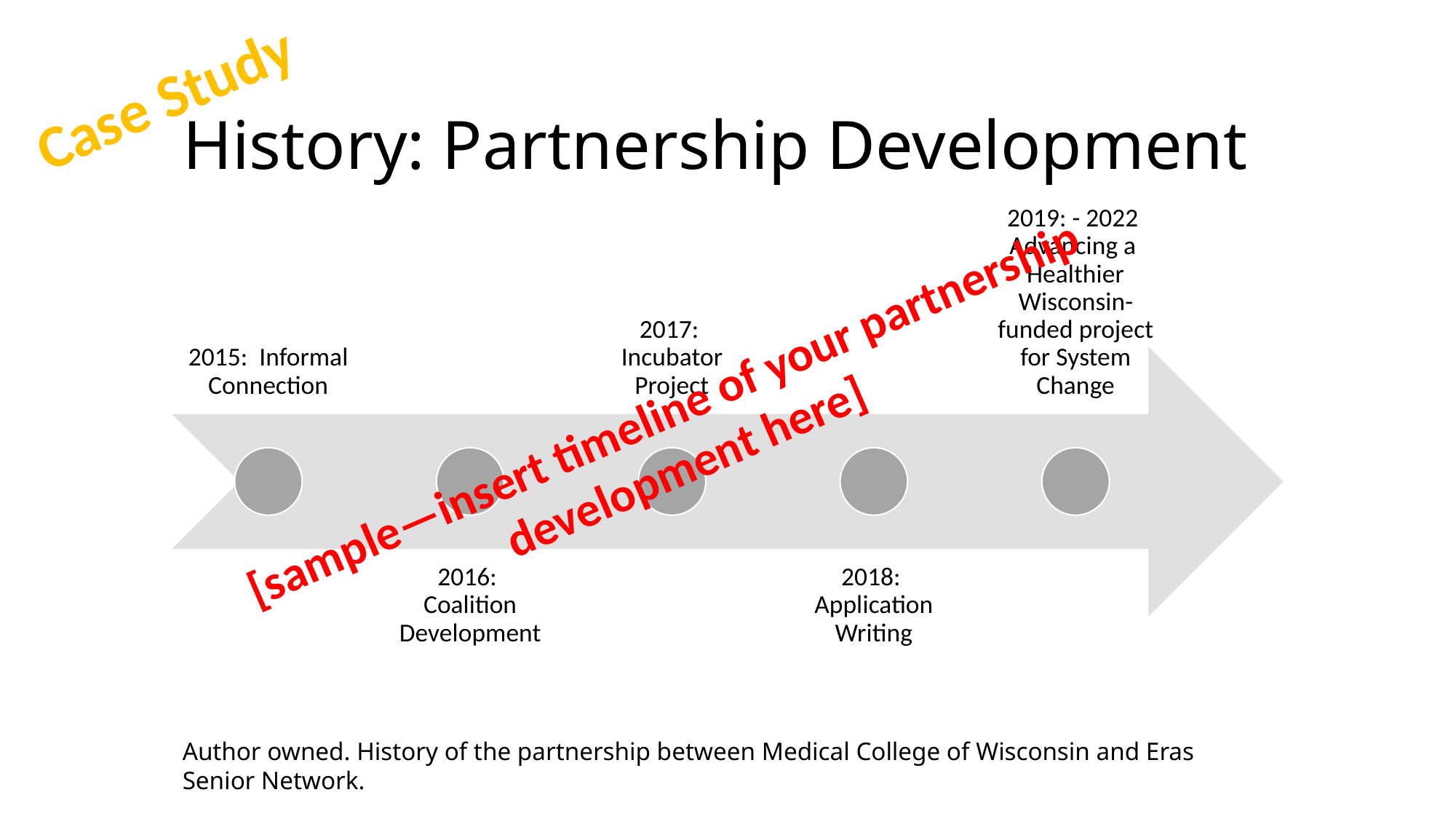

Case Study
# History: Partnership Development
[sample—insert timeline of your partnership development here]
Author owned. History of the partnership between Medical College of Wisconsin and Eras Senior Network.

## Slide 11
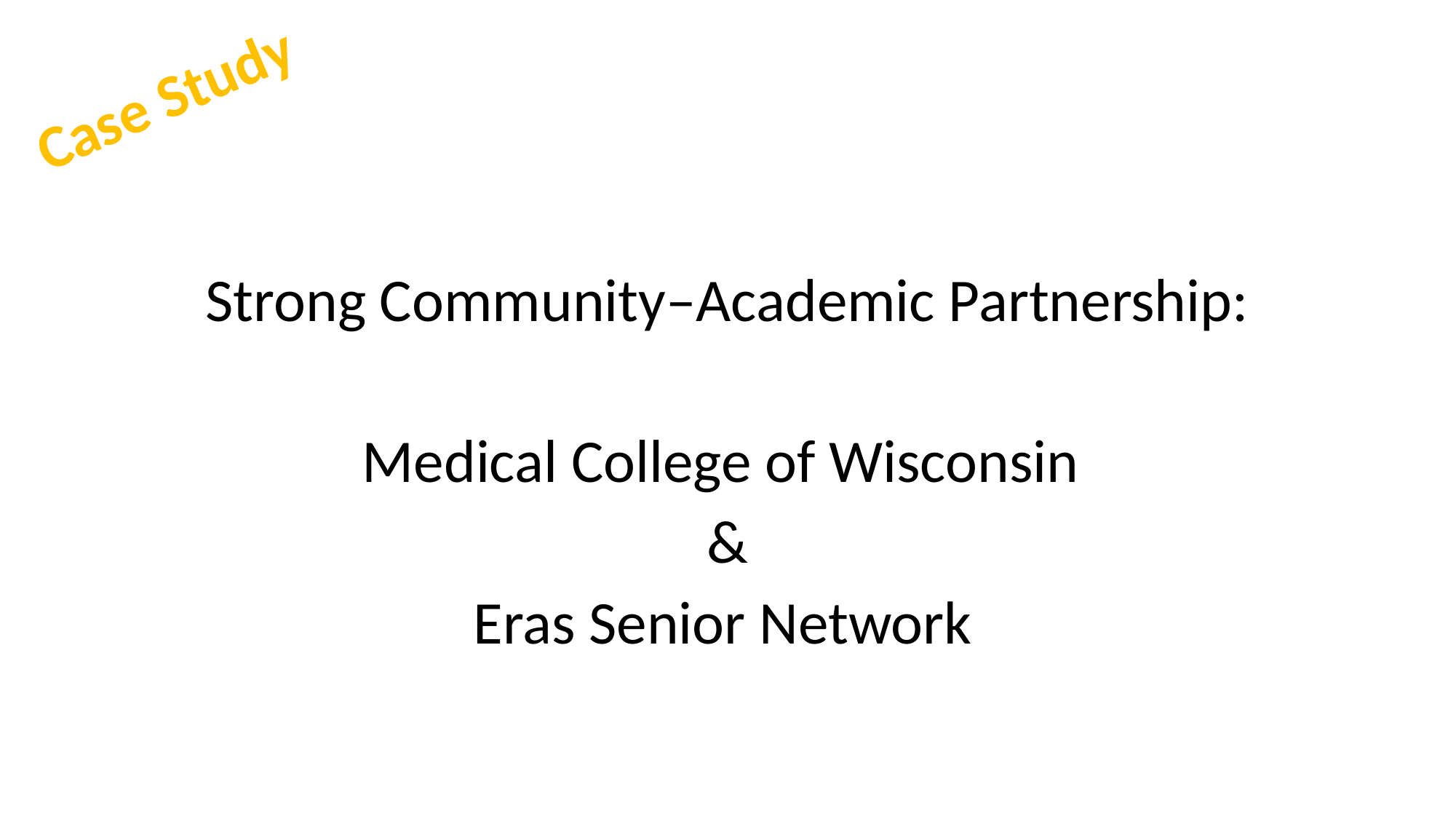

Case Study
Strong Community–Academic Partnership:
Medical College of Wisconsin
&
Eras Senior Network

## Slide 12
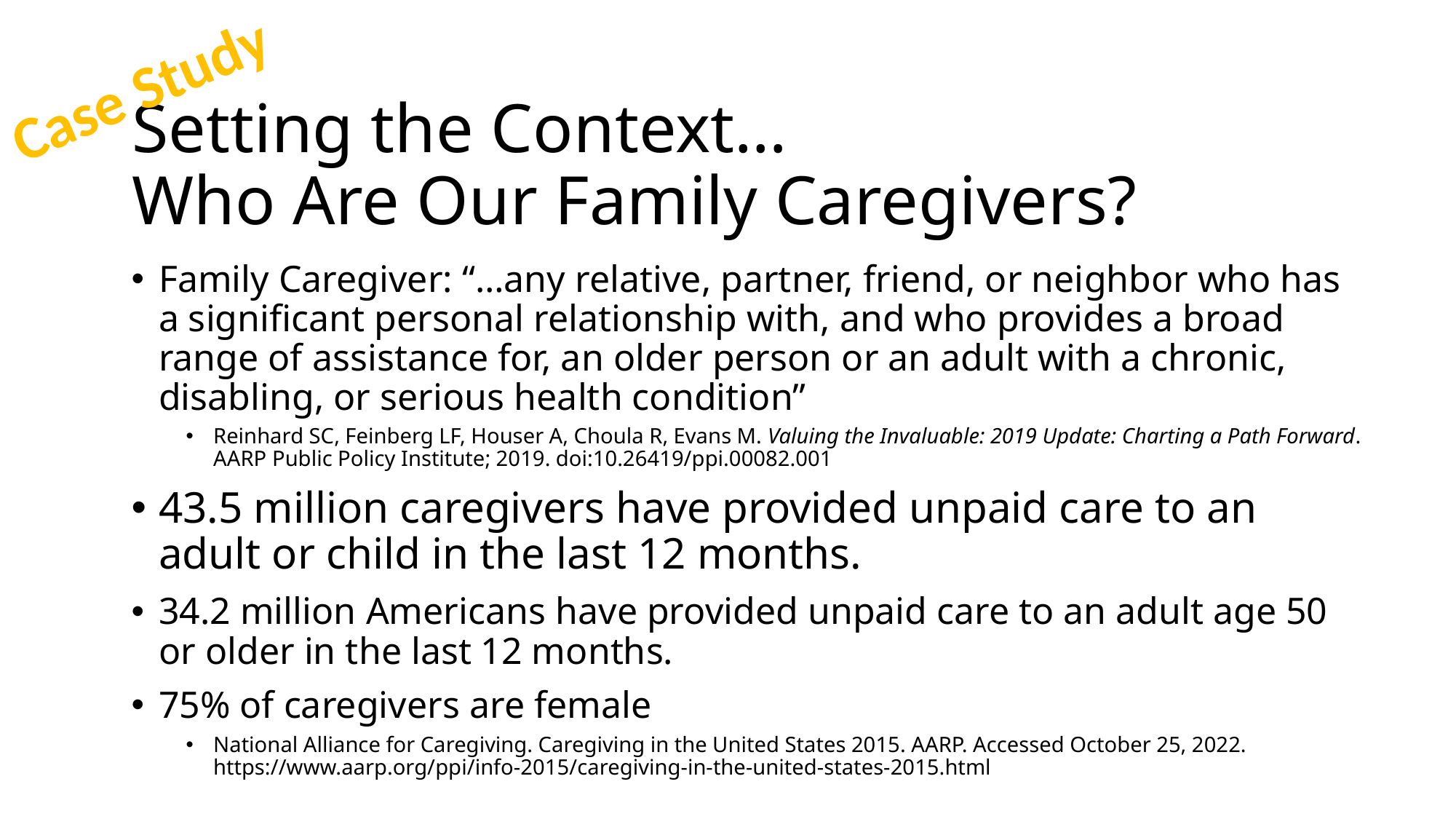

Case Study
# Setting the Context… Who Are Our Family Caregivers?
Family Caregiver: “…any relative, partner, friend, or neighbor who has a significant personal relationship with, and who provides a broad range of assistance for, an older person or an adult with a chronic, disabling, or serious health condition”
Reinhard SC, Feinberg LF, Houser A, Choula R, Evans M. Valuing the Invaluable: 2019 Update: Charting a Path Forward. AARP Public Policy Institute; 2019. doi:10.26419/ppi.00082.001
43.5 million caregivers have provided unpaid care to an adult or child in the last 12 months.
34.2 million Americans have provided unpaid care to an adult age 50 or older in the last 12 months.
75% of caregivers are female
National Alliance for Caregiving. Caregiving in the United States 2015. AARP. Accessed October 25, 2022. https://www.aarp.org/ppi/info-2015/caregiving-in-the-united-states-2015.html

## Slide 13
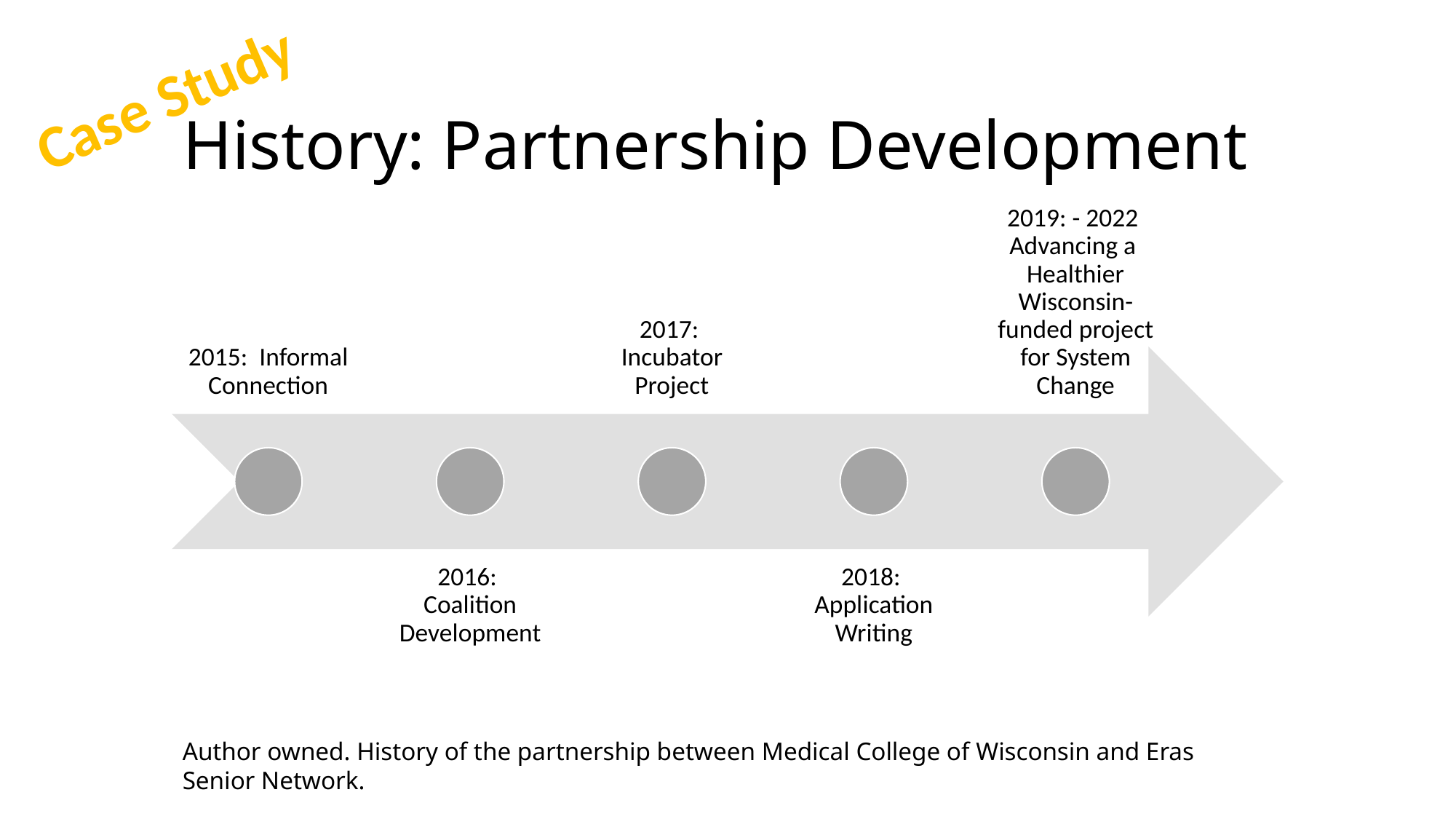

Case Study
# History: Partnership Development
Author owned. History of the partnership between Medical College of Wisconsin and Eras Senior Network.

## Slide 14
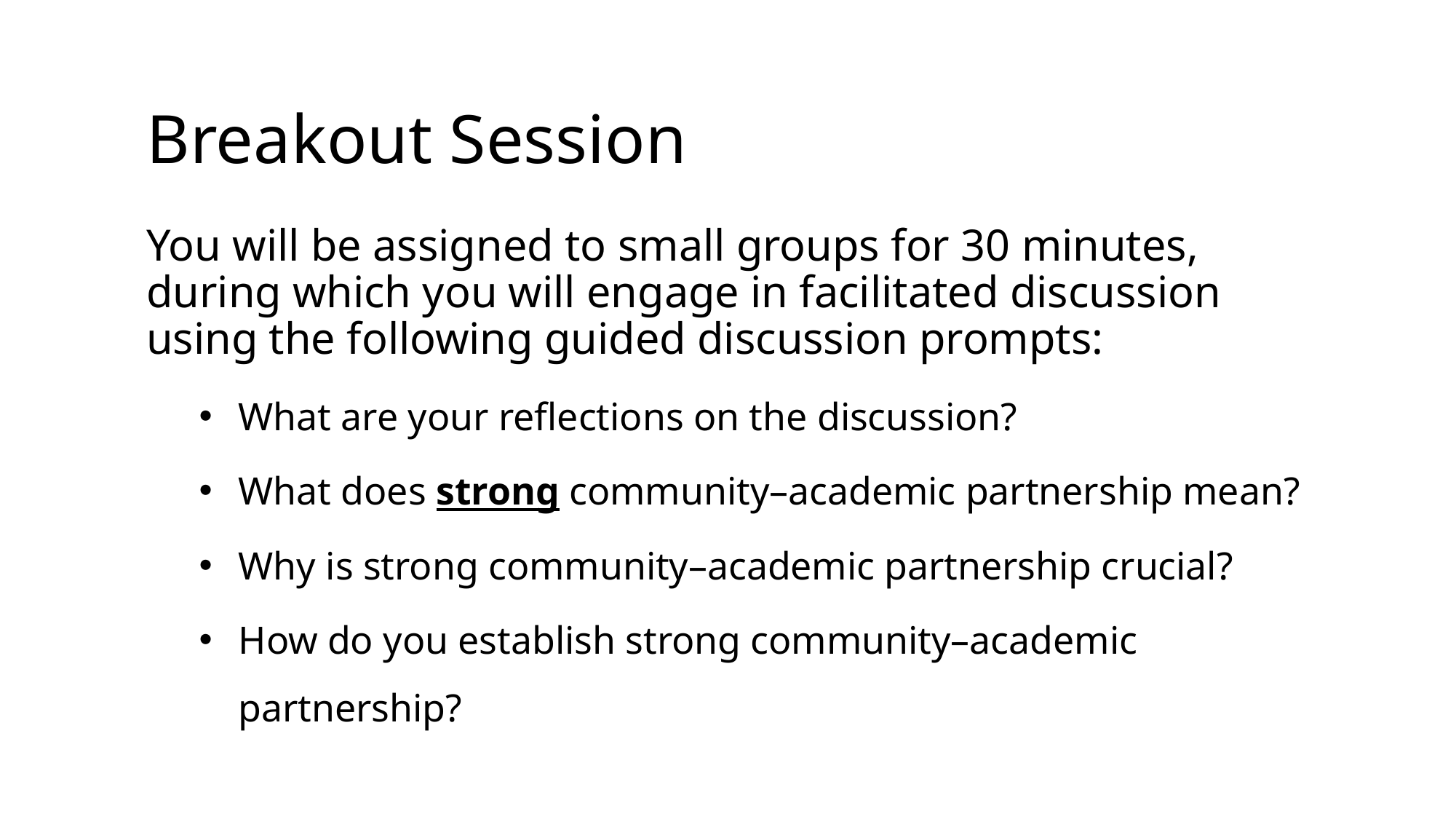

# Breakout Session
You will be assigned to small groups for 30 minutes, during which you will engage in facilitated discussion using the following guided discussion prompts:
What are your reflections on the discussion?
What does strong community–academic partnership mean?
Why is strong community–academic partnership crucial?
How do you establish strong community–academic partnership?

## Slide 15
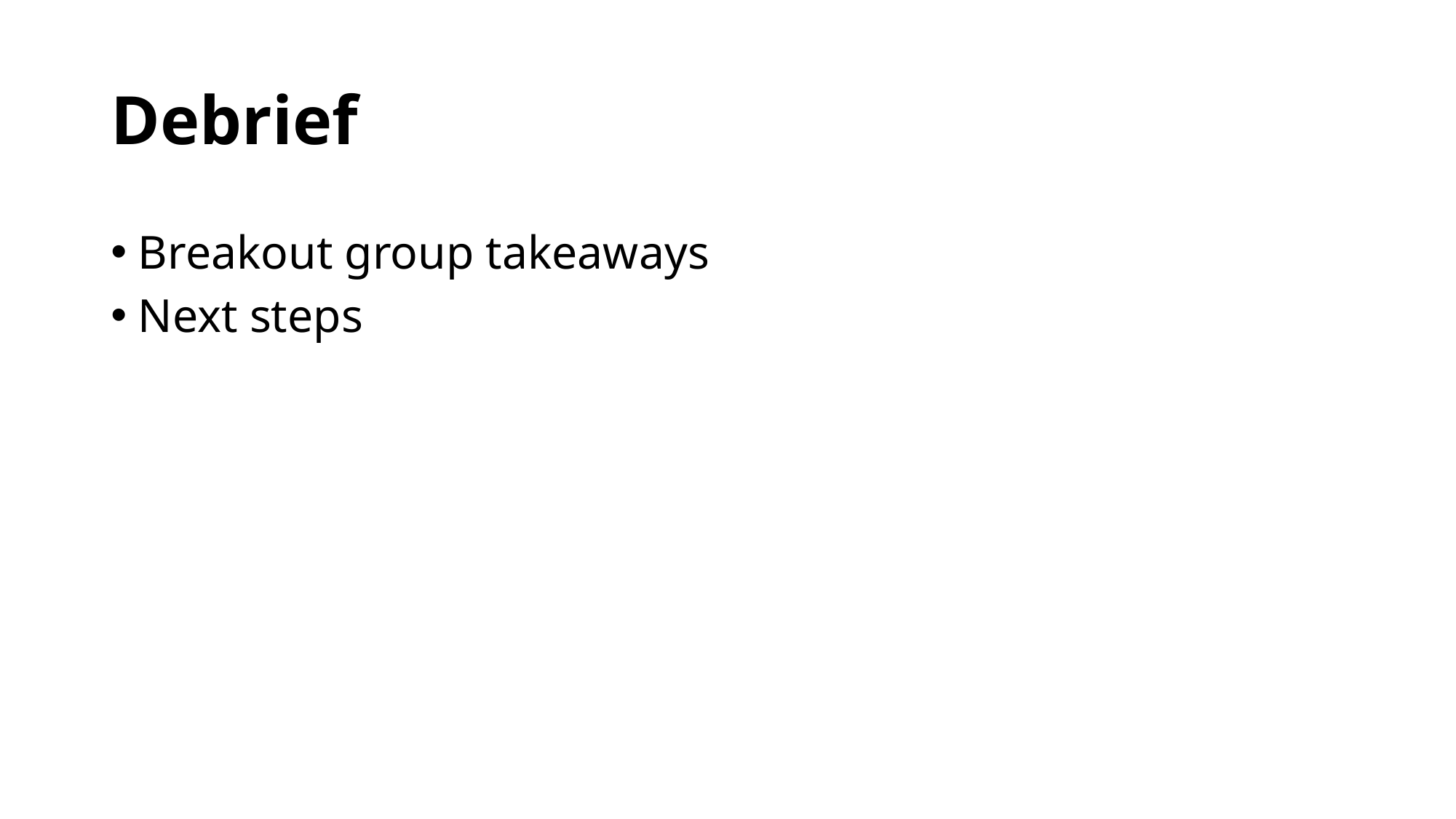

# Debrief
Breakout group takeaways
Next steps

## Slide 16
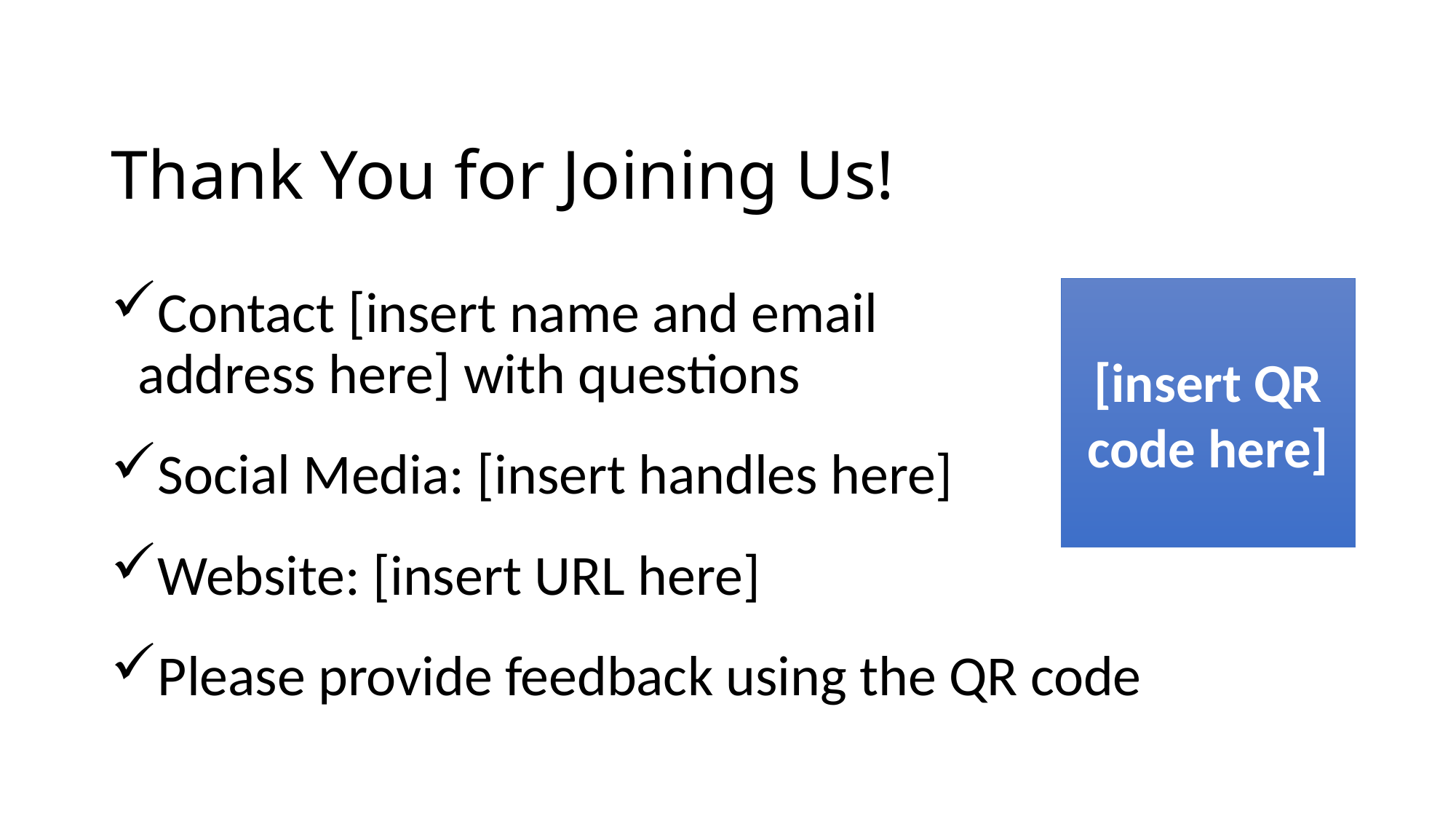

# Thank You for Joining Us!
Contact [insert name and email
address here] with questions
Social Media: [insert handles here]
Website: [insert URL here]
Please provide feedback using the QR code
[insert QR code here]

## Slide 17
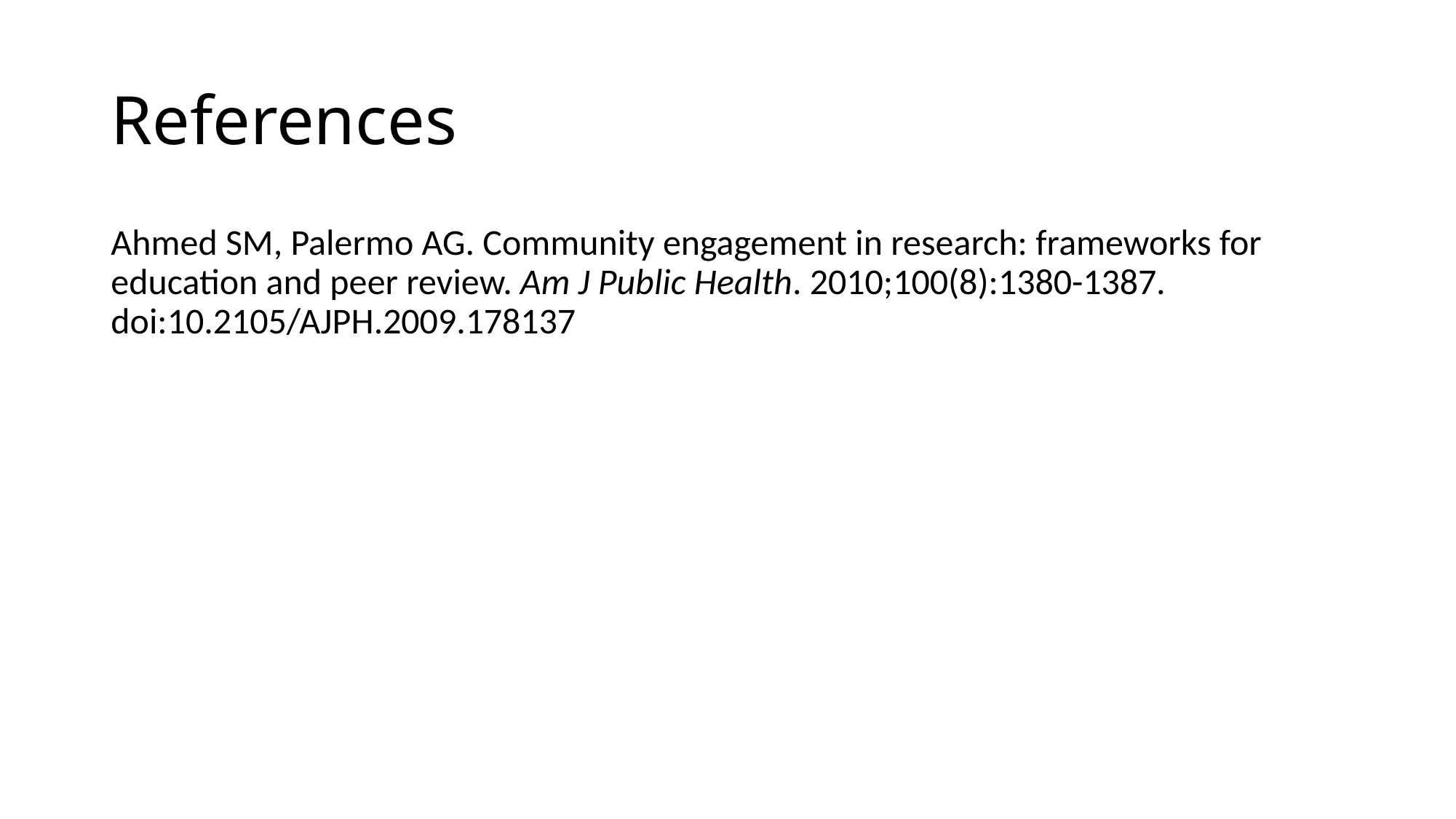

# References
Ahmed SM, Palermo AG. Community engagement in research: frameworks for education and peer review. Am J Public Health. 2010;100(8):1380-1387. doi:10.2105/AJPH.2009.178137
