# Supplementary material for: Foundations of Community Engagement: A Series for Effective Community-Engaged Research
Source: MedEdPORTAL. 2023 Oct 10;19:11350. doi: 10.15766/mep_2374-8265.11350 (PMC10562524; doi:10.15766/mep_2374-8265.11350)
Supplement: Supplementary file 1 — CE Didactic Session Slides.pptxApplication for Small-Group Series.docxCommunity-Academic Partnership Slides.pptxEquitable Power and Responsibility Slides.pptxEquitable Power and Responsibility Case Studies.docxCapacity Building and Dissemination Slides.pptxFacilitator Guide.docxCE Didactic Session Evaluation.docxSmall-Group Session Evaluation.docx [file mep_2374-8265.11350-s001.zip › G. Facilitator Guide.docx]

**Foundations of Community Engagement: A Series for Effective Community-Engaged Research**

**Comprehensive Facilitator Guide**

**Overview and Purpose**

This 4-session series addresses an unmet need to expose medical students to community engagement (CE) and community-engaged research (CEnR) principles, an under-emphasized area in medical education. The series involves an initial didactic session focused on foundational CEnR principles and 3 hybrid lecture–discussion sessions demonstrating active community–academic or faculty–learner partnerships demonstrating application of the CEnR principles. This facilitator guide includes an overview of the series and individual guides for each session.

**Learning Objectives**

By the end of this series, learners will be able to:

1. Define the principles of CEnR
2. Apply the principles of CEnR to current and future CEnR opportunities
3. Discuss examples of the principles of CEnR

**Curriculum Description**

The series commences with a mandatory initial didactic session for all students enrolled in a summer research program, followed by the 3 subsequent lecture–discussion sessions conducted as optional sessions for a smaller cohort of students accepted to the program. (The application is available as Appendix B.)

The first session introduces CEnR conceptually to all summer research students. Given the breadth of content and introductory nature of this session, a didactic format is used with interactive questions and opportunities for comments and questions. Students are then invited to apply for the second part of the summer program—a 3-session course in which core CEnR principles, introduced in the first session, are explored in more depth. Each of these sessions represents a deep dive into 1 or 2 core CEnR principles and highlights a practical case study of a CEnR community–academic partnership or faculty–learner interaction in the context of a CEnR program. Academic and community partners in the program share their experiences and insights pertaining to the respective CEnR principles. Readings and small-group discussions are highlighted. Mentorship components are offered as well, including invitations to consult with CEnR faculty for career advisement.

Some core faculty presenters participate in the first didactic session and a subsequent lecture–discussion session—inviting either a community partner or learner to join with them in the lecture–discussion session. There is flexibility in terms of the resources and availability of the presenters for how to fill the following roles. It may even be possible for a single experienced faculty presenter to lead all sessions, provided they include community and learner voices of sufficient variety and depth to augment their perspective.

**Session Agenda**

| **Title** | **Description** |
| --- | --- |
| *Community-Engaged Research Principles: A Didactic Session for Medical Students* | - Review current definitions and principles of CE and CEnR - Discuss evolution of and rationale for CE - Learn about the state of CEnR at the host academic institution and how it intersects with other core pillars - Discuss examples of CEnR occurring at the host academic institution |
| Session 1: *Strong Community–Academic Partnership* | - Define the principle of strong community–academic partnership in the context of CEnR - Apply the principle of strong community–academic partnership to current and future CEnR opportunities - Discuss examples of the principle of strong community–academic partnership |
| Session 2: *Equitable Power and Responsibility* | - Define the principle of equitable power and responsibility in the context of CEnR - Apply the principle of equitable power and responsibility to current and future CEnR opportunities - Discuss examples of the principle of equitable power and responsibility |
| Session 3: *Capacity Building and Effective Dissemination* | - Define the principles of capacity building and effective dissemination in the context of CEnR - Apply the principles of capacity building and effective dissemination to current and future CEnR opportunities - Discuss examples of the principles of capacity building and effective dissemination |

Agendas for 60-minute sessions are provided for all sessions. Our research indicates that many participants in 60-minute sessions would have preferred longer small-group and debrief sessions, so 90‑minute agendas are also provided for the 3 small-group sessions.

**Materials**

A slide deck with facilitator notes has been provided for all 4 sessions in the series:

- Appendix A – Principles of CE didactic session slides
- Appendix C – Strong community-academic partnership slides
- Appendix D – Equitable power and responsibility slides
- Appendix F – Capacity building and effective dissemination slides

A pair of case studies are provided for the session on equitable power and responsibility:

- Appendix E – Equitable power and responsibility case studies

These materials have been formatted for easy implementation. Slides and facilitator notes have all been provided in editable formats and clearly indicate where institution- and community partner-specific information should be added. Clear guidance is provided throughout.

Facilitator notes in the slide decks complement this guide, providing clear instructions for session facilitation with timing, instructions, and, for standard content slides, scripts that may be read by the facilitator verbatim (indicated by italicized text), references for source materials, etc.

**Roles**

This curriculum’s structure allows for flexibility in roles, with guidance for facilitation and presentation included in this guide and the facilitator notes in the slide decks. In this curriculum and facilitator guide, role-based guidance uses these loose definitions of the roles:

- Student or participant – attendee or learner
- Facilitator – coordinates the presentation materials and facilitates or moderates the presentation of the session. May be a staff or faculty member. Role may be easily filled by the lead faculty presenter
- Presenter – faculty member, community-partner, or medical student with CEnR experience
- Small-group facilitator – a faculty member or near-peer

**Didactic Session: *Community-Engaged Research Principles***

**Session Agenda (60 minutes)**

| **Time** | **Item (Presenter)** |
| --- | --- |
| 5 minutes | Welcome (all presenters) |
| 25 minutes | Community-Engaged Research (CEnR) Principles (institutional community engagement leader/researcher) |
| 5 minutes | Q & A (institutional community engagement leader/researcher) |
| 8 minutes | CEnR: Expanding our Purview (community-engaged researcher and/or clinician) |
| 8 minutes | CEnR: A Connecting Point for Students and Community (community-engaged researcher and/or clinician) |
| 4 minutes | Introduction to Foundations of Community Engagement: A Series for Effective Community-Engaged Research  How to Apply (community-engaged researcher and/or clinician) |
| 5 minutes | Q & A (community-engaged researcher and/or clinician) |

**Materials**

Principles of CE didactic session slides (Appendix A) with facilitator notes

**Session Preparation**

Student

None required

Facilitator

Before attending the session, you should:

1. Determine session duration and adjust agendas as needed.
2. Review slides, inserting program-specific information and presenter-provided materials as needed.
3. Prepare interactive polling using polling software. (We used Poll Everywhere.)
4. Determine which slides will be led by which presenter (unless a single presenter is leading the entire session). Recommended breakdown would be: most senior presenter leads introduction and CE principles sections; more junior presenter(s) lead the remaining sections.
5. Determine any modifications to content or procedures for subsequent 3 sessions and integrate them into this presentation.

**Role and Responsibilities**

Faculty Presenter

- Your role is to provide engaging and informative perspectives into the high-level context of CE in a manner accessible to medical students who may or may not have any background knowledge of CE.
- Although this session is designed as a background didactic presentation with less interaction than subsequent sessions, presenters are advised to encourage participant questions via chat (virtual format) or raised-hand (in-person format) participation during Q & A components.
- If multiple presenters or a presenter and staff person, e.g., education coordinator, are available, those not presenting may keep time and identify/triage questions in chat (virtual format) or raised-hand (in-person format) participation.
- Program application information (if used) and session evaluation QR code (if used) should be displayed on a final slide and also emailed to participants after the session along with slides and any other session procedural information per below.

**Post-session Work – Faculty and Staff**

- Email QR code or link to session evaluation immediately after session.
- If requiring students to apply to subsequent 3-session program:
  - Send email with application information to all session participants.
  - Remind students of application via email format approximately 1 week before deadline.
  - After deadline, compile and review applications.
  - Reconcile number of acceptable applications with constraints of small-group format. A low threshold for accepting applications, using the application not to screen out applicants but to learn about applicants’ interests and background, inspires a sense of commitment to the program. There are a number of ways to facilitate the subsequent 3 sessions for different numbers of engaged students—the limiting factor is attracting and coordinating small-group facilitators who can realistically provide high-quality facilitation for up to 8-10 students per small group. Recruiting more facilitators or asking students to engage with near-peer facilitators could be options for expanding the pool of accepted applicants. In our experience, we have been able to adjust the structure of the subsequent sessions to accommodate all suitable applicants.
- Depending on the accepted pool of applicants, schedule the 3 follow-up sessions. We have found that a 4-week gap between the initial session and subsequent sessions, held 1 week apart, was a suitable rhythm. The application process mentioned above was conducted with a 2- or 3-week application deadline after the initial session, and then a rapid acceptance and scheduling of subsequent sessions. If an application process will not be used between the didactic session and small-group sessions, it may be advisable to shorten the timeline between the didactic and first small-group session. Be mindful of local academic calendar considerations, including common exam periods, vacations, and interview seasons to enable participation of interested students.
- Send acceptant letters with links to readings for small-group session 1 (*Strong Community–Academic Partnerships*).
- Send an email reminder 2-3 days prior to the next session.

**References**

Ahmed SM, Palermo AG. Community engagement in research: frameworks for education and peer review. *Am J Public Health*. 2010;100(8):1380-1387. doi:10.2105/AJPH.2009.178137

American Council on Education. The 2024 Elective Classification for Community Engagement. https://carnegieelectiveclassifications.org/the-2024-elective-classification-for-community-engagement/

Centers for Disease Control and Prevention. *Principles of Community Engagement* (1st ed.). Atlanta, GA: CDC/ATSDR Committee on Community Engagement; 1997.

Dharamsi S, Espinoza N, Cramer C, Amin M, Bainbridge L, Poole G. Nurturing social responsibility through community service-learning: lessons learned from a pilot project. *Med Teach*. 2010;32(11):905-911. doi:10.3109/01421590903434169

Hood CM, Gennuso KP, et al. County health rankings: relationships between determinant factors and health outcomes. *Am J Prev Med.*2016;50(2):129-135.

Liaison Committee on Medical Education. Standards, Publications, & Notification Forms. Accessed January 11, 2022. https://lcme.org/publications/

Meurer LN, Young SA, Meurer JR, et al. The urban and community health pathway: preparing socially responsive physicians through community-engaged learning. *Am J Prev Med*. 2011;41(4 Suppl 3):S228-236. doi:10.1016/j.amepre.2011.06.005**Session 1: *Strong Community–Academic Partnership***

**Session Agenda (60-90 minutes)**

| **Time** | **Item (Presenter)** |
| --- | --- |
| 5 minutes | Welcome (community-engaged researcher and/or clinician)   - Context setting - Presenter introductions |
| 20-25 minutes | CEnR Principles: Strong Community–Academic Partnership (community-engaged researcher and/or clinician, community partner) |
| 30-50 minutes | Small-Group Breakout Session (facilitated by presenters; other community-engaged researchers/clinicians/staff)   - Introduction - Facilitated discussion questions - What are your reflections on the discussion? - What does strong community–academic partnership mean? - Why is strong community–academic partnership crucial? - How do you establish strong community–academic partnership? - Discuss community–academic partnerships featured in the provided case studies |
| 5-10 minutes | Debrief (community-engaged researcher/clinician)   - Breakout group takeaways - Next steps |

**Materials**

Strong community-academic partnership slides (Appendix C) with facilitator notes

**Session Preparation**

Student

- Read the following:
  - Ahmed SM, Palermo AG. Community engagement in research: Frameworks for education and peer review. *Am J Public Health.* 2010;100(8):1380-1387.
  - Ahmed SM, Neu Young S, DeFino MC, Franco Z, Nelson DA. Towards a practical model for community engagement: advancing the art and science in academic health centers. *J Clin Transl Sci.* 2017;1(5):310-315.

Facilitator

Before attending the session, you should:

1. Determine session duration and adjust agendas as needed.
2. Review slides, inserting program-specific information and presenter-provided materials as needed.

Faculty Presenter

1. Consider a community partner or student who would be suitable to join and assist you in illustrating the dynamics of this CE principle. Given the topic, it may be best to recruit a community partner to assist with this—ideally one with whom you have a strong relationship with a robust history that can provide multiple cases in point that will exemplify the partnership for students. It is advisable to identify this community partner and request their assistance far in advance and, if possible, to compensate your community partner for their time in a meaningful way.
2. Meet with your identified community partner in advance of this session to determine the most effective way to share your partnership and illustrate the principle of strong community–academic partnership. There are many possible ways to illustrate this. An example is given in Appendix C. Suitable options that have been used in the past include an open, reflective discussion; an advice-giving session; a format in which one partner interviews the other and draws out the historical and present context of the partnership.
3. Prepare the case study slides and provide them to the facilitator in advance of the session.
4. Determine if a facilitator or another presenter will be included to introduce presenters, set context, or debrief the session. If additional presenters or small-group facilitators are required, they should be recruited far in advance.

**Roles and Responsibilities During Session**

Presenter

- Your role is to provide an engaging and informative example of the CEnR principle of strong community–academic partnership from the real-world experience you and your community partner have had in this area. The above preparatory work will help you guide the conversation, but learners will benefit from any reflections or lessons learned you would share in this area. Learners may or may not have experience in CEnR but, if this has been offered as an optional session, have chosen to engage in this voluntary program indicating an interest in the topic.
- During the large-group component of the session, the focus will be on interaction between community partner and academic partner. Interaction will be encouraged to take place during the small-group breakout sessions following the initial large-group discussion. Presenters may choose to take questions via raised hand or chat box, or they may choose to direct questions to the small-group discussions.
- Following the small-group portion of the session, there will be a return to large-group debrief. A takeaway may be requested from each group if time permits. A reminder for the next scheduled session may be provided before the session concludes. If used, a post-session evaluation QR code should be displayed on the final slide and emailed to students following the session.

Small-Group Facilitator

- Small-group facilitators will be responsible for guiding a group of learners through reflection on the community and academic partner discussion, as well as prepared reflective questions. Small-group facilitators may be the community and academic partners who had presented in the session, other community-engaged academic and community leaders, or community-engaged staff or learners. Ideally, small-group facilitators will lead groups of 4-6 learners, although this number may increase or decrease depending on attendance and small-group facilitator recruitment.
- Small-group facilitators are encouraged to first create an atmosphere of openness and comfort. It is recommended to start by asking everyone to lean into the discussion by either putting away their devices (in-person) or turning on their cameras (virtual). A short introduction may be useful, with the small-group facilitator starting by introducing themself and the reason they have been inspired to join today, then requesting the participants do the same.
- Generate and guide discussion of the learners. Instead of speaking extensively, ask probing questions, invite reflection, and stimulate connection between content, experience, and intention. Learners of CEnR have widely variable levels of experience in the area and can often teach and advise one another, especially on institutionally accessible resources and best practices from a student perspective. Seek to elevate the voices of participants and reinforce and add perspective to them, not to correct or discredit the messages shared.
- If possible, expand the small-group discussion to 50 minutes to enable more thorough and personalized levels of discussion. If this is not possible, it may be necessary to narrow the focus of the questions or areas of discussion posed to the group.
- The prepared questions should not constrain small-group facilitators. Small-group facilitators are invited to provide additional prompts or areas of reflection.

**Post-session Work – Faculty and Staff**

1. Email QR code or link to session evaluation immediately after session.
2. Send an email reminder 2-3 days prior to the next session.
3. Distribute the case studies to participants so they may prepare for the next session.

**References**

Ahmed SM, Palermo AG. Community engagement in research: Frameworks for education and peer review. *Am J Public Health.* 2010;100(8):1380-1387.**Session 2: *Equitable Power and Responsibility***

**Session Agenda (60-90 minutes)**

| **Time** | **Item (Presenter)** |
| --- | --- |
| 5 minutes | Welcome (facilitator, community-engaged researcher and/or clinician)   - Context setting - Presenter introductions |
| 20-25 minutes | CEnR Principles: Equitable Power and Responsibility (community-engaged researcher and/or clinician, community partner) |
| 30-50 minutes | Small-Group Breakout Session (facilitated by presenters; other community-engaged researchers/clinicians/staff)   - Introduction - Facilitated discussion questions   - How can we balance power and responsibility in medicine and community-engaged research?   - How might you incorporate this into your own work and/or learning?   - Discuss community–academic partnerships featured in the provided case studies |
| 5-10 minutes | Debrief (community-engaged researcher/clinician)   - Breakout group takeaways - Next steps |

**Materials**

Equitable power and responsibility slides (Appendix D) with facilitator notes

Case studies (Appendix E) – to be distributed to students prior to or at start of session if virtual or provided at start of session if in person.

**Session Preparation**

Student

Review the assigned case studies and related discussion questions (Appendix E).

Program Facilitator

Before attending the session, you should:

1. Determine session duration and adjust agendas as needed.
2. Review slides and case studies (Appendix E), inserting program-specific information and presenter-provided materials as needed.

Faculty Presenter

Before attending the session, you should:

1. Consider a community partner or student who would be suitable to join and assist you in illustrating the dynamics of this CEnR principle. Given the topic, it may be best to recruit a community partner to assist with this—ideally one with whom you have a strong relationship with a robust history that can provide multiple cases in point that will exemplify your mutual responsibility and power in the partnership. It is advisable to identify this community partner and request their assistance far in advance and, if possible, to compensate your community partner for their time in a meaningful way.
2. It is recommended to meet with your identified community partner in advance of this session to determine the most effective way to share your partnership and illustrate the principle of equitable power and responsibility. There are many possible ways to illustrate this. An example is given in Appendix D. Suitable options that have been used in the past include an open, reflective discussion; an advice-giving session; a format in which one partner interviews the other and draws out the historical and present context of the partnership.
3. Prepare the case study slides and provide them to the facilitator in advance of the session.
4. Determine if a facilitator or another presenter will be included to introduce presenters, set context, or debrief the session. If additional presenters or small-group facilitators are required, they should be recruited far in advance.

**Roles and Responsibilities During Session**

Presenter

- Your role is to provide an engaging and informative example of the CEnR principle of equitable power and responsibility from the real-world experience you and your partner have had in this area. The above preparatory work will help you guide the conversation, but learners will benefit from any reflections or lessons learned you would share in this area. Learners may or may not have experience in CEnR but, if this has been offered as an optional session, have chosen to engage in this voluntary program indicating an interest in the topic.
- During the large-group component of the session, the focus will be on interaction between community partner and academic partner. Interaction will be encouraged to take place during the small-group breakout sessions following the initial large-group discussion. Presenters may choose to take questions via raised hand or chat box, or they may choose to direct questions to the small-group discussions.
- Following small-group portion of the session, there will be a return to large-group debrief. A takeaway may be requested from each group if time permits. A reminder for the next scheduled session may be provided before the session concludes. If used, a post-session evaluation QR code should be displayed on the final slide and emailed to students following the session.

Small-Group Facilitator

- Small-group facilitators will be responsible for guiding a group of learners through reflection on the community and academic partner discussion, as well as prepared reflective questions. Small-group facilitators may be the community and academic partners who had presented in the session, other community-engaged academic and community leaders, or community-engaged staff or learners. Ideally, small-group facilitators will lead groups of 4-6 learners, although this number may increase or decrease depending on attendance and small-group facilitator recruitment.
- Small-group facilitators are encouraged to first create an atmosphere of openness and comfort. It is recommended to start by asking everyone to lean into the discussion by either putting away their devices (in-person) or turning on their cameras (virtual). A short introduction may be useful, with the small-group facilitator starting by introducing themselves and the reason they have been inspired to join today, then requesting the participants do the same.
- Generate and guide discussion of the learners. Instead of speaking extensively, ask probing questions, invite reflection, and stimulate connection between content, experience, and intention. Learners of CEnR have widely variable levels of experience in the area and can often teach and advise one another, especially on institutionally accessible resources and best practices from a student perspective. Seek to elevate the voices of participants and reinforce and add perspective to them, not to correct or discredit the messages shared.
- If possible, expand the small-group discussion to 50 minutes to enable more thorough and personalized levels of discussion. If this is not possible, it may be necessary to narrow the focus of the questions or areas of discussion posed to the group.
- The prepared questions should not constrain small-group facilitators. Small-group facilitators are invited to provide additional prompts or areas of reflection.

**Post-session Work – Faculty and Staff**

1. Email QR code or link to session evaluation immediately after session.
2. Send an email reminder 2-3 days prior to the next session.

**References**

Ahmed SM, Palermo AG. Community engagement in research: Frameworks for education and peer review. *Am J Public Health.* 2010;100(8):1380-1387.

**Session 3: *Capacity Building and Effective Dissemination***

**Session Agenda (60-90 minutes)**

| **Time** | **Item (Presenter)** |
| --- | --- |
| 5 minutes | Welcome (facilitator, community-engaged researcher and/or clinician)   - Context setting - Presenter introductions |
| 20-25 minutes | CEnR Principles: Capacity Building and Effective Dissemination (community-engaged researcher and/or clinician, student partner[s]) |
| 30-50 minutes | Small-Group Breakout Session (facilitated by presenters; other community-engaged researchers/clinicians/staff)   - Introduction - Facilitated discussion questions   - First 5-10 minutes—please think about and write your response to the prompt:     - *Describe a future self who has reached your potential as a CE physician. What would that look like? What kind of skills would that entail? How would that change the way you were able to impact the health of your patients? How could that be integrated with your clinical and other responsibilities? How would that change the way your patients view you?*   - Remaining time—volunteers share their reflections with the group and discuss the following:     - How might you get from where you are to the future self you describe?     - What/whom might you engage with at your institution toward CE and CEnR capacity building?     - What non-medical school resources might you engage with?     - What resource or opportunity would help you reach your potential as a CE physician, but does not exist at your institution?     - How can we help you reach your goals? |
| 5-10 minutes | Debrief (community-engaged researcher/clinician)   - Breakout group takeaways - Consultation opportunities |

**Materials**

Capacity building and dissemination slides (Appendix F) with facilitator notes

**Session Preparation**

Student

None required

Facilitator

Before attending the session, you should:

1. Determine session duration and adjust agendas as needed.
2. Review slides, inserting program-specific information and presenter-provided materials as needed.

Faculty Presenter

1. Consider a community partner or student who would be suitable to join and assist you in illustrating the dynamics of this CEnR principle. In the past, a senior community-engaged medical student was invited to co-present with their community partner to each share their experience of capacity building and success in dissemination, each from their own perspective. Capacity building and dissemination fit well together in the context of many senior medical learners who have had significant involvement in CEnR. A community partner who could speak to these areas would also be a suitable presenter. Including both a community partner and student who had worked with the faculty member on a CEnR project involving these principles could also be a very engaging triad to consider. It is advisable to identify this community partner and request their assistance far in advance and, if possible, to compensate your community partner for their time in a meaningful way.
2. Meet with your identified co-presenter(s) in advance of this session, in order to determine the most effective way to share your work and illustrate the principles of dissemination and capacity building. There are many possible ways to illustrate this. Suitable options that have been used in the past include an open, reflective discussion; an advice-giving session; a format in which one partner interviews the other and draws out the historical and present context of the partnership; a session focused on reflection and examples of areas of learning or personal growth, discovery, and/or dissemination.
3. Prepare the case study slides and provide them to the facilitator in advance of the session.
4. Determine if a facilitator or another presenter will be included to introduce presenters, set context, or debrief the session. If additional presenters or small-group facilitators are required, they should be recruited far in advance.

**Roles and Responsibilities During Session**

Presenter

- Your role is to provide an engaging and informative example of the CEnR principles of capacity building and effective dissemination from the real-world experience you and your co-presenter(s) have had in this area. The above preparatory work will help you guide the conversation, but learners will benefit from any reflections or lessons learned you would share in this area. Learners may or may not have experience in CEnR but, if this has been offered as an optional session, have chosen to engage in this voluntary program indicating an interest in the topic.
- During the large-group component of the session, the focus will be on interaction between co-presenters. Interaction will be encouraged to take place during the small-group breakout sessions following the initial large-group discussion. Presenters may choose to take questions via raised hand or chat box, or they may choose to direct questions to the small-group discussions.
- Following small-group portion of the session, there will be a return to large-group debrief and series wrap-up. A takeaway may be requested from each group if time permits. A complete series wrap-up will be conducted. If used, a post-session evaluation QR code should be displayed on the final slide and emailed to students following the session.

Small-Group Facilitator

- Small-group facilitators will be responsible for guiding a group of learners through reflection on the community and academic partner discussion, as well as prepared reflective questions. Small-group facilitators may be the community and academic partners who had presented in the session, other community-engaged academic and community leaders, or community-engaged staff or learners. Ideally, small-group facilitators will lead groups of 4-6 learners, although this number may increase or decrease depending on attendance and small-group facilitator recruitment.
- Small-group facilitators are encouraged to first create an atmosphere of openness and comfort. It is recommended to start by asking everyone to lean in to the discussion by either putting away their devices (in-person) or turning on their cameras (virtual). A short introduction may be useful, with the small-group facilitator starting by introducing themself and the reason they have been inspired to join today, then requesting the participants do the same.
- Generate and guide discussion of the learners. Instead of speaking extensively, ask probing questions, invite reflection and stimulate connection between content, experience and intention. Learners of CEnR have widely variable levels of experience in the area and can often teach and advise one another, especially on institutionally accessible resources and best practices from a student perspective. Seek to elevate the voices of participants and reinforce and add perspective to them, not to correct or discredit the messages shared.
- If possible, expand the small-group discussion to 50 minutes to enable more thorough and personalized levels of discussion. If this is not possible, it may be necessary to narrow the focus of the questions or areas of discussion posed to the group.
- The prepared questions should not constrain small-group facilitators. Small-group facilitators are invited to provide additional prompts or areas of reflection.

**Post-session Work – Faculty and Staff**

1. Email QR code or link to session evaluation immediately after session.
2. Send a post-session wrap-up email; certificates of program completion (if desired), and any additional programming and resource materials.

**References**

Ahmed SM, Palermo AG. Community engagement in research: Frameworks for education and peer review. *Am J Public Health.* 2010;100(8):1380-1387.
