# Supplementary material for: Foundations of Community Engagement: A Series for Effective Community-Engaged Research
Source: MedEdPORTAL. 2023 Oct 10;19:11350. doi: 10.15766/mep_2374-8265.11350 (PMC10562524; doi:10.15766/mep_2374-8265.11350)
Supplement: Supplementary file 1 — CE Didactic Session Slides.pptxApplication for Small-Group Series.docxCommunity-Academic Partnership Slides.pptxEquitable Power and Responsibility Slides.pptxEquitable Power and Responsibility Case Studies.docxCapacity Building and Dissemination Slides.pptxFacilitator Guide.docxCE Didactic Session Evaluation.docxSmall-Group Session Evaluation.docx [file mep_2374-8265.11350-s001.zip › H. CE Didactic Session Evaluation.docx]

Virtual Community Engagement Student Summer Series: Didactic Session Evaluation

Thank you for participating in *Community Engagement Principles* offered by the [insert the name of your institution here] in collaboration with [insert name of collaborating program/office/department here].

As a reminder, the session's learning objectives were:

- Review current definitions and principles of community engagement (CE) and community engaged research (CEnR)
- Discuss evolution of and rationale for CE
- Learn about the state of CE at the [insert your institution name here] and how it intersects with other core pillars
- Discuss examples of CEnR research occurring at [insert your institution name here]

Your feedback is important to us! Please respond to the questions below. Your answers will remain anonymous.

Q1 I found this session worthwhile.

- Strongly agree
- Somewhat agree
- Neither agree nor disagree
- Somewhat disagree
- Strongly disagree

Q2 I learned something that I will use in my practice/profession.

- Strongly agree
- Somewhat agree
- Neither agree nor disagree
- Somewhat disagree
- Strongly disagree

Q3 Overall the speakers were effective at communicating session content.

- Strongly agree
- Somewhat agree
- Neither agree nor disagree
- Somewhat disagree
- Strongly disagree

Display this question (Q4) if answer to Q3 is:

If Overall the speakers were effective at communicating session content. = Somewhat agree

Or Overall the speakers were effective at communicating session content. = Somewhat disagree

Or Overall the speakers were effective at communicating session content. = Strongly disagree

Or Overall the speakers were effective at communicating session content. = Neither agree nor disagree

Q4 Please explain how you felt about the speakers:

________________________________________________________________

Q5 On a technical level, how did the platform work for you?

- I had no technical difficulties and was very satisfied with the platform.
- I had no technical difficulties, but I wish the format had been different.
- I had a few technical difficulties, but I was still able to participate fully.
- I had major technical difficulties that limited my ability to participate fully.

Display this question (Q6) if answer to Q5 is:

If On a technical level, how did the platform work for you? = I had no technical difficulties, but I wish the format had been different.

Q6 Please explain what could have been done better:

________________________________________________________________

Display this question (Q7) if answer to Q5 is:

If On a technical level, how did the platform work for you? = I had major technical difficulties that limited my ability to participate fully.

Q7 We are sorry the platform did not work well for you! Please describe the difficulties you had:

________________________________________________________________

Q8 Do you plan to apply for the Virtual Community Engagement Student Summer Series?

- Yes
- No
- Maybe

Q9 Please use the space below to share any other thoughts about the didactic session, including what stood out as beneficial and what could be improved.

________________________________________________________________

Q10 What institution/campus are you from?

- [insert name of your institution here]
- Other

Display this question (Q11) if answer to Q10 is:

If What institution/campus are you from? = Other

Q11 What institution are you from?

________________________________________________________________

Q12 What type of student/learner are you?

- Medical
- Graduate
- Pharmacy
- Resident
- Other

Display this question (Q13) if answer to Q12 is:

If What type of student/learner are you? = Medical

Q13 What year are you in your medical training?

- M1 (1)
- M2 (2)
- M3 (3)
- M4 (4)

Display this question (Q14) if answer to Q12 is:

If What type of student/learner are you? = Graduate

Q14 What degree program are you in?

________________________________________________________________

Display this question (Q15) if answer to Q12 is:

If What type of student/learner are you? = Other

Q15 What type of student/learner are you?

________________________________________________________________

Q16 Are you participating in the summer research program? [IF APPLICABLE TO YOUR INSTITUTION]

- Yes
- No

Thank you for your feedback!
